# Organic Photoredox-Catalyzed TEMPO Disproportionation: Development of a Mild Platform for In Situ Oxoammonium Ion Formation

**DOI:** 10.1021/acs.joc.6c01658

**Published:** 2026-08-04

**Authors:** Caroline W. Evans, Brett N. Hemric

**Affiliations:** Department of Chemistry and Biochemistry, 7832 University of Tampa, Tampa, Florida 33606, United States

## Abstract

The organic photoredox-catalyzed disproportionation of TEMPO (2,2,6,6-tetramethylpiperidine-1-oxyl) is reported with application to the oxidation of alcohols. Although traditional methods of TEMPO disproportionation rely on strong acids, this research provides milder, sustainable conditions using visible light and a weak acid with very small loading (<0.10 mol %) of a common and affordable organic photocatalyst. This method is compatible with acid-sensitive functional groups and applicable to a broad range of primary benzylic, allylic, and aliphatic alcohols with excellent to moderate yields. Detailed mechanistic investigations were employed including Stern–Volmer luminescence quenching analysis, direct UV–vis observation of the fleeting oxoammonium ion intermediate, kinetic isotope effect analysis, and partial rate order determination of the weak acid. These results highlight the key role of the excited photocatalyst in promoting disproportionation with only a small dependence on weak acid. This novel approach expands the applications of TEMPO disproportionation using mild photoredox-catalyzed conditions to bypass the strong acid requirement for greater substrate compatibility while exploring new methodologies for in situ oxoammonium ion generation without the use of external oxidants.

## Graphical abstract

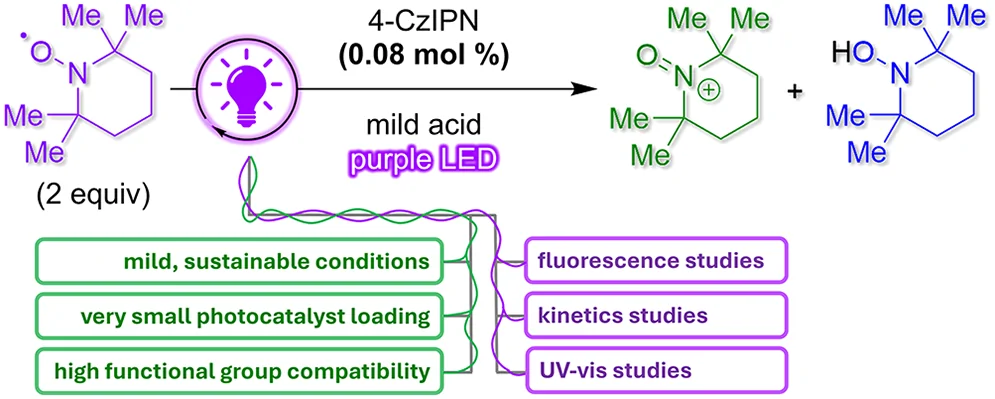

## Introduction

TEMPO (2,2,6,6-tetramethylpiperidine-1-oxyl) is a stable nitroxyl radical that is commonly used for mechanistic analysis as a radical scavenger.^1^ Additionally, TEMPO has received significant interest due to its ability to exist in two additional oxidation states (Scheme 1A):^2^ an oxidized form (oxoammonium ion) and a reduced form (hydroxylamine). In particular, its oxoammonium ion form is widely used within synthetic applications as both an electrophile^3^ and a mild oxidant.^2c^ Although many synthetic methods utilize stoichiometric oxidants to oxidize TEMPO to its oxoammonium ion form, this can also be accomplished through the disproportionation of two TEMPO radicals to achieve both the oxoammonium ion and hydroxylamine forms (Scheme 1B). This disproportionation is traditionally driven by a strongly acidic medium of pH < 2;^4^ however, the corrosive nature of these strong acids creates harsh conditions that are incompatible with acid-sensitive substrates. As such, an increasingly mild strategy toward the disproportionation of TEMPO would be highly desired.

**Scheme 1:**
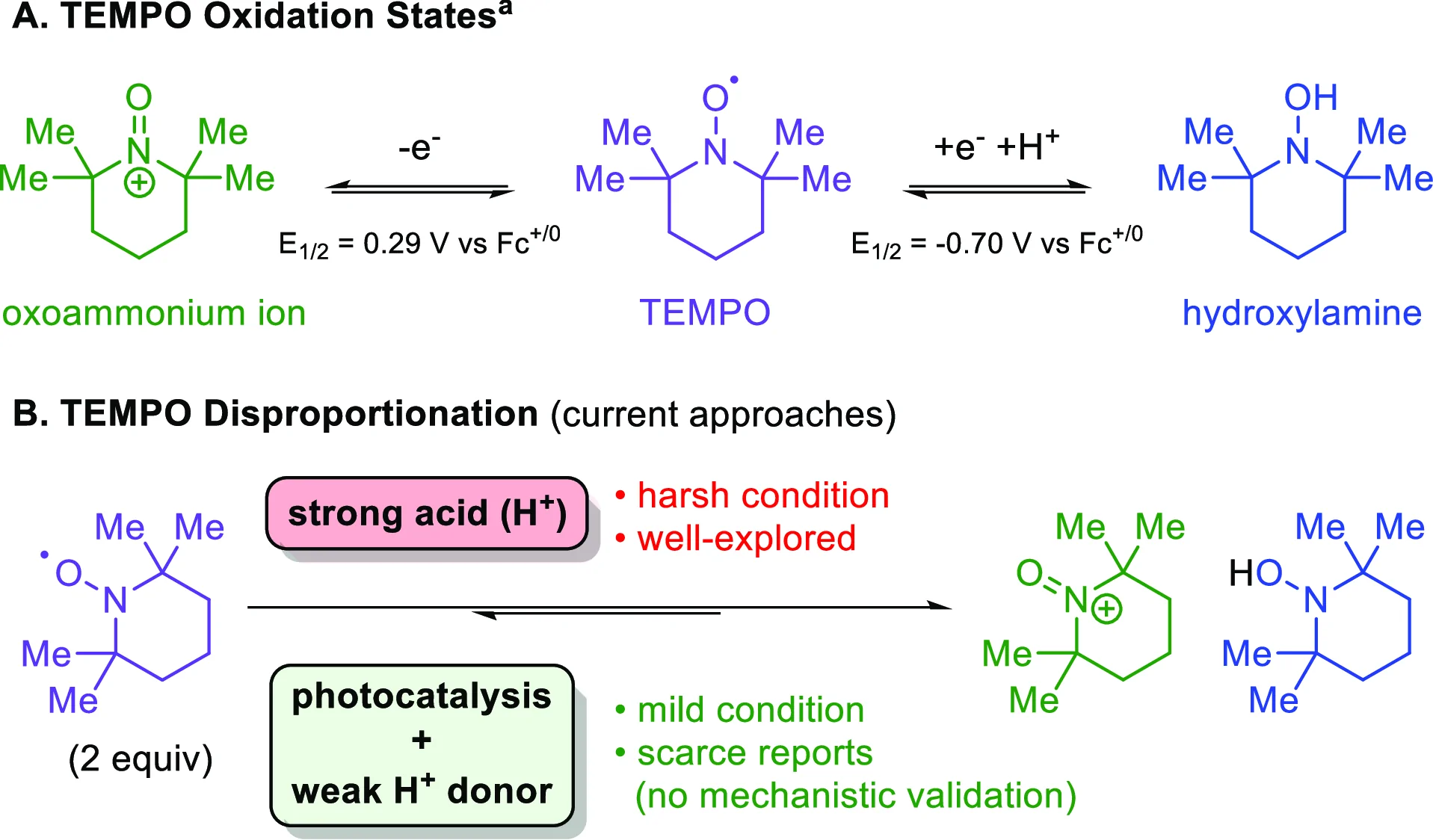
TEMPO Oxidation States and Disproportionation

The application of visible light in organic synthesis is a powerful strategy for accomplishing chemical transformations under mild conditions.^5^ Photoinduced single-electron transfer (SET), termed photoredox catalysis, utilizes a chromophore’s excited redox potential for radical activation. Modern photocatalysts utilize visible light with high efficiency to bypass the chemical degradation associated with high-energy ultraviolet light.^6^ Although traditional approaches have leveraged heavy metal photocatalysts due to their strong visible light absorption, long-lived excited states, and tunable redox potentials, focus has shifted toward economical and earth-abundant alternatives,^7^ such as organic dyes, which are prized for their homogeneity, chemical stability, and comparable redox windows.^8^ Hence, the application of photoredox catalysis to TEMPO disproportionation would offer a significantly more mild and compatible condition than previous acid-driven strategies.

The application of photoredox catalysis to TEMPO disproportionation has been proposed by Koike and Akita^9^ in a process that utilizes an iridium photocatalyst to facilitate TEMPO disproportionation toward the alpha-oxygenation of 1,3-dicarbonyl compounds (Scheme 2A). However, a very similar transformation reported by Tan et al.^10^ with Rose Bengal proposes a mechanism independent of TEMPO disproportionation. Neither of these proposals are supported by any clarifying mechanistic investigation, which brings into question the underlying mechanism for this process and merits more comprehensive analysis.^11^ Some reports exist for TEMPO participating in photocatalytic reactions but only catalytically or in the presence of stoichiometric oxidants.^12^ This report seeks to pair the development of a photoredox-catalyzed TEMPO disproportionation with a thorough investigation of the mechanism. This would bypass the traditional requirement for strong acids and expand the applications of photocatalysis while also clarifying existing mechanistic incongruities (Scheme 2B).

**Scheme 2:**
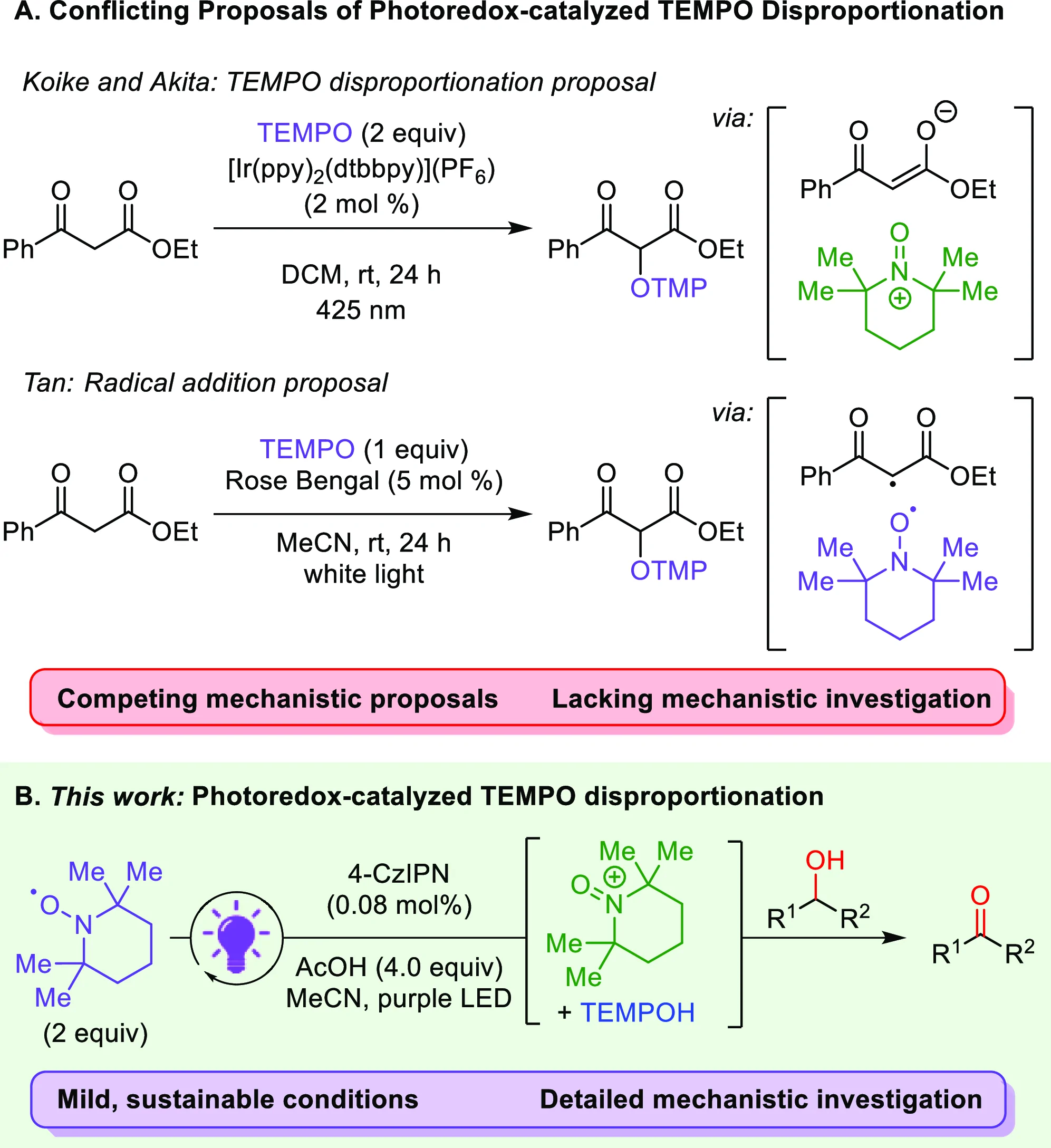
Discrepancies in the Existing Reports of Photoredox-Catalyzed TEMPO Disproportionation and a New System

a Electrochemical data were obtained from the literature.^2f,13^

Herein, we report the development of photoredox-catalyzed TEMPO disproportionation applied to an alcohol oxidation system featuring compatibility with acid-sensitive substrates and mild, sustainable conditions. This process proceeds efficiently with extremely small loadings of a widely available organic dye using an inexpensive visible-light system at room temperature. Detailed mechanistic investigations support the theory of photoredox-catalyzed TEMPO disproportionation driven by light energy, successfully bypassing the previous requirement of a strong acid.

## Results and Discussion

The photocatalyst 4-CzIPN (1,2,3,5-tetrakis­(carbazol-9-yl)-4,6-dicyanobenzene) (Scheme 3) was chosen for this reaction as a widely available and affordable organic dye known for its high catalytic efficiency and strong excited redox window (*E*_1/2_^PC^•+^/PC*^ = −1.16 V vs SCE and *E*_1/2_^PC*/PC^•–^^ = +1.50 V vs SCE).^14^ Acetonitrile (MeCN) was chosen for its ability to stabilize charged species and support electron transfer events.^15^ Acetic acid (AcOH) was tested as a mild acid (p*K*_a_ 4.76 in H_2_O, 23.5 in MeCN)^13a^ that would not promote TEMPO disproportionation in the dark.

**Scheme 3:**
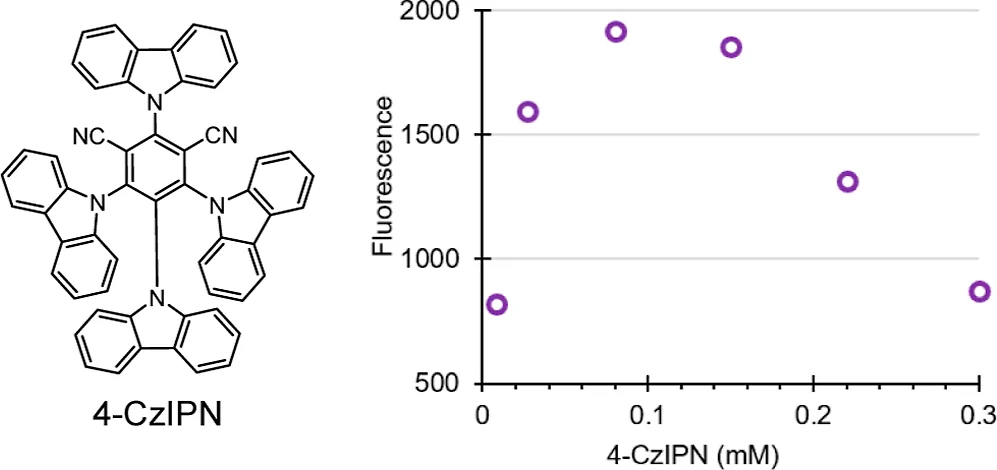
Fluorescence for 4-CzIPN at 400 nm to Determine Optimal Loading and Avoid Photocatalytic Self-Quenching^a^

a Solutions in MeCN. Emission measured at 450–650 nm.

To glean photophysical insight on the optimal photocatalyst loading, its fluorescence properties were measured. 4-CzIPN was found to have a local maximum absorbance peak (λ_max_) around 365 nm with decreasing absorbance farther into the visible region (see Supporting Information Figure S1 for the absorbance spectrum). First, it was noted that the emission intensity of 4-CzIPN was similar for both 365 and 400 nm (see Supporting Information Figure S3). Accordingly, the fluorescence of 4-CzIPN was measured with excitation at 400 nm at various concentrations to test for the optimal loading to avoid self-quenching (Scheme 3). Fluorescence initially rose with increased photocatalyst loadings as optimal fluorescence occurred with 80 μM 4-CzIPN, which corresponds to 0.08 mol % photocatalyst loading for a reaction mixture with 0.1 M alcohol. The steady decrease in fluorescence intensity with 4-CzIPN concentrations following 80 μM is rationalized by aggregation-induced self-quenching of the photocatalyst from intermolecular π–π stacking which inhibits photonic absorption and increases nonradiative relaxation pathways.^16^

Accordingly, optimization began with 0.08 mol % 4-CzIPN with 4-biphenylmethanol (**1a**) as the model alcohol along with 4 equiv of AcOH and 2 equiv of TEMPO (**2a**) in MeCN and an argon atmosphere at room temperature (Table 1, entry 1). Ultimately, these conditions were also found to be the optimal conditions. First, it was established that a slight background reaction occurred in the dark, indicating that some oxidation occurs independent of photocatalysis (entry 2). Next, these conditions were compared to the wavelengths previously tested in the emission spectra to correlate product yields against the fluorescence data. This demonstrated 400 nm as the optimal excitation wavelength (entries 3–4). The decreased yields at 365 nm were hypothesized to be caused by absorbance from **1a** itself from its extended conjugation. Gratifyingly, the small 0.08 mol % photocatalyst loading that previously provided optimal emission intensity also provided optimal product yields when compared to higher, more traditional photocatalyst loadings (entries 6–8). Furthermore, only a slight background reaction was observed in the absence of the catalyst (entry 5). Additionally, varying the amount of AcOH confirmed 4 equiv of AcOH as optimal for this reaction (Table 1, entries 9–12), with the complete absence of AcOH mostly eliminating reactivity (entry 9). When the amount of TEMPO was surveyed, 2 equiv was found to be optimal (entries 13–15), with the absence of TEMPO still providing a slight background reaction (entry 13). Notably, the diminished yields for TEMPO amounts below 2 equiv supports the idea of TEMPO disproportionation, as only half of the TEMPO present can provide an oxidation product. (Note that 1 equiv of TEMPO is likely above 50% due to some background reaction, as seen with 0 equiv in entry 15). Finally, a decrease in reaction yield is observed under an ambient atmosphere (entry 16) which is rationalized by the T_1_ quenching abilities of molecular oxygen.^17^ Accordingly, it was determined that all components are vital to providing the desired reaction in high yields, with only slight reactivity observed when one component is absent (entries 2, 5, 9, and 13).

**Table 1:**
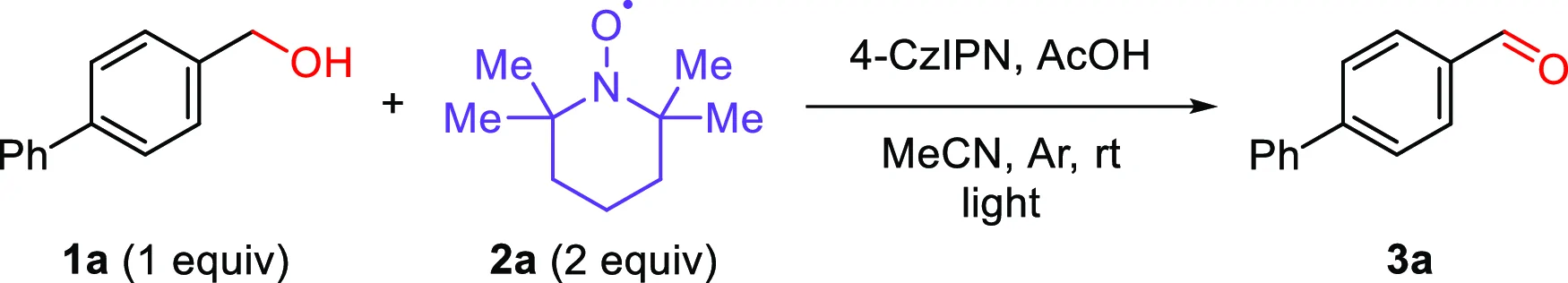
Reaction Optimization^a^

entry	TEMPO (equiv)	AcOH (equiv)	4-CzIPN (mol %)	λ (nm)	3a (%)^b^
1	2	4	0.08	400	95 (83)^c^
2	2	4	0.08	dark	9
3	2	4	0.08	365	44
4	2	4	0.08	465	66
5	2	4	0	400	11
6	2	4	1	400	87
7	2	4	3	400	94
8	2	4	5	400	94 (87)^c^
9	2	0	0.08	400	8
10	2	2	0.08	400	88
11	2	3	0.08	400	80
12	2	5	0.08	400	91
13	0	4	0.08	400	6
14	1	4	0.08	400	62
15	1.5	4	0.08	400	75
16^d^	2	4	0.08	400	80

a Reactions were carried out on a 0.1 mmol scale.

b Reaction yields were determined by ^1^H NMR of the crude reaction with dibromomethane as a quantitative internal standard.

c Isolated yield on a 0.3 mmol scale with 3.9 equiv of AcOH in parentheses.

d The reaction was run under ambient air.

With optimized conditions in hand, the alcohol substrate scope for this reaction was evaluated (Table 2). Notably, 4-phenylbenzaldehyde (**3a**) was found to provide a slightly higher yield at the 1.0 mmol scale. For other benzaldehyde products, the deuterated 4-phenylbenzaldehyde worked with a slightly lower efficiency to the proton analog (**3a′** vs **3a**). Naphthaldehydes of both 1- and 2-substitution were viable, with the 1-naphthaldehyde giving higher yield, opposite of what might be supposed from steric effects (**3b**–**3c**). Notably, this reaction worked on protected alcohols (despite the potentially deprotecting presence of AcOH) such as a catechol-protected diol (**3d**) as well as a silane-protected alcohol (**3e**), thus demonstrating the compatibility of this method with common alcohol protection strategies. A number of other functional groups were tolerated within the reaction, including carboxylic acids (**3f**), esters (**3g**), and sulfides (**3h**), with no oxidation observed on the latter substrate. However, the production of vanillin (**3i**) resulted in poor yield, a phenomenon that can be attributed to chelation by the para-hydroxyl substituent with the oxoammonium ion, as reported by Stahl.^18^ A range of cinnamyl alcohols were also tested, with all substrates providing elevated yields with reasonably high *E*/*Z* ratios (**3j**–**3n**). Both electron-donating and electron-withdrawing substituents provided high yields (**3**k/**3m** vs **3l**), and steric elements on the system did not prove to be deleterious (**3n**). Similarly, both geranial (**3o**) and farnesal (**3p**) were produced in the reaction, with high yield but 1:1 *E*/*Z* ratio. Notably, it has been reported that small-molecule photocatalysts can induce *E*/*Z* isomerization of alkenes by performing energy transfer from the excited triplet state.^19^ Limited aliphatic cases were tested but resulted in excellent to good yields (**3q**–**3r**). Unfortunately, only select secondary alcohols were compatible with this reaction, producing minimal reaction yields (**3s**–**3t**). This agrees with previously reported oxidations of secondary alcohols by oxoammonium ions.^18,20^ Notably, no ring-opened product was observed in the production of **3t**, suggesting the oxidation step in the mechanism does not involve a radical intermediate (in agreement with previous reports).^21^

**Table 2:**
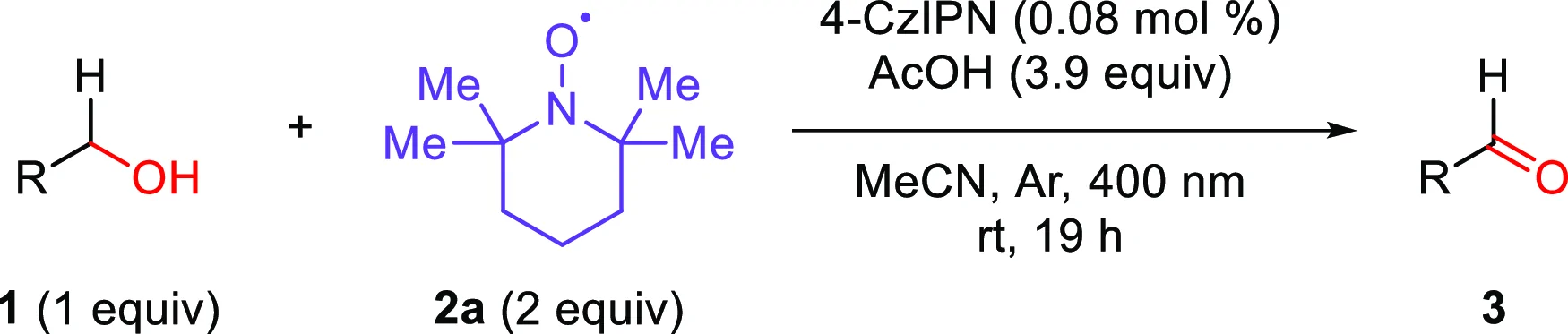

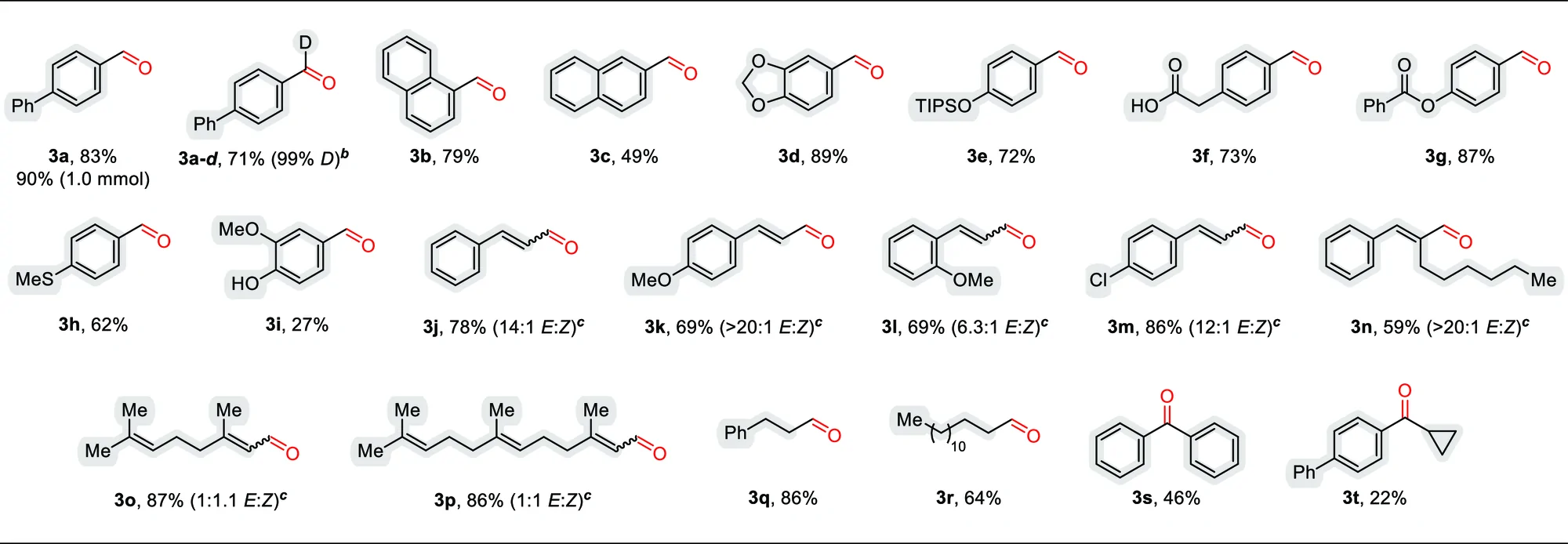
Alcohol Scope for the Photoredox-Catalyzed Oxidation Reaction^a^

a Isolated yields were obtained on a 0.3 mmol scale.

b Starting from the *d*_2_-alcohol (99% *D*).

c Ratio determined by ^1^H NMR of the crude reaction starting from the corresponding >20:1 *E*/*Z* alcohols.

In examining the reaction mechanism, attempts were made to experimentally verify the occurrence of TEMPO disproportionation through direct observation of the oxoammonium ion intermediate formed in the reaction (Scheme 4A). An experiment was devised to trap the oxoammonium ion intermediate in an aqueous phase to isolate it from its precursor radical and hydroxylamine forms to avoid the thermodynamically favorable comproportionation. As this oxoammonium salt is strongly water-soluble, unlike its radical and hydroxylamine forms, MeCN was substituted for denser 1,2-dichloroethane with water to create a biphasic mixture. Upon 400 nm irradiation for 4 h with AcOH and 4-CzIPN, the aqueous phase was immediately separated and measured with UV–visible spectroscopy. The resulting UV absorption spectrum of the aqueous phase strongly agreed with the spectrum of the oxoammonium ion with possible trace amounts of residual TEMPO (Scheme 4B). This direct observation of the trapped oxoammonium ion under photocatalytic conditions provides empirical evidence for the occurrence of TEMPO disproportionation. Therefore, it was concluded that the formation of the oxoammonium ion via TEMPO disproportionation was responsible for alcohol oxidation.

**Scheme 4:**
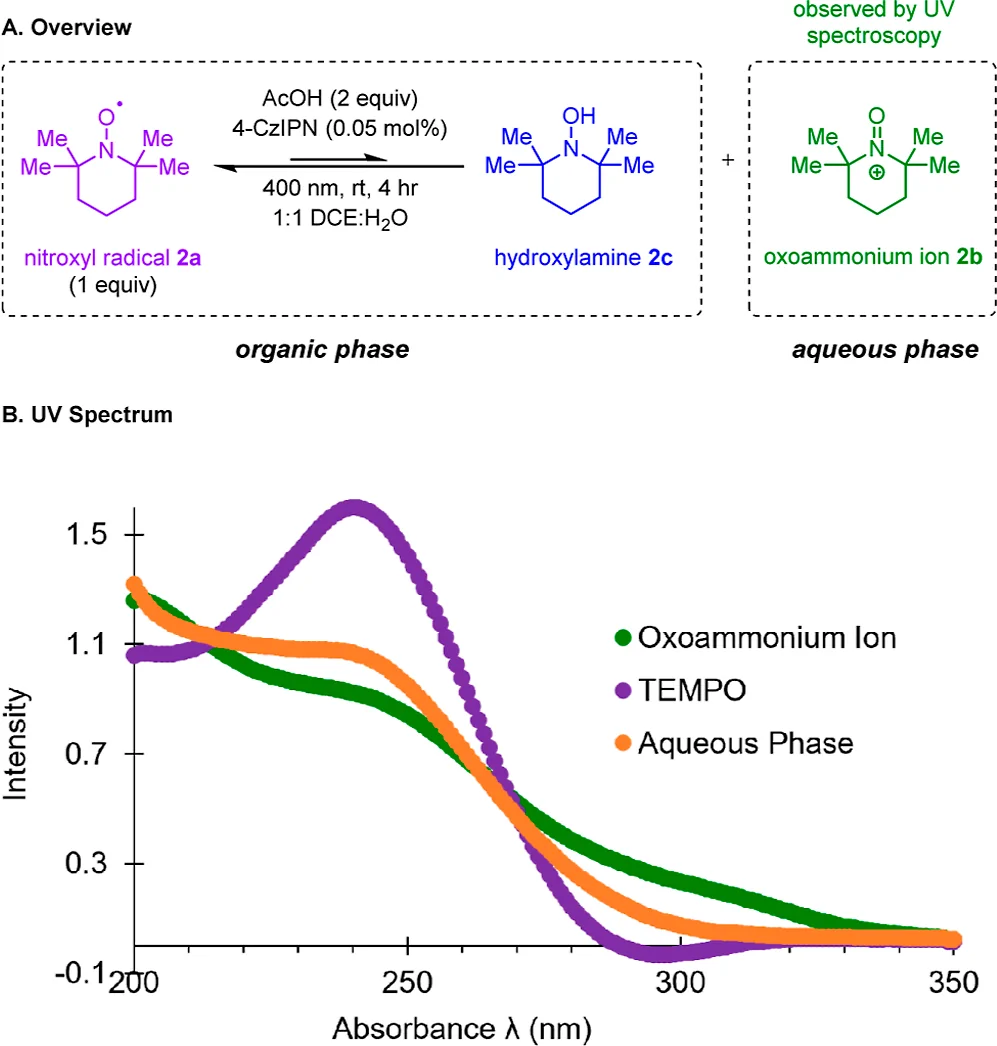
Oxoammonium Trapping via Disproportionation into the Aqueous Phase

In order to further implicate the importance of light irradiation in the reaction, a light on/off experiment was conducted by monitoring the formation of product **3a** under normal reaction conditions with aliquots taken at the start and end of alternative on/off light cycles (Scheme 5). Noticeable reaction progress was observed during the 20 min periods of light irradiation (0–20 min, 40–60 min, and 80–110 min). Agreeing with previous control experiments, only a very small background reaction was observed in the 20 min dark periods (20–40 min and 60–80 min). It was concluded that consistent light exposure was necessary for reaction progress and hypothesized that a light-initiated cascade/relay process was unlikely.

**Scheme 5:**
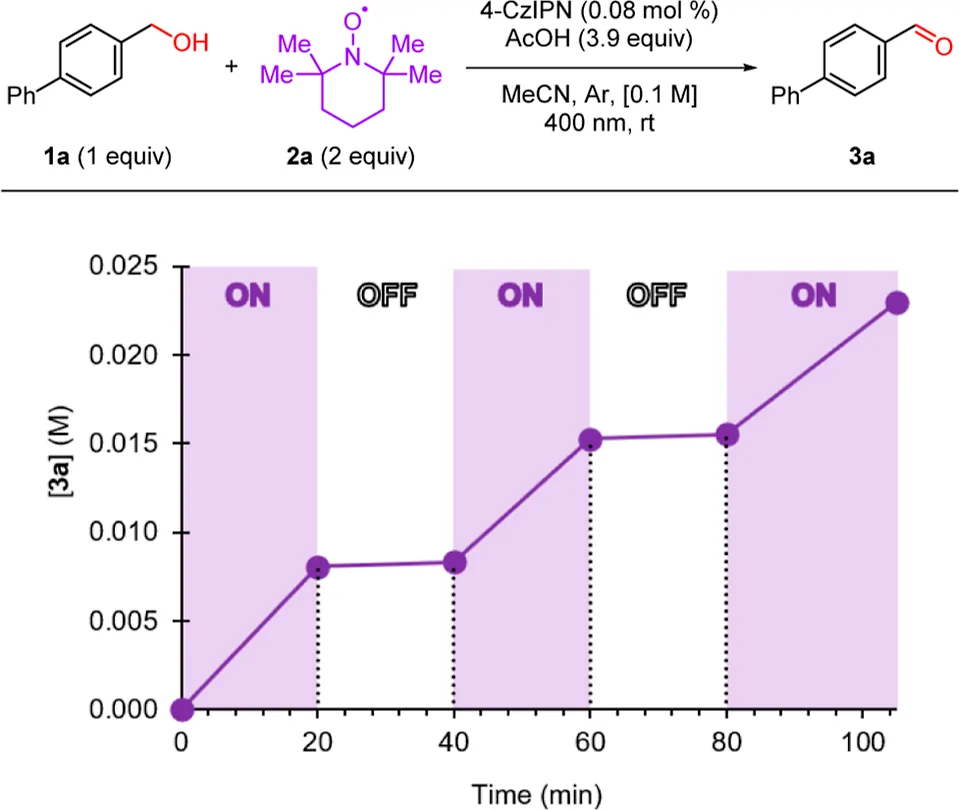
Light On/Off Experiment

a Reactions were carried out on a 0.3 mmol scale with 1,1,2,2-tetrachloroethane (0.3 mmol) as a quantitative internal standard.
b Product amounts were determined by solvent-suppressed ^1^H NMR of CDCl_3_-diluted (0.3 mL) aliquots (0.20 mL) at the indicated times.

After concluding that TEMPO disproportionation is indeed occurring and that the reaction is driven by light energy, Stern–Volmer luminescence quenching analysis was conducted to investigate the mechanistic role of the photocatalyst and TEMPO. Emission spectroscopy demonstrated that fluorescence intensity decreased significantly with increased concentrations of TEMPO (see Supporting Information Figure S5). A Stern–Volmer quenching plot was assembled as a plot of this luminescence quenching vs TEMPO concentration (Scheme 6).^22^ Significant luminescence quenching was observed with an S_1_ kinetic quenching constant calculated as *k*_s_ = 1.47 × 10^10^ M^–1^ s^–1^ (see Supporting Information, Section 6C). These results demonstrate that rapid S_1_ quenching of 4-CzIPN from the excited state by TEMPO is the driving force behind its disproportionation.^22^ Based on the reported mechanisms of 4-CzIPN in photocatalysis,^23^ it is reasonable to surmise that the strong photoexcited redox potentials of 4-CzIPN allow it to act as an electron shuttle to perform two sequential, nonspontaneous redox events to catalyze its disproportionation.

**Scheme 6:**
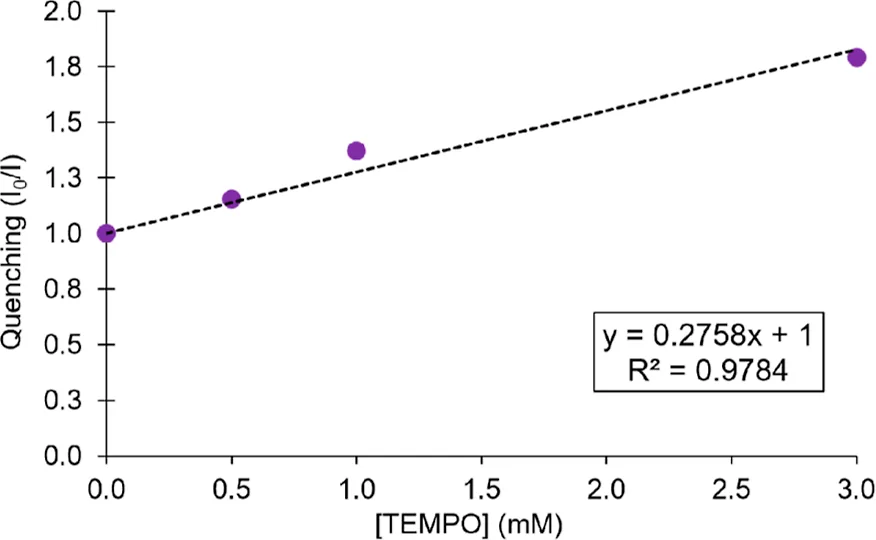
Stern–Volmer Analysis of TEMPO as a Quencher of 4-CzIPN

To further eliminate the possibility that AcOH could be driving TEMPO disproportionation, the rate order for AcOH was determined through kinetic analysis. While AcOH is too weak to independently drive disproportionation, it was hypothesized that AcOH was necessary to promote the kinetics of photocatalyzed disproportionation by protonating the hydroxylamine form and slowing the competitive comproportionation. The reaction rate was graphed against the concentration of AcOH and modeled as a power fit (Scheme 7). From this, the partial reaction order with respect to AcOH was determined to be 0.52 (see Supporting Information, Section 8). This substoichiometric partial rate order indicates an indirect mechanistic role of AcOH. Furthermore, these results demonstrate that photocatalyzed TEMPO disproportionation differs from traditional methods in that it is not driven by acid protonation.

**Scheme 7:**
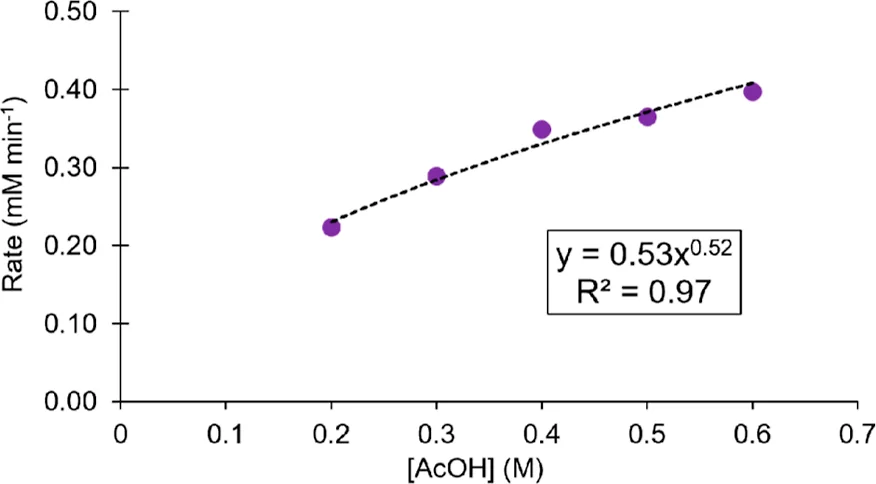
Partial Rate Order in Acetic Acid

The lack of irreversible oxoammonium ion buildup in solution for this reaction when alcohol is removed suggests a reversible comproportionation step. Hence, we theorize the mechanistic role of AcOH is mainly stabilization of the reduced hydroxylamine anion by protonation to slow the competing comproportionation process. This would increase the lifetime of the oxoammonium ion intermediate to kinetically allow its interaction with the alcohol substrate. Additionally, excess weak acid could shift the reaction equilibrium to favor TEMPO disproportionation as described by Le Chatelier’s Principle. It is also possible that AcOH interacts with the TEMPO radical prior to the initial TEMPO oxidation event through hydrogen-bonding or direct protonation to reduce the endergonic barrier of disproportionation, further kinetically promoting this step. These rationalizations account for both the necessity of AcOH in the reaction mechanism and its substoichiometric partial rate order.

A kinetic isotope effect (KIE) study was performed to investigate the kinetics of the alcohol oxidation step in the mechanism (Scheme 8). Our findings show a KIE of 1.32, which aligns with prior reports suggesting that alcohol oxidation represents the turnover-limiting step.^24^ This KIE study and the reversibility of disproportionation suggest that the reaction becomes irreversible only upon alcohol oxidation by the oxoammonium ion.

**Scheme 8:**
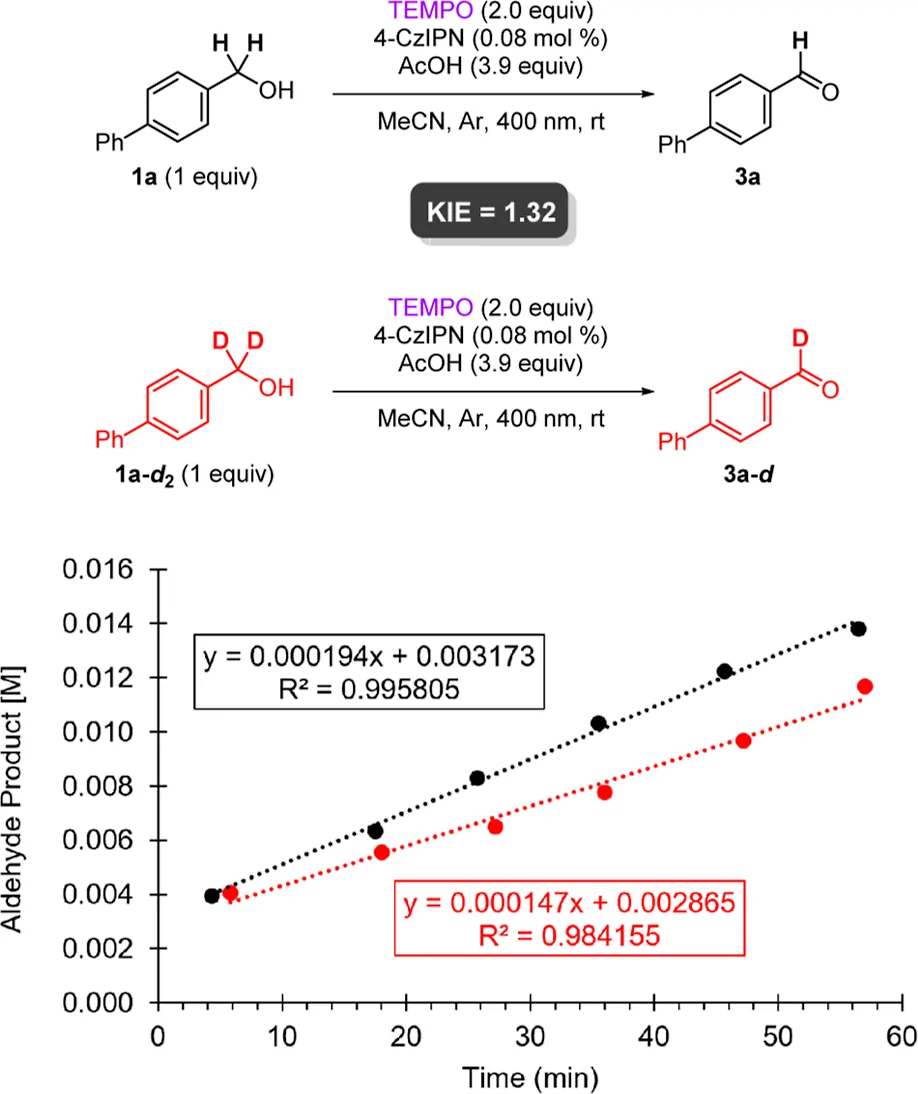
Kinetic Isotope Effect

a Reactions were run on a 0.3 mmol scale with 1,1,2,2-tetrachloroethane as a quantitative internal standard.

To unify the previous analysis, a proposed mechanism can be formulated (Scheme 9). First, initial photocatalytic excitation of 4-CzIPN (A) followed by SET with TEMPO (B) creates the oxoammonium ion and a radical anion form of 4-CzIPN. While photoexcited 4-CzIPN possesses bifunctional redox capabilities, its higher oxidative potential coupled with the more facile TEMPO oxidation suggests a more favorable reductive quenching pathway. The photocatalyst is then regenerated back to the ground state through oxidation by a second TEMPO radical to yield its hydroxylamine anion (C), which is stabilized by AcOH protonation (D) (as mentioned previously, it is possible that AcOH can loosely coordinate to the TEMPO radical to promote the TEMPO reduction in step C). Finally, an irreversible and rate-limiting alcohol oxidation by the photochemically generated oxoammonium ion (E) furnishes the aldehyde and TEMPO hydroxylamine. This process occurs in competition with comproportionation of the oxoammonium ion and hydroxylamine anion.

**Scheme 9:**
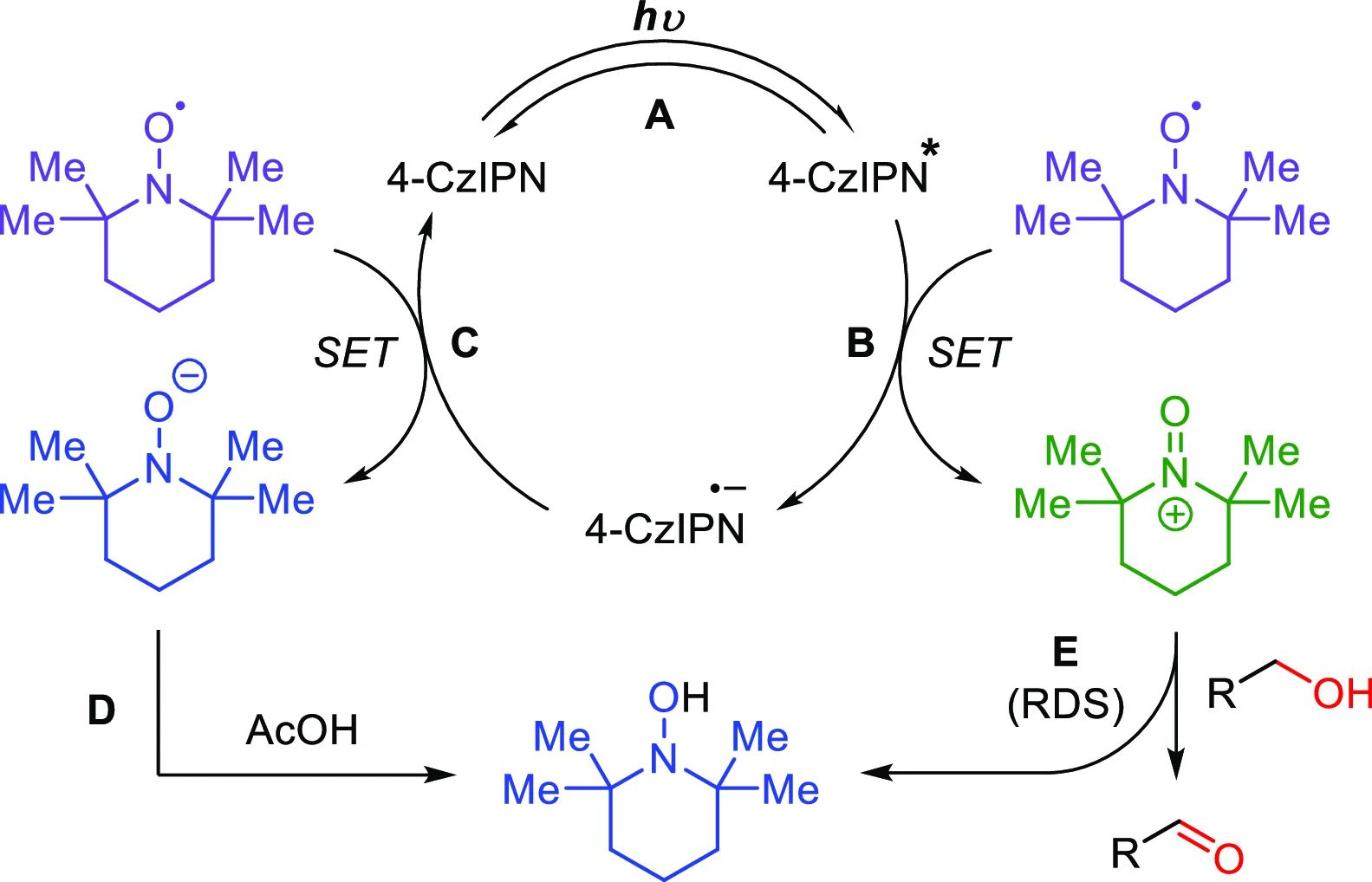
Proposed Mechanism

## Conclusion

This work describes the development of photoredox-catalyzed TEMPO disproportionation applied to the oxidation of alcohols. This process promotes mild and sustainable chemistry using visible light and very small loadings of an affordable organic dye. In addition to demonstrating a substrate scope with high yields and functional group tolerance, significant mechanistic analyses were performed. A small partial reaction rate order for AcOH (0.52) determined from kinetic analysis, combined with significant photocatalytic quenching by TEMPO (*k*_s_ = 1.47 × 10^10^ M^–1^ s^–1^) determined from Stern–Volmer luminescence quenching analysis, suggests a reaction mechanism driven by the excited redox potential of the photocatalyst instead of the p*K*_a_ of a strong acid. UV–vis studies demonstrated the presence of the oxoammonium ion under the reaction conditions, and an alcohol KIE of 1.32 suggests alcohol oxidation is the rate-limiting step in this mechanism. Future research seeks to explore alternative applications of the photochemically generated oxoammonium ion as an electrophile in nucleophilic addition reactions.

## Supplementary Material



## Data Availability

The data underlying this study are available in the published article and its Supporting Information.

## Notes

Experimental procedures and full characterization data for all new compounds with copies of ^1^H, ^13^C, and ^19^F spectra (PDF)
