# Supplementary material for: Organic Photoredox-Catalyzed TEMPO Disproportionation: Development of a Mild Platform for In Situ Oxoammonium Ion Formation
Source: J Org Chem. 2026 Aug 4;91(32):11186–92. doi: 10.1021/acs.joc.6c01658 (PMC13488434; doi:10.1021/acs.joc.6c01658)
Supplement: Supplementary file 1 [file jo6c01658_si_001.pdf]

Organic Photoredox-Catalyzed TEMPO Disproportionation: Development of a Mild Platform for In Situ Oxoammonium Ion Formation

Caroline W. Evans and Brett N. Hemric*

Department of Chemistry and Biochemistry, University of Tampa, Tampa, FL 33606

Email: bhemric@ut.edu

SUPPORTING INFORMATION

Table of Contents **Page**

1. General Methods S1
2. Condition Optimization S2
3. Synthesis of Starting Materials S4
4. Protocol and Characterization of Novel Compounds S7
5. UV-Visible Spectroscopy Studies S13
   A. 4-CzIPN Absorption Spectrum
   B. Oxoammonium Trapping Experiment
6. Fluorescence Spectroscopy Studies S14
   A. 4-CzIPN Emission Spectrum
   B. Photocatalytic Self-Quenching
   C. Stern-Volmer Luminescence Quenching Analysis
7. Light On/Off Experiment S17
8. Determination of Partial Reaction Rate Order in Acetic Acid S18
9. Kinetic Isotope Effect S22
10. References S24
11. ^{1}H and ^{13}C NMR Spectra S25

1. General Methods

General Procedures

Stir bars were cleaned stirring in concentrated nitric acid overnight, rinsed with water and acetone, dried in an oven at 120 °C overnight and cooled/stored in a desiccator filled with Drierite. Optimization and substrate screens were performed in 1- or 2-Dram glass vials with Teflon-coated micro stir bar. All other reactions were performed in round-bottom flasks with rubber septa and Teflon-coated stir bars, unless otherwise noted. Plastic syringes were used for the transfer of pure solvents and glass pipets were used for transfer of crude reaction solutions. Analytical thin-layer chromatography (TLC) was performed using aluminum plates coated with a 0.25 mm layer of 230–400 mesh silica gel with fluorescent indicator (254 nm). TLC plates were visualized by exposure to ultraviolet light and treatment with vanillin or $KMnO_4$ stain. Organic solutions were concentrated under reduced pressure using a rotary evaporator. Flash column chromatography was performed using 60 Å silica gel or a Teledyne ISCO Combiflash RF+ with RediSep Gold Silica Gel Flash Columns.

Materials

Commercial reagents and anhydrous solvents were used as received.

Instrumentation

Nuclear magnetic resonance spectra were recorded on a JEOL 400 MHz cryoprobe at room temperature unless otherwise noted. Chemical shifts for ^{1}H NMR are reported in parts per million (ppm, δ) and referenced to residual protium in $CDCl_3$ (δ 7.26). Chemical shifts for ^{13}C NMR are reported as total carbons in parts per million (ppm, δ) and referenced to the carbon resonances of $CDCl_3$ (δ 77.0) unless otherwise noted. NMR values are reported as follows: chemical shift, multiplicity (s = singlet, d = doublet, t = triplet, q = quartet, p = pentet, m = multiplet, br = broad), coupling constant (Hz), and integration. Infrared spectroscopic data was obtained on a Thermo Nicolet iS10 FTIR and is reported in wavenumbers (cm^{-1}). High-resolution mass spectra of compounds were obtained using a Thermo Scientific Orbitrap Exploris 120 electrospray ionization mass spectrometer, introduced through direct injection (10 or 100 µL/min flow rate, 80 psi nitrogen gas curtain). UV-Vis spectra were obtained on an Agilent Cary 300 double-beam UV-Vis spectrophotometer using semi-microscale quartz cuvettes. Fluorescence spectra were obtained on an F-7000 FL Spectrophotometer using 3.5 mL 10 mm quartz cuvettes. Melting point values were obtained on a DigiMelt MPA160.

The photoirradiation setup for reactions was assembled using two 100 W, 395-400 nm LED panels purchased from "Indmird" on Amazon (https://a.co/d/05F7k5MA). The panels were placed parallel to each other (ca. 2 inches apart) with the borosilicate glass vials (Chemglass product #CG-4904-06 and CG-4904-09) suspended ca. 1 inch from each plate via copper wire from a rod extended between/above the plates. No light filters were utilized. A 10-inch diameter fan on high setting was placed at the end of the setup to allow the air to flow between the plates. Temperature analysis revealed the air between the plates to be maintaining room temperature.

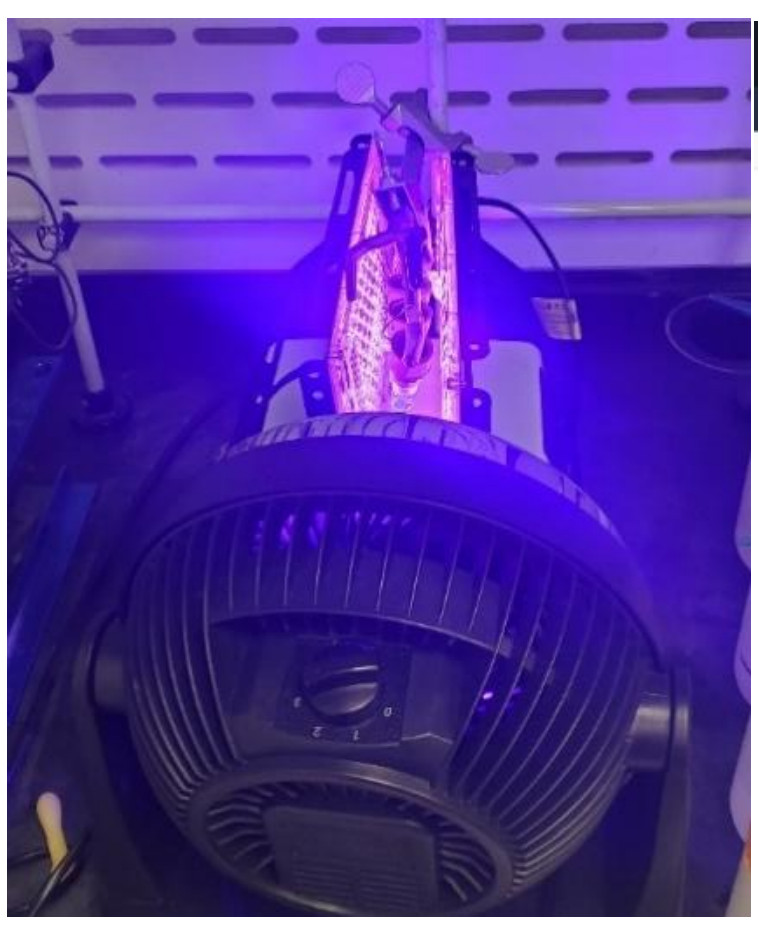

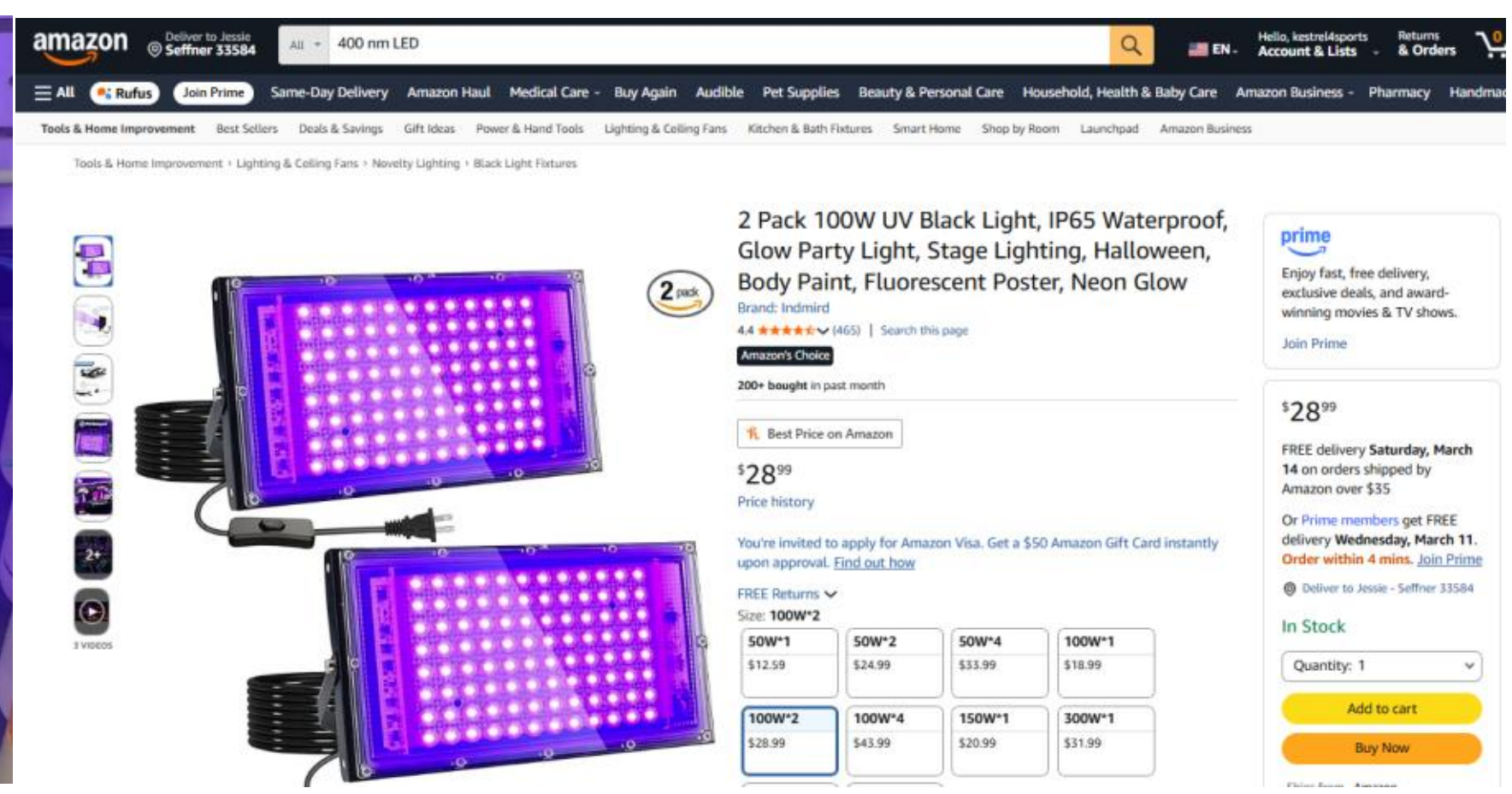

2. Condition Optimization

General Example of Optimization Screening Conditions

To a 1-dram vial equipped with a Teflon-coated stir bar was added TEMPO **2a** (31.3 mg, 0.2 mmol, 2 equiv) and 4-biphenylcarboxaldehyde (18.4 mg, 0.1 mmol, 1 equiv). The vial was capped with a rubber septum and sealed with parafilm. Using a needle inserted through the rubber septum, air was evacuated and the vial was backfilled with argon three times. Under argon using a 1 mL syringe, dry MeCN (0.92 mL) and a stock solution of 4-CzIPN (0.96 mM in MeCN, 83.0 µL, 0.00008 mmol, 0.0008 equiv) were added (this creates an 80 µM 4-CzIPN solution when combined with 0.92 mL MeCN) followed by glacial acetic acid

(23.1 µL, 24.0 mg, 0.4 mmol, 4 equiv). The vial was stirred 800 rpm for 19 h under 400 nm irradiation of (2x 100 W, 400 nm LED panels, 1" from vial). Room temperature was maintained using a circulating fan. The resulting crude mixture was quenched by removal of 400 nm light source and exposure to air, then concentrated *in vacuo* to yield the crude product. Yields were determined by quantitative ^1H NMR spectroscopy through addition of $CDCl_3$ (0.5 mL) and dibromomethane (7.0 µL, 0.1 mmol via a 10 µL microsyringe) to the crude reaction. The resulting solution was analyzed by ^1H NMR with a 45° pulse angle and 4 second relaxation delay with 16 scans. The resulting spectra were analyzed in MestReNova, with the dibromomethane singlet peak (found at 4.905 ppm) set to a relative integration of 2.0.

Table S1. Condition Survey for Alcohol Oxidation[a]

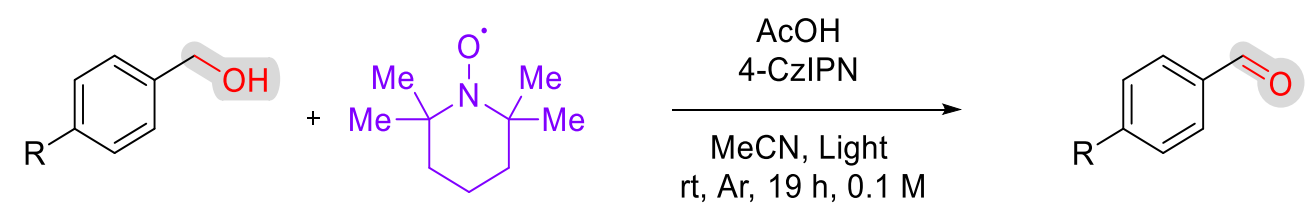

1, 1 equiv + **2,** 2 equiv → **3**

entry	R	**TEMPO** (equiv)	**AcOH** (equiv)	**4-CzIPN** (mol%)	**Wavelength** (nm)	**3a** (%)[b]
1	Ph	2	4	0.08	365	44
2	Ph	2	4	0.08	400	82
3	Ph	2	4	0.08	465	66
4	Ph	2	4	0.08	dark	9
5	Ph	2	0	0.08	400	8
6	Ph	2	2	0.08	400	88
7	Ph	2	3	0.08	400	80
8	Ph	2	4	0.08	400	95
9	Ph	2	5	0.08	400	91
10	CF_3	2	6	0.08	dark	5
11	Ph	0	4	0.08	400	6
12	Ph	1	4	0.08	400	62
13	Ph	1.5	4	0.08	400	75
14	Ph	2	4	0	400	11
15	Ph	2	4	5	400	94 (87)[c]
16	Ph	2	4	3	400	94
17	Ph	2	4	1	400	87
18	Ph	2	4	0.08	400	95 (83)[c]
19[d]	Ph	2	4	0.08	400	80

[a]Run on 0.1 mmol scale. [b]Determined with quantitative ^1H NMR with dibromomethane (7.0 µL, 0.1 mmol). [c]Isolated yields on a 0.3 mmol scale. [d]Reaction conducted in an aerobic atmosphere.

3. Synthesis of Starting Materials

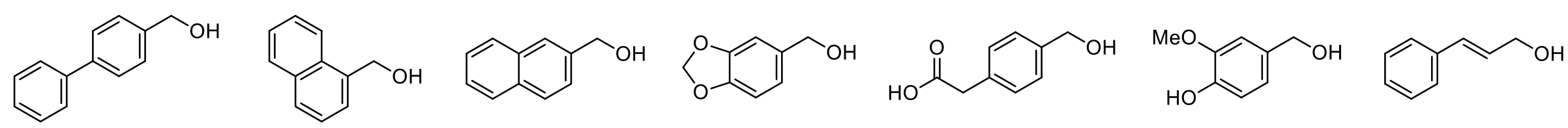

1a 1b 1c 1d 1f 1i 1j

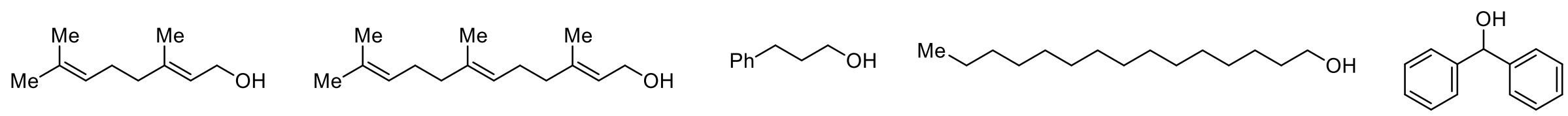

1o 1p 1q 1r 1s

Substrates **1a**, **1b–d**, **1f**, **1i–j**, and **1o–s** were purchased from commercial sources and used as received.

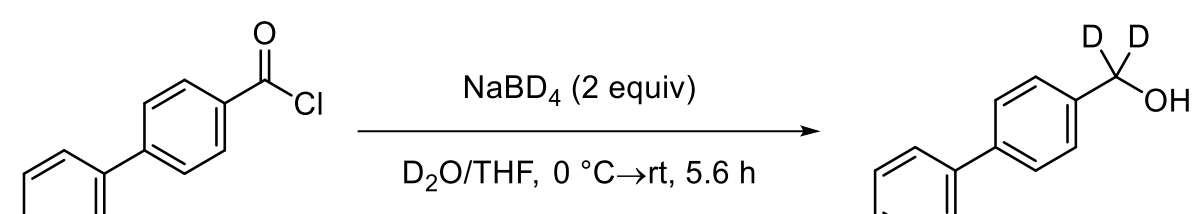

1a-*d*2

Biphenyl-4-methanol-*d*2 (1a-*d*2). Modified from previous report.[1] To a stirring solution of sodium borodeuteride (250.8 mg, 6 mmol, 2 equiv) in D_2O (3 mL) and THF (10 mL) at 0˚C (ice bath) was added 4-biphenylcarbonyl chloride (0.650 g, 3 mmol, 1 equiv) solution in 2 mL THF slowly over 10 min. The solution was brought to room temperature 6 minutes following addition and allowed to stir an additional 5.6 h. The organic layer was extracted with diethyl ether and washed with DI H_2O then brine. The solution was dried with Na_2SO_4 and solvent was evaporated *in vacuo* to yield the product as a powdery white solid (497.3 mg, 89%, 99% *D*) which was used without further purification. ***R**f* = 0.32 (25% EtOAc–hexanes); **m.p.**: 99.5–101.5 °C; **^{1}H NMR** ($CDCl_3$, 400 MHz): δ 7.61 (d, *J* = 8.0 Hz, 4H), 7.46 (t, *J* = 6.7 Hz, 4H), 7.36 (t, 7.4 Hz, 1H), 1.76 (s, 1H); **^{13}C{^{1}H} NMR** ($CDCl_3$, 100 MHz): δ 140.8, 140.6, 139.7, 128.7 (2C), 127.5 (2C), 127.3 (2C), 127.1 (2C), 64.4 (p, *J* = 21.7, 1C); **FTIR** (thin film): cm^{-1} ; 3219, 3058, 3032, 1484, 1400, 1237, 1087, 1061, 973, 952, 872, 744, 684, 596; **HRMS** (ESI$^+$, m/z): Calcd for $C_{13}H_9D_2$+ ($[M+H-H_2O]^+$): 169.0981; found: 169.0974.

Standard Condition A for Aldehyde Reduction for Alcohol Preparation

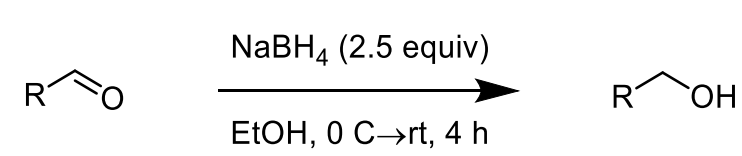

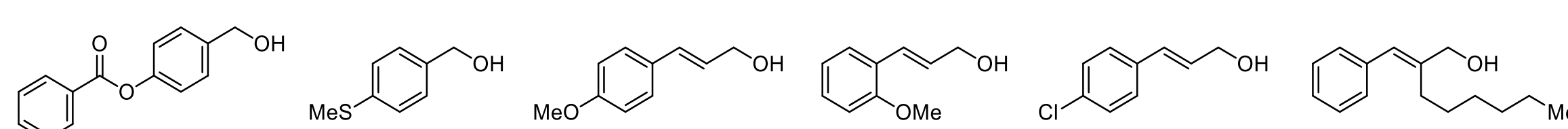

1g 1h 1k 1l 1m 1i

To a stirring solution of aldehyde (5.0 mmol, 1.0 equiv) in EtOH (25 mL) at 0 °C (ice bath) was added $NaBH_4$ (2.5 M in EtOH, 5 mL, 12.5 mmol, 2.5 equiv) over 5 min. The reaction was maintained at 0 °C for 5 min, then allowed to warm to room temperature for four hours or until total aldehyde consumption by monitor of TLC plate. The reaction was cooled to 0 °C (ice bath) and carefully quenched with a saturated solution of NH_4Cl (10 mL) added dropwise followed by 10 mL deionized water. The layers were separated and the aqueous layer was extracted with diethyl ether (10 mL x 3). The combined organic layers were washed with brine (10 mL x 2), dried with sodium sulfate, and concentrated *in vacuo* to yield the crude alcohol which was purified as noted.

4-(Benzoyloxy)benzenemethanol (1g). Synthesized using Standard Condition A on a 12.0 mmol scale starting from 4-(benzoyloxy)benzaldehyde. Isolated by flash column chromatography (silica gel, 100% hexanes to 40% ethyl acetate–hexanes) as a white powdery solid (1.04 g, 38%).[2] R_f = 0.22 (25% EtOAc–hexanes); **m.p.**: 72–74 °C; **^{1}H NMR** ($CDCl_3$, 400 MHz): δ 8.21 (d, *J* = 7.2 Hz, 2H), 7.65 (t, *J* = 7.4 Hz, 1H), 7.52 (t, *J* = 7.7 Hz, 2H), 7.44 (d, *J* = 8.4 Hz, 2H), 7.21 (d, *J* = 8.5 Hz, 2H), 4.72 (s, 2H), 1.77 (s, 1H); **^{13}C{^{1}H} NMR** ($CDCl_3$, 100 MHz): δ 165.3, 150.3, 138.5, 133.6, 130.2 (2C), 129.4, 128.6 (2C), 128.1 (2C), 121.8 (2C), 64.8; **FTIR** (thin film): cm^{-1} ; 3297, 3060, 2926, 2871, 1728, 1599, 1507, 1450, 1267, 1211, 1065, 985, 878, 697; **HRMS** (ESI^+, m/z): Calcd for $C_{14}H_{13}O_3^+$ ($[M+H]^+$): 229.0859; found: 229.0849.

4-(Methylthio)benzenemethanol (1h). Synthesized using Standard Condition A on a 3.0 mmol scale starting from 4-(methylthio)benzaldehyde. Isolated by flash column chromatography (silica gel, 100% hexanes to 20% ethyl acetate–hexanes) as a white solid (287.3 mg, 62%). R_f = 0.29 (25% EtOAc–hexanes); **m.p.**: 40–42 °C; **^{1}H NMR** ($CDCl_3$, 400 MHz): δ 7.28 (d, *J* = 8.4 Hz, 2H), 7.24 (d, *J* = 8.5 Hz, 2H), 4.63 (s, 2H), 2.47 (s, 3H), 1.69 (s, 1H); **^{13}C{^{1}H} NMR** ($CDCl_3$, 100 MHz): δ 137.8, 137.7, 127.6 (2C), 126.7 (2C), 64.9, 15.9; **FTIR** (thin film): cm^{-1} ; 3204, 2985, 2918, 2868, 2824, 1599, 1490, 1402, 1365, 1216, 1088, 1036, 797; **HRMS** (ESI^+, m/z): Calcd for $C_8H_9S^+$ ($[M+H-H_2O]^+$): 137.0419; found: 137.0414.

4-Methoxycinnamic alcohol (1k). Synthesized using Standard Condition A on a 2.0 mmol scale starting from 4-methoxycinnamaldehyde to afford **1g** as a white, crystalline solid (280.8 mg, 86%). The spectra matched previous report.[3] **^{1}H NMR** ($CDCl_3$, 400 MHz): δ 7.33 (d, *J* = 8.7 Hz, 2H), 6.86 (d, *J* = 8.7 Hz, 2H), 6.56 (d, *J* = 15.9 Hz, 1H), 6.24 (dt, *J* = 15.8, 6.0 Hz, 1H), 4.30 (dd, *J* = 6.0, 1.4 Hz, 2H), 3.81 (s, 3H), 1.49 (s, 1H); **^{13}C{^{1}H} NMR** ($CDCl_3$, 100 MHz): δ 159.2, 131.2, 129.1, 127.6 (2C), 126.1, 113.9 (2C), 63.9, 55.3.

2-Methoxycinnamyl alcohol (1l). Synthesized using Standard Condition A on a 5.0 mmol scale starting from 2-methoxycinnamaldehyde. Isolated as a colorless oil (244.0 mg, 30%) and used without further purification. The spectra matched previous report.[4]

4-Chlorocinnamyl alcohol (1m). Synthesized using Standard Condition A on a 5.0 mmol scale starting from 4-chlorocinnamaldehyde. Isolated as a white crystalline solid (436 mg, 52%) and used without further purification. The spectra matched previous report.[5]

α-Hexylcinnamic alcohol (1i). Synthesized using Standard Condition A on a 3.0 mmol scale from α-hexylcinnamaldehyde to afford **1i** as a colorless oil (519.0 mg, 79%). Spectra match previous report.[4] R_f = 0.56 (25% EtOAc–hexanes); **^{1}H NMR** ($CDCl_3$, 400 MHz): δ 7.34 (t, *J* = 7.4 Hz, 2H), 7.26-7.20 (m, 3H), 6.53 (s, 1H), 4.24 (d, *J* = 1.6 Hz, 2H), 2.29 (t, *J* = 8.1 Hz, 2H), 1.55 (br s, 1H), 1.52-1.46 (m, 2H), 1.34-1.24 (m, 6H), 0.87 (t, *J* = 6.8 Hz, 3H); **^{13}C{^{1}H} NMR** ($CDCl_3$, 100 MHz): δ 142.4, 137.5, 128.6 (2H), 128.2 (2H), 126.4, 125.2, 67.0, 31.6, 29.5, 28.7, 28.3, 22.6, 14.1; **FTIR** (thin film): cm^{-1} ; 3301, 2954, 2924, 2856, 1599, 1493, 1465, 1277, 1092, 1012, 9156, 747, 696; **HRMS** (ESI^+, m/z): Calcd for $C_{15}H_{21}^+$ ($[M+H-H_2O]^+$): 201.1638; found: 201.1627.

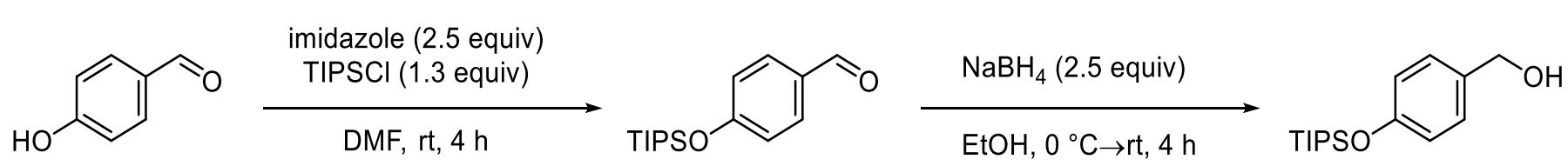

1e

4-(Triisopropylsilyloxy)benzenemethanol (1e).

<u>Step 1:</u> To a 10 mL round bottom flask was added 4-hydroxybenzaldehyde (0.61 g, 5 mmol, 1 equiv) and imidazole (0.85 g, 12.5 mmol, 2.5 equiv) followed by dimethylformaldehyde (2 mL). Triisopropylsilyl chloride (1.39 mL, 6.5 mmol, 1.3 equiv) was added slowly over 10 min. The reaction was allowed to stir at room temperature for 4 hours then was quenched with 5 mL DI H_2O. The organic layer was extracted with diethyl ether (3x 10 mL) and washed with brine (3x 10 mL). The solution was dried with sodium sulfate and solvent was evaporated *in vacuo*. The crude reaction mixture containing 4-(triisopropylsilyloxy)benzaldehyde was used directly in the next step without further purification.

<u>Step 2:</u> Synthesized using Standard Condition A on a 5 mmol scale starting from the crude 4-(triisopropylsiloxy)benzaldehyde above. Isolated by flash column chromatography (silica gel, 100% hexanes to 15% ethyl acetate–hexanes) as a colorless oil (996.8 mg, 69% over 2 steps). ***R_f*** = 0.49 (25% EtOAc–hexanes); **^{1}H NMR** ($CDCl_3$, 400 MHz): δ 7.22 (d, *J* = 8.4 Hz, 2H), 6.86 (d, *J* = 8.4 Hz, 2H), 4.60 (s, 2H), 1.60 (s, 1H), 1.26 (sept, *J* = 7.4 Hz, 3H), 1.10 (d, *J* = 7.4 Hz,18H); **$^{13}C\{^1H\}$ NMR** ($CDCl_3$, 100 MHz): δ 155.7, 133.3, 128.5 (2C), 119.9 (2C), 65.1, 17.9 (6C), 12.6 (3C); **FTIR** (thin film): cm^{-1} ; 3309, 2944, 2866, 1609, 1508, 1463, 1260, 996, 909, 881, 830, 733, 674; **HRMS** (ESI^+, m/z): Calcd for $C_{16}H_{27}OSi^+$ ($[M+H-H_2O]^+$): 263.1826; found: 263.1814.

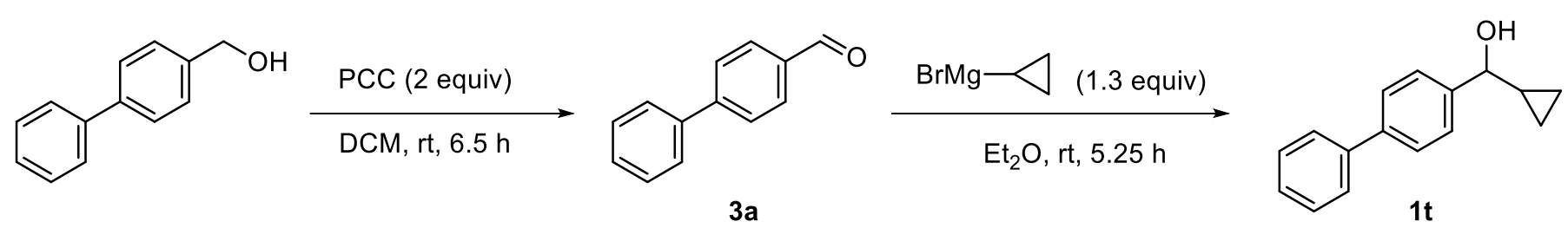

α-Cyclopropyl[1,1′-biphenyl]-4-methanol (1t).

<u>Step 1:</u> To a stirring solution of 4-biphenylmethanol (552.7 mg, 3 mmol, 1 equiv) in dichloromethane (15 mL) was added pyridinium chlorochromate (1.293 g, 6 mmol, 2 equiv). The reaction was stirred at room temperature for 6.5 h until completion of the reaction as monitored by a TLC plate. The reaction mixture was filtered through silica and eluted with dichloromethane, dried with sodium sulfate, and the solvent was evaporated *in vacuo*. The crude reaction mixture containing 4-biphenylcarboxaldehyde was used directly in the next step without further purification.

<u>Step 2:</u> To a flame-dried 3-neck round bottom flask, equipped with a Vigreux column fitted with an argon inlet, was added magnesium turnings ground with a mortar and pestle (0.51 g, 21 mmol, 7 equiv) and catalytic iodine. Iodine sublimation was initiated using a heat gun. After 30 min, diethyl ether (12 mL) was added followed by dropwise addition of bromocyclopropane (0.312 mL, 3.9 mmol, 1.3 equiv). After 25 minutes the reaction had warmed and turned from yellow to a milky white and was allowed to stir for an additional 5 minutes. The crude 4-biphenylcarboxaldehyde (3 mmol) in 30 mL diethyl ether was added dropwise and stirred at room temperature for 5.25 h. The reaction was carefully quenched with 20 mL 1.0 M HCl to minimize rigorous foaming. The solution was filtered with cotton and the organic layer was extracted with diethyl ether (2x 20 mL) and washed with brine (2x 20 mL). The organic layer was dried with sodium sulfate and evaporated *in vacuo* to yield α-Cyclopropyl[1,1′-biphenyl]-4-methanol which was isolated by flash column chromatography (silica gel, 100% hexanes to 20% ethyl acetate–hexanes) as a yellow solid (278.0 mg, 41% over 2 steps). ***R_f*** = 0.40 (25% EtOAc–hexanes); **m.p.**: 69.5–70.5 °C; **^{1}H NMR** ($CDCl_3$, 400 MHz): δ 7.60 (d, *J* = 8.3 Hz, 4H), 7.51 (d, *J* = 8.2 Hz, 2H), 7.45 (d, *J* = 8.2 Hz, 2H), 7.35 (t, *J* = 7.4 Hz, 1H), 4.06 (d, *J* = 7.4 Hz, 1H), 1.99 (s, 1H), 1.27 (qt, *J* = 8.2, 4.9 Hz, 1H), 0.68 (tdd, *J* = 8.4, 5.1, 3.9 Hz, 1H), 0.60 (tt, *J* = 8.5, 4.4 Hz, 1H), 0.51 (dq, *J* = 9.4, 4.7 Hz, 1H), 0.42 (dq, *J* = 9.5, 4.6 Hz); **$^{13}C\{^1H\}$ NMR** ($CDCl_3$, 100 MHz): δ 142.8, 140.9, 140.4, 128.7 (2C), 127.2, 127.1 (2C), 127.1 (2C), 126.4 (2C), 78.4, 19.2, 3.7, 2.9; **FTIR** (thin film): cm^{-1} ; 3320, 3079, 3030, 3003, 2871, 1487, 1404, 1242, 1026, 1005, 826, 757, 690; **HRMS** (ESI^+, m/z): Calcd for $C_{16}H_{15}^+$ ($[M+H-H_2O]^+$): 207.1168; found: 207.1158.

Standard Conditions for TEMPO Oxoammonium Ion Preparation

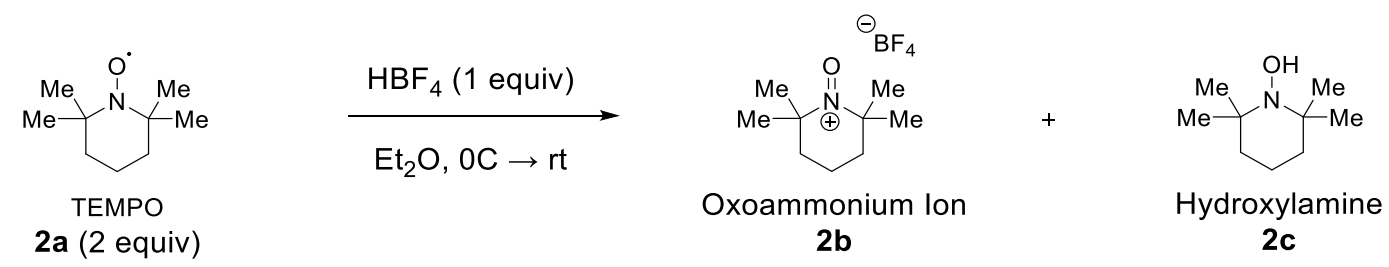

2,2,6,6-tetramethyl-1-oxopiperidinium (2b) and 1-hydroxy-2,2,6,6-tetramethylpiperidine (2c). To a 50 mL round bottomed flask was added 2,2,6,6-tetramethyl-1-pipiridinyloxyl **2a** (1.56 g, 10 mmol, 1 equiv) and diethyl ether (30 mL). The solution was stirred until **2a** dissolved and cooled to 0°C in an ice bath. HBF_4

(2.88 mL, 22 mmol, 2.2 equiv, 48% in H_2O) was added dropwise over 10 minutes and allowed to stir for an additional 5 minutes. The solution changed colors from red to yellow and two layers formed. The solution was allowed to stir at room temperature for 90 mins or until no red color remained. The solution was filtered using a Büchner funnel and rinsed with diethyl ether. The precipitate yielded product **2b** as a bright yellow solid (909.4 mg, 37%) and was used without further purification.

4. Alcohol Oxidation Protocols and Characterization of Novel Compounds

Standard Condition B for Solid Alcohols

To a 2-dram vial equipped with a Teflon-coated stir bar was added TEMPO **2a** (93.8 mg, 0.6 mmol, 2 equiv) and the alcohol (0.3 mmol, 1 equiv). The vial was capped with a rubber septum and sealed with parafilm. Using a needle inserted through the rubber septum, air was evacuated and backfilled with argon three times. Under argon using a 1 mL syringe, dry MeCN (2.75 mL) and a stock solution of 4-CzIPN (0.96 mM in MeCN, 0.25 mL, 0.00024 mmol, 0.0008 equiv) were added, followed by glacial acetic acid (67.0 µL, 69.7 mg, 1.16 mmol, 3.9 equiv). The vial was stirred at 800 rpm for 19 h under irradiation of 400 nm light (2x 100 W LED panels, 1” from vial). Room temperature was maintained using a circulating fan. The resulting crude mixture was quenched by removal of 400 nm light source and exposure to air, then concentrated *in vacuo* to yield the crude product.

Standard Condition B for Liquid Alcohols

To a 2-dram vial equipped with a Teflon-coated stir bar was added TEMPO **2a** (93.8 mg, 0.6 mmol, 2 equiv). The vial was capped with a rubber septum and sealed with parafilm. Using a needle inserted through the rubber septum, air was evacuated and backfilled with argon three times. Under argon using a 1 mL syringe, dry MeCN (2.75 mL) and a stock solution of 4-CzIPN (0.96 mM in MeCN, 0.25 mL, 0.00024 mmol, 0.0008 equiv) were added, followed by glacial acetic acid (67.0 µL, 69.7 mg, 1.16 mmol, 3.9 equiv) and alcohol (0.3 mmol, 1 equiv). The vial was stirred at 800 rpm for 19 h under irradiation of 400 nm light (2x 100W, 400 nm LED panels, 1” from vial). Room temperature was maintained using a circulating fan. The resulting crude mixture was quenched by removal of 400 nm light source and exposure to air and concentrated *in vacuo* to yield the crude product.

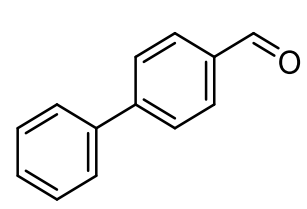

3a

4-Biphenylcarboxaldehyde (3a). Synthesized using Standard Condition B. Isolated by flash column chromatography (silica gel, 100% hexanes to 5% ethyl acetate–hexanes) as a white solid (45.2 mg, 83%). The spectra match previous report.[6] The ^{1}H and ^{13}C NMR are included as evidence of purity. **^{1}H NMR** ($CDCl_3$, 400 MHz): δ 10.06 (s, 1H), 7.96 (d, *J* = 8.5 Hz, 2H), 7.76 (d, *J* = 8.4 Hz, 2H), 7.64 (d, *J* = 6.9 Hz, 2H), 7.49 (t, *J* = 7.3 Hz, 2H), 7.41 (t, 7.3 Hz, 1H); **^{13}C{^{1}H} NMR** ($CDCl_3$, 100 MHz): δ 191.9, 147.1, 139.6, 135.1, 130.2 (2C), 128.9 (2C), 128.4, 127.6 (2C), 127.3 (2C).

1.0 mmol scale:

To a 25-mL schlenk flask equipped with a Teflon-coated stir bar was added TEMPO **2a** (156.3 mg, 2.0 mmol, 2 equiv) and the biphenyl-4-methanol (184.2 mg, 1.0 mmol, 1 equiv). The flask was capped with a rubber septum and sealed with parafilm. Using the sidearm stopcock joint, air was evacuated and backfilled with argon three times. Under argon using a 10 mL syringe, dry MeCN (9.2 mL) and a stock solution of 4-CzIPN (0.96 mM in MeCN, 0.83 mL, 0.0008 mmol, 0.0008 equiv) were added, followed by glacial acetic

acid (0.23 mL, 240.2 mg, 4.0 mmol, 4.0 equiv). The vial was stirred at 800 rpm for 19 h under irradiation of 400 nm light (2x 100 W LED panels, 1” from vial). Room temperature was maintained using a circulating fan. The resulting crude mixture was quenched by removal of 400 nm light source and exposure to air, then concentrated *in vacuo* to yield the crude product. Isolation by flash column chromatography (silica gel, 100% hexanes to 5% ethyl acetate–hexanes) provided **3a** as a clear oil (163.3 mg, 90%). (Before, during, and after photos provided below with the consumption of TEMPO evident in the color change from red/orange to yellow.)

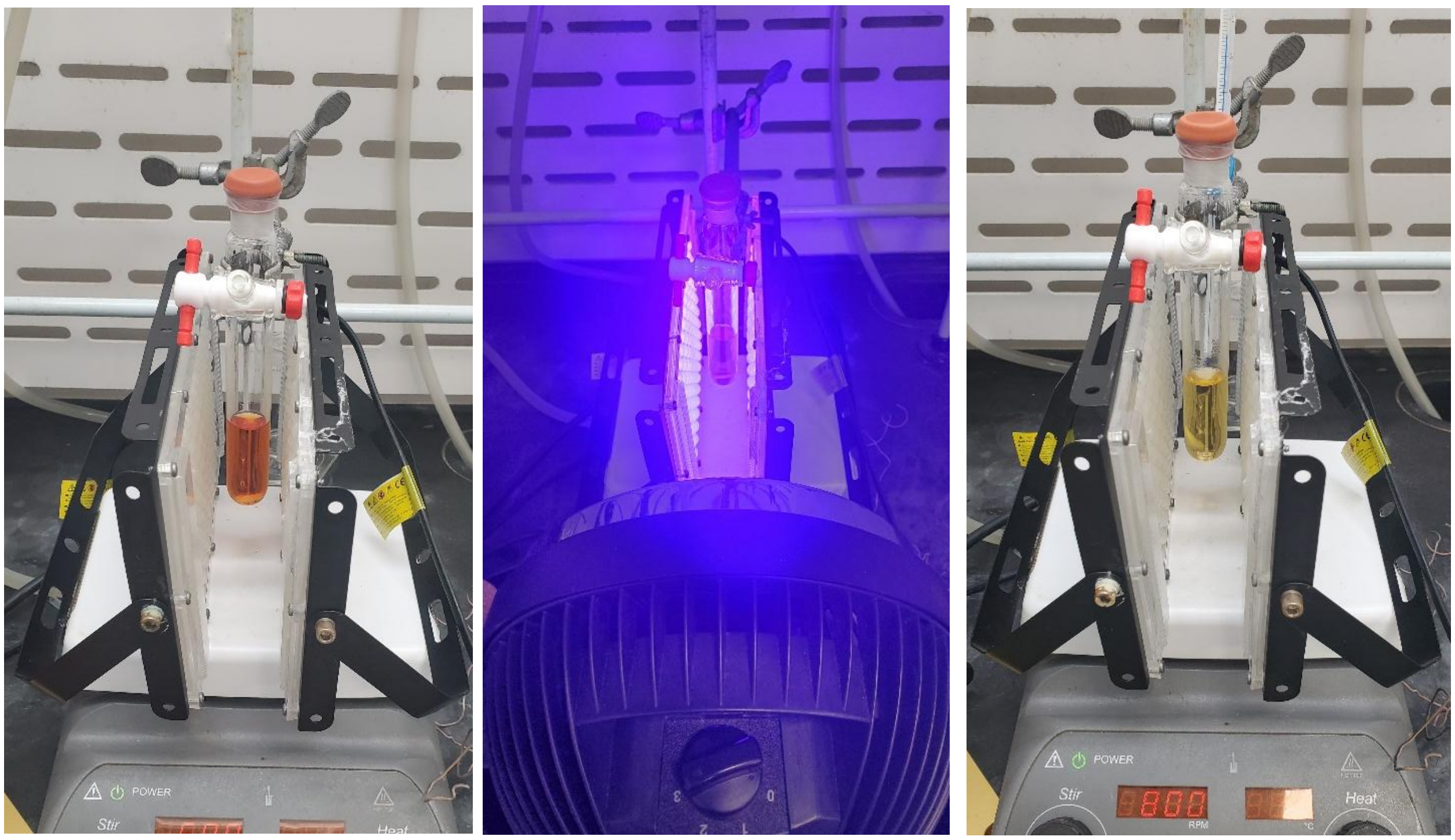

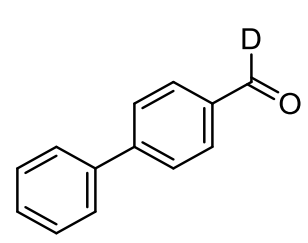

3a-*d*

4-Biphenylcarboxaldehyde-*d* (3a-*d*). Synthesized using Standard Condition B. Isolated by flash column chromatography (silica gel, 100% hexanes to 4% ethyl acetate–hexanes) as a white solid (39.2 mg, 71%, 99% *D*). ***R$_f$*** = 0.65 (25% EtOAc–hexanes); **m.p.**: 58–59 °C; **^{1}H NMR** ($CDCl_3$, 400 MHz): δ 7.96 (d, *J* = 8.2 Hz, 2H), 7.76 (d, *J* = 8.2 Hz, 2H), 7.64 (d, *J* = 7.2 Hz, 2H), 7.49 (t, *J* = 7.4 Hz, 2H), 7.43 (t, *J* = 7.3 Hz, 1H); **^{13}C{^{1}H} NMR** ($CDCl_3$, 100 MHz): δ 191.6 (t, *J* = 26.5 Hz, 1C), 147.1, 139.6, 135.0 (t, *J* = 3.7 Hz, 1C), 130.2 (2C), 129.0 (2C), 128.4, 127.6 (2C), 127.3 (2C); **FTIR** (thin film): cm^{-1} ; 3028.04, 2111, 2069, 1670, 1600, 1230, 818, 758, 700; **HRMS** (ESI$^+$, m/z): Calcd for $C_{13}H_{10}DO^+$ ($[M+H]^+$): 184.0867; found: 184.0859.

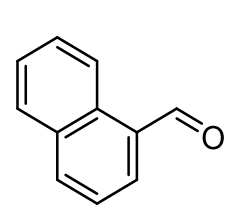

3b

1-Naphthaldehyde (3b). Synthesized using Standard Condition B. Isolated by flash column chromatography (silica gel, 100% hexanes to 5% ethyl acetate–hexanes) as a clear oil (37.1 mg, 79%). ***R$_f$*** = 0.65 (25% EtOAc–hexanes); **^{1}H NMR** ($CDCl_3$, 400 MHz): δ 10.39 (s, 1H), 9.26 (d, *J* = 8.8 Hz, 1H), 8.09 (d, *J* = 8.4 Hz, 1H), 7.98 (dd, *J* = 7.1, 1.3 Hz, 1H), 7.92 (d, *J* = 8.4 Hz, 1H), 7.69 (ddd, *J* = 8.5, 6.9, 1.4 Hz, 1H), 7.64-7.57 (m, 2H); **^{13}C{^{1}H} NMR** ($CDCl_3$, 100 MHz): δ 193.6, 136.7, 135.3, 133.6, 131.3, 130.4, 129.0,

128.4, 126.9, 124.8 (2C); **FTIR** (thin film): cm^{-1} ; 3054, 2725, 2104, 2065, 1676, 1602, 1509, 1216, 1169, 1055, 885, 802, 756, 695, 648; **HRMS** (ESI$^+$, m/z): Calcd for $C_{11}H_9O^+$ ([M+H]$^+$): 157.0648; found: 157.0656.

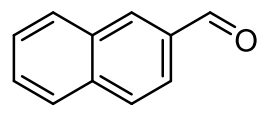

3c

2-Naphthaldehyde (3c). Synthesized using Standard Condition B. Isolated by flash column chromatography (silica gel, 100% hexanes to 5% ethyl acetate–hexanes) as a white solid (22.9 mg, 49%). R_f = 0.65 (25% EtOAc–hexanes); **m.p.**: 61.5–62.5 °C; **^{1}H NMR** ($CDCl_3$, 400 MHz): δ 10.15 (s, 1H), 8.32 (s, 1H), 8.00-7.88 (m, 4H), 7.66-7.56 (m, 2H); **^{13}C{^{1}H} NMR** ($CDCl_3$, 100 MHz): δ 192.3, 136.4, 134.6, 134.1, 132.5, 129.5, 129.1, 129.0, 128.0, 127.0, 122.6; **FTIR** (thin film): cm^{-1} ; 3363, 3065, 2831, 1686, 1625, 1459, 1364, 1256, 1164, 1008, 871, 834, 748; **HRMS** (ESI$^+$, m/z): Calcd for $C_{11}H_9O^+$ ([M+H]$^+$): 157.0648; found: 157.0643.

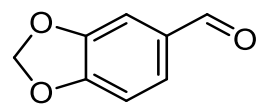

3d

Piperonal (3d). Synthesized using Standard Condition B. Isolated by flash column chromatography (silica gel, 100% hexanes to 5% ethyl acetate–hexanes) as a white solid (40.0 mg, 89%). The spectra match previous report.[7] The ^{1}H and ^{13}C NMR are included as evidence of purity. **^{1}H NMR** ($CDCl_3$, 400 MHz): δ 9.79 (s, 1H), 7.39 (d, J = 7.9 Hz, 1H), 7.31 (s, 1H), 6.91 (dd, J = 7.9, 1.2 Hz, 1H), 6.06 (s, 2H); **^{13}C{^{1}H} NMR** ($CDCl_3$, 100 MHz): δ 190.3, 153.0, 148.6, 131.7, 128.7, 108.3, 106.7, 102.0.

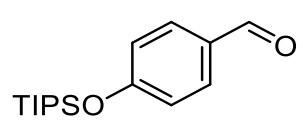

3e

4-((Triisopropylsilyl)oxy)benzaldehyde (3e). Synthesized using Standard Condition B. Isolated by flash column chromatography (silica gel, 100% hexanes to 6% ethyl acetate–hexanes) as a colorless oil (60.5 mg, 72%). R_f = 0.79 (25% EtOAc–hexanes); **^{1}H NMR** ($CDCl_3$, 400 MHz): δ 9.87 (s, 1H), 7.78 (d, J = 8.4 Hz, 2H), 6.97 (d, J = 8.4 Hz, 2H), 1.28 (sept, J = 7.4 Hz, 3H), 1.10 (d, J = 7.5 Hz, 18H); **^{13}C{^{1}H} NMR** ($CDCl_3$, 100 MHz): δ 190.8, 161.8, 131.9 (2C), 130.0, 120.2 (2C), 17.7 (6C), 12.6 (3C); **FTIR** (thin film): cm^{-1} ; 2944, 2867, 2729, 1698, 1595, 1506, 1463, 1272, 1209, 1153, 996, 903, 837, 682; **HRMS** (ESI$^+$, m/z): Calcd for $C_{16}H_{27}O_2Si+$ ([M+H]$^+$): 279.1775; found: 279.1766.

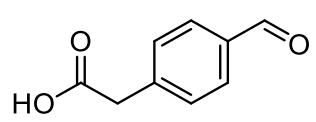

3f

4'-Formylphenylacetic acid (3f). Synthesized using Standard Condition B and isolated via acid-base extraction using the following procedure. 15 mL ethyl acetate was added to the crude reaction mixture, and the product was extracted into the aqueous layer using sodium hydroxide (10 mL 3x). The solution was neutralized with 30 mL 1.0 M HCl and the product was extracted into the organic layer with ethyl acetate (10 mL 3x). The organic layer was dried using sodium sulfate followed by rotary evaporation to afford the product as a white solid (35.9 mg, 73%). R_f = 0.11 (25% EtOAc–hexanes); **m.p.**: 147.5–149 °C; **^{1}H NMR** ($CDCl_3$, 400 MHz): δ 9.99 (s, 1H), 7.86 (d, J = 8.0 Hz, 2H), 7.46 (d, J = 8.0 Hz, 2H), 3.75 (s, 2H); **^{13}C{^{1}H} NMR** ($CDCl_3$, 100 MHz): δ 192.0, 176.7, 140.0, 135.4, 130.1 (2C), 130.0 (2C), 41.0; **FTIR** (thin film): cm^{-1} ; 2922, 2770, 2625, 1717, 1656, 1575, 1394, 1254, 1135, 900, 800, 698, 629; **HRMS** (ESI$^-$, m/z): Calcd for $C_8H_7O^-$ ([M–H–CO_2]$^-$): 119.0502; found: 119.0510.

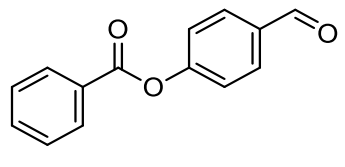

3g

4-(Benzoyloxy)-benzaldehyde (3g). Synthesized using Standard Condition B. Isolated by flash column chromatography (silica gel, 100% hexanes to 15% ethyl acetate–hexanes) as a white solid (59.1 mg, 87%). R_f = 0.60 (25% EtOAc–hexanes); **m.p.**: 89.5–91.5 °C; **^{1}H NMR** ($CDCl_3$, 400 MHz): δ 10.01 (s, 1H), 8.20 (d, J = 7.3 Hz, 2H), 7.96 (d, J = 8.6 Hz, 2H), 7.66 (t, J = 7.4 Hz, 1H), 7.53 (t, J = 7.7 Hz, 2H), 7.41 (d, J = 8.5

Hz, 2H); **^{13}C{^{1}H} NMR** (CDCl$_3$, 100 MHz): δ 190.9, 164.4, 155.5, 133.9, 133.9, 131.2 (2C), 130.1 (2C), 128.7, 128.6 (2C), 122.4 (2C); **FTIR** (thin film): cm^{-1} ; 2918, 2849, 2821, 2726, 1735, 1696, 1595, 1503, 1451, 1252, 1205, 1156, 1058, 704; **HRMS** (ESI$^+$, m/z): Calcd for $C_{14}H_{11}O_3^+$ ([M+H]$^+$): 227.0703; found: 227.0694.

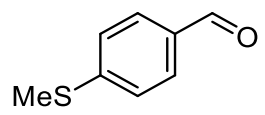

3h

4-(Methylthio)benzaldehyde (3h). Synthesized using Standard Condition B. Isolated by flash column chromatography (silica gel, 100% hexanes to 5% ethyl acetate–hexanes) as an orange oil (28.2 mg, 62%). ***R$_f$*** = 0.60 (25% EtOAc–hexanes); **^{1}H NMR** (CDCl$_3$, 400 MHz): δ 9.94 (s, 1H), 7.79 (d, *J* = 8.1 Hz, 2H), 7.34 (d, *J* = 8.1 Hz, 2H), 2.55 (s, 3H); **^{13}C{^{1}H} NMR** (CDCl$_3$, 100 MHz): δ 190.9, 147.5, 132.4, 129.6 (2C), 124.7 (2C), 14.3; **FTIR** (thin film): cm^{-1} ; 2973, 2921, 2828, 2735, 1690, 1588, 1559, 1214, 1169, 1090, 835, 808, 690; **HRMS** (ESI$^+$, m/z): Calcd for $C_8H_9OS^+$ ([M+H]$^+$): 153.0369; found: 153.0361.

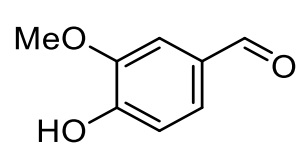

3i

Vanillin (3i). Synthesized using Standard Condition B. Isolated by flash column chromatography (silica gel, 100% hexanes to 15% ethyl acetate–hexanes) as a white solid (12.4 mg, 27%). The spectra match the commercial compound. The ^{1}H and ^{13}C NMR are included as evidence of purity. **^{1}H NMR** (CDCl$_3$, 400 MHz): δ 9.82 (s, 1H), 7.43-7.41 (m, 2H), 7.04 (d, *J* = 8.6 Hz, 1H), 6.30 (s, 1H), 3.96 (s, 3H); **^{13}C{^{1}H} NMR** (CDCl$_3$, 100 MHz): δ 191.0, 151.7, 147.1, 129.7, 127.6, 114.3, 108.7, 56.0.

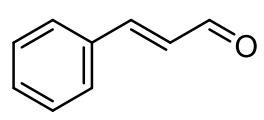

3j

***E*-Cinnamaldehyde (3j).** Synthesized using Standard Condition B. An *E:Z* ratio of 14:1 was determined by ^{1}H NMR of the crude reaction. Isolated by flash column chromatography (silica gel, 100% hexanes to 5% ethyl acetate–hexanes) as an amber oil (30.9 mg, 78%) of only the *E* isomer. Isolated. The spectra match a previous report.[8] The ^{1}H and ^{13}C NMR are included as evidence of purity. **^{1}H NMR** (CDCl$_3$, 400 MHz): δ 9.74 (d *J* = 7.7 Hz, 1H), 7.60 (dd, *J* = 6.6, 2.9 Hz, 2H), 7.51 (d, *J* = 15.9 Hz, 1H), 7.49-7.43 (m, 3H), 6.75 (dd, *J* = 15.9, 7.7 Hz, 1H); **^{13}C{^{1}H} NMR** (CDCl$_3$, 100 MHz): δ 193.3, 152.4, 133.4, 130.8, 128.6 (2C), 128.0 (2C), 128.0.

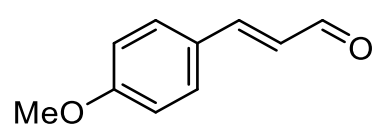

3k

4'-Methoxycinnamaldehyde (3k). Synthesized using Standard Condition B. An *E:Z* ratio >20:1 was determined by ^{1}H NMR of the crude reaction mixture. Isolated by flash column chromatography (silica gel, 100% hexanes to 9% ethyl acetate–hexanes) as a white solid (33.5 mg, 69%). The spectra match the commercial compound. The ^{1}H and ^{13}C NMR are included as evidence of purity. **^{1}H NMR** (CDCl$_3$, 400 MHz): δ 9.65 (d, *J* = 7.8 Hz, 1H), 7.52 (d, *J* = 8.8 Hz, 2H), 7.42 (d, *J* = 15.8 Hz, 1H), 6.94 (d, *J* = 8.8 Hz, 2H), 6.61 (dd, *J* = 15.8, 7.8 Hz, 1H), 3.86 (s, 3H); **^{13}C{^{1}H} NMR** (CDCl$_3$, 100 MHz): δ 193.7, 162.0, 152.7, 130.2 (2C), 126.5, 126.3, 114.4 (2C), 55.3.

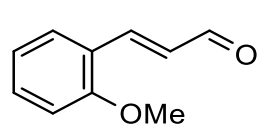

3l

2-Methoxycinnamaldehyde (3l). Synthesized using Standard Condition B. An *E:Z* ratio of 6.3:1 was determined by ^{1}H NMR of the crude reaction. Isolated by flash column chromatography (silica gel, 100% hexanes to 4% ethyl acetate–hexanes) as a white solid (33.4 mg, 69%) of only the *E* isomer. The spectra match a previous report.[9] The ^{1}H and ^{13}C NMR are included as evidence of purity. **^{1}H NMR** (CDCl$_3$, 400 MHz): δ 9.68 (d, *J* = 7.9 Hz, 1H), 7.84 (d, *J* = 16.0 Hz, 1H), 7.54 (dd, *J* = 7.7, 1.7 Hz, 1H), 7.41 (td, *J* = 8.1,

1.6 Hz, 1H), 6.99 (t, *J* = 7.6 Hz, 1H), 6.94 (d, *J* = 8.4 Hz, 1H), 6.78 (dd, *J* = 16.4, 7.9 Hz, 1H), 3.90 (s, 3H); **^{13}C{^{1}H} NMR** ($CDCl_3$, 100 MHz): δ 194.6, 158.2, 148.2, 132.6, 128.9, 128.8, 122.8, 120.8, 111.2, 55.3.

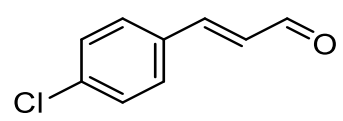
3m

4-Chlorocinnamaldehyde (3m). Synthesized using Standard Condition B. An *E:Z* ratio of 12:1 was determined by ^{1}H NMR of the crude reaction. Isolated by flash column chromatography (silica gel, 100% hexanes to 4% ethyl acetate–hexanes) as a white crystalline solid (42.8 mg, 86%) of only the *E* isomer. The spectra match the commercial compound. The ^{1}H and ^{13}C NMR are included as evidence of purity. **^{1}H NMR** ($CDCl_3$, 400 MHz): δ 9.69 (d, *J* = 7.6 Hz, 1H), 7.49 (d, *J* = 8.5 Hz, 2H), 7.43 (d, *J* = 15.7 Hz, 1H), 7.40 (d, *J* = 8.50 Hz, 2H), 6.68 (dd, *J* = 16.0, 7.7 Hz, 1H); **^{13}C{^{1}H} NMR** ($CDCl_3$, 100 MHz): δ 193.9, 151.1, 137.2, 132.4, 129.6 (2C), 129.4 (2C), 128.8.

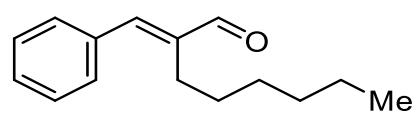
3n

α-Hexylcinnamaldehyde (3n). Synthesized using Standard Condition B. An *E:Z* ratio of >20:1 was determined by ^{1}H NMR of the crude reaction. Isolated by flash column chromatography (silica gel, 100% hexanes to 4% ethyl acetate–hexanes) as a colorless oil (38.6 mg, 59%) of only the *E* isomer. ***R_f*** = 0.80 (25% EtOAc–hexanes); **^{1}H NMR** ($CDCl_3$, 400 MHz): δ 9.55 (s, 1H), 7.49 (d, *J* = 7.5 Hz, 2H), 7.45 (t, *J* = 7.4 Hz, 2H), 7.40 (t, *J* = 7.0 Hz, 1H), 7.21 (s, 1H), 2.52 (t, *J* = 8.0 Hz, 2H), 1.51-1.45 (m, 2H), 1.40-1.36 (m, 2H), 1.31-1.29 (m, 4H), 0.88 (t, *J* = 6.7 Hz, 3H); **^{13}C{^{1}H} NMR** ($CDCl_3$, 100 MHz): δ 195.7, 149.8, 143.3, 134.9, 129.6 (2C), 129.5, 128.7 (2C), 31.5, 29.5, 28.2, 24.7, 22.6, 14.0; **FTIR** (thin film): cm^{-1} ; 2954, 2926, 2856, 2711, 1678, 1623, 1456, 1200, 1090, 754, 695; **HRMS** (ESI^+, m/z): Calcd for $C_{15}H_{21}O^+$ ($[M+H]^+$): 217.1587; found: 217.1597.

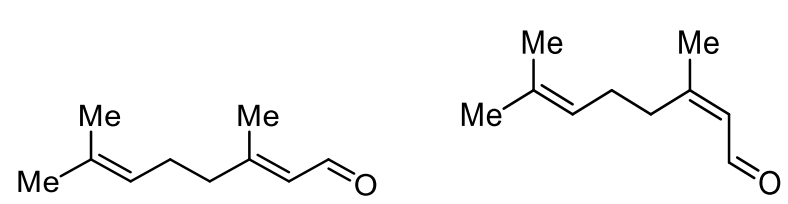
3o + 3o'

Geranial (3o) and Neral (3o'). Synthesized using Standard Condition B. Isolated by flash column chromatography (silica gel, 100% hexanes to 5% ethyl acetate–hexanes) as a sweet-smelling pale yellow oil and an inseparable 1:1.1 mixture of **3o:3o'** (39.7 mg, 87%). The spectra matched previous reports.[10] The ^{1}H and ^{13}C NMR are included as evidence of purity. **^{1}H NMR** ($CDCl_3$, 400 MHz): δ 9.97 (d, *J* = 8.2 Hz, 1H, *3o*), 9.86 (d, *J* = 8.3 Hz, 1H, *3o'*), 5.85 (d, *J* = 8.2 Hz, 1H *3o* + 1H *3o'*), 5.07-5.02 (m, 1H *3o* + 1H *3o'*), 2.57 (t, *J* = 7.4 Hz, 2H, *3o'*), 2.24-2.15 (m, 4H *3o* + 2H *3o'*), 2.16 (s, 1H, *3o*), 1.97 (s, 1H, *3o'*), 1.67 (s, 3H *3o* + 3H *3o'*), 1.59 (s, 3H, *3o*), 1.58 (s, 3H, *3o'*); **^{13}C{^{1}H} NMR** ($CDCl_3$, 100 MHz): δ 191.2 (*3o*), 190.6 (*3o'*), 163.8 (*3o*), 163.7 (*3o'*), 133.5 (*3o'*), 132.7 (*3o*), 128.4 (*3o'*), 127.1 (*3o*), 122.3 (*3o'*), 121.9 (*3o*), 40.3 (*3o*), 32.3 (*3o'*), 26.7 (*3o'*), 25.4 (2C *3o* + 1C *3o'*), 24.9 (*3o'*), 17.5 (1C *3o* + 1C *3o'*), 17.3 (*3o*).

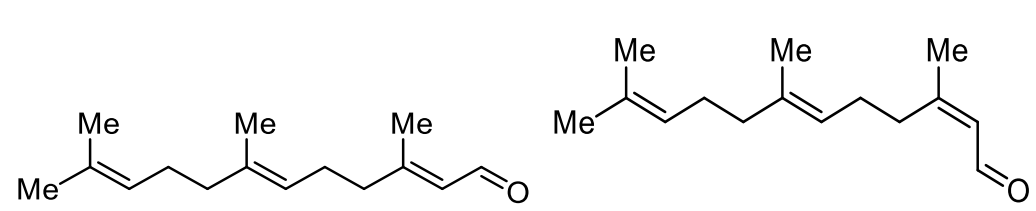
3p + 3p'

(2*E*,6*E*)-Farnesal (3p) and (2*Z*,6*E*)-Farnesal (3p'). Synthesized using Standard Condition B. An *E:Z* ratio of 1:1 was determined by ^{1}H NMR of the crude reaction. Isolated by flash column chromatography (silica gel, 100% hexanes to 5% ethyl acetate–hexanes) as a colorless oil (56.6 mg, 86%) and 1:1.5 ratio of **3p**:**3p'**.

(2*E*,6*E*) isomer (3p) with trace 3p' (>20:1). Isolated as a colorless oil (15.0 mg). ***R_f*** = 0.79 (25% EtOAc–hexanes); **^{1}H NMR** ($CDCl_3$, 400 MHz): δ 9.99 (d, *J* = 8.1 Hz, 1H), 5.89 (dd, *J* = 8.2, 1.3 Hz, 1H), 5.11–5.03 (m, 2H), 2.27-2.19 (m, 4H), 2.17 (d, *J* = 1.3 Hz, 3H), 2.09-2.02 (m, 2H), 2.00-1.94 (m, 2H), 1.67 (s, 3H), 1.60 (s, 3H), 1.59 (s, 3H); **^{13}C{^{1}H} NMR (3d')** ($CDCl_3$, 100 MHz): δ 191.3, 164.0, 136.4, 131.4, 127.3, 124.0, 122.3, 40.5, 39.5, 26.5, 25.6, 25.5, 17.6, 17.5, 15.9; **FTIR** (thin film): cm^{-1} ; 2966, 2916, 2853, 1672, 1441, 1377, 1193, 1119, 830; **HRMS** (ESI^+, m/z): Calcd for $C_{15}H_{25}O^+$ ($[M+H]^+$): 221.1900; found: 221.1911.

(2*Z*,6*E*) isomer (3p') with some 3p isomer (4.2:1). Isolated as a colorless oil (41.6 mg). ***R*f** = 0.90 (25% EtOAc–hexanes); **^{1}H NMR** ($CDCl_3$, 400 MHz): δ 9.90 (d, *J* = 8.3 Hz, 1H), 5.87 (d, *J* = 8.2 Hz, 1H), 5.12-5.03 (m, 2H), 2.58 (t, *J* = 7.5 Hz, 1H), 2.27-2.22 (m, 2H), 2.07-2.01 (m, 2H), 1.98 (s, 5H), 1.66 (s, 4H), 1.58 (s, 6H); **^{13}C{^{1}H} NMR** ($CDCl_3$, 100 MHz): δ 190.8, 164.0, 137.2, 131.5, 128.5, 123.9, 122.0, 39.5, 32.5, 26.9, 26.4, 25.6, 25.0, 17.6, 16.0; **FTIR** (thin film): cm^{-1} ; 2966, 2915, 2855, 1673, 1441, 1376, 1152, 842; **HRMS** (ESI$^+$, m/z): Calcd for $C_{15}H_{25}O^+$ ([M+H]$^+$): 221.1900; found: 221.1893.

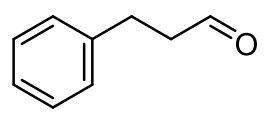

3q

3-Phenylpropanal (3q). Synthesized using Standard Condition B. Isolated by flash column chromatography (silica gel, 100% hexanes to 5% ethyl acetate–hexanes) as a colorless liquid (34.8 mg, 86%). The spectra match previous report.[11] The ^{1}H and ^{13}C NMR are included as evidence of purity. **^{1}H NMR** ($CDCl_3$, 400 MHz): δ 9.83 (s, 1H), 7.31 (t, *J* = 7.7 Hz, 2H), 7.25-7.18 (m, 3H), 2.97 (t, *J* = 7.6 Hz, 2H), 2.79 (t, *J* = 7.5 Hz, 2H); **^{13}C{^{1}H} NMR** ($CDCl_3$, 100 MHz): δ 201.6, 140.2, 128.5 (2C), 128.2 (2C), 126.2, 45.2, 28.0.

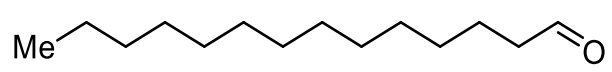

3r

Tetradecanal (3r). Synthesized using Standard Condition B. Isolated by flash column chromatography (silica gel, 100% hexanes to 8% ethyl acetate–hexanes) as a white solid (41.0 mg, 64%). The spectra matched a previous report.[12] The ^{1}H and ^{13}C NMR are included as evidence of purity. **^{1}H NMR** ($CDCl_3$, 400 MHz): δ 9.75 (t, *J* = 1.9 Hz, 1H), 2.41 (td, *J* = 7.3, 1.9 Hz, 2H), 1.61 (pent, *J* = 7.3 Hz, 2H), 1.32-1.21 (m, 20H), 0.87 (t, *J* = 6.8 Hz, 3H); **^{13}C{^{1}H} NMR** ($CDCl_3$, 100 MHz): δ 202.9, 43.7, 31.8, 29.6-29.0 (9C), 22.6, 22.0, 14.1

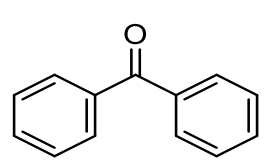

3s

Benzophenone (3s). Synthesized using Standard Condition B. Isolated by flash column chromatography (silica gel, 100% hexanes to 8% ethyl acetate–hexanes) as a white solid (25.0 mg, 46%). The spectra match previous report.[13] The ^{1}H and ^{13}C NMR are included as evidence of purity. **^{1}H NMR** ($CDCl_3$, 400 MHz): δ 7.8 (d, *J* = 7.8 Hz, 4H), 7.59 (tt, *J* = 7.4, 1.4 Hz, 2H), 7.49 (t, *J* = 7.6 Hz, 4H); **^{13}C{^{1}H} NMR** ($CDCl_3$, 100 MHz): δ 196.7, 137.4 (2C), 132.3 (2C), 129.9 (4C), 128.2 (4C).

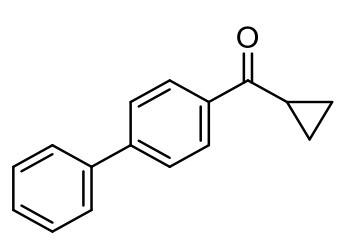

3t

[1,1′-Biphenyl]-4-ylcyclopropylmethanone (3t). Synthesized using Standard Condition B. Isolated by flash column chromatography (silica gel, 100% hexanes to 4% ethyl acetate–hexanes) as a white solid (14.8 mg, 22%). No ring-opened product was observed. ***R*f** = 0.70 (25% EtOAc–hexanes); **m.p.**: 103–105 °C; **^{1}H NMR** ($CDCl_3$, 400 MHz): δ 8.10 (d, *J* = 8.3 Hz, 2H), 7.71 (d, *J* = 8.4 Hz, 2H), 7.65 (d, *J* = 7.1 Hz, 2H), 7.48 (t, *J* = 7.4 Hz, 2H), 7.41 (t, *J* = 7.3 Hz, 1H), 2.73 (tt, *J* = 7.9, 4.6 Hz, 1H), 1.29-1.25 (m, 2H), 1.08 (dt, *J* = 7.9, 3.5 Hz, 2H); **^{13}C{^{1}H} NMR** ($CDCl_3$, 100 MHz): δ 200.2, 145.4, 139.9, 136.6, 128.9 (2C), 128.6 (2C), 128.1, 127.3 (2C), 127.1 (2C), 29.7, 17.1, 11.7; **FTIR** (thin film): cm^{-1} ; 3306, 3016, 2919, 2849, 1726, 1659, 1600, 1406, 1382, 1226, 990, 842, 740, 696; **HRMS** (ESI$^+$, m/z): Calcd for $C_{16}H_{15}O^+$ ([M+H]$^+$): 223.1117; found: 223.1109.

5. UV-Visible Spectroscopy Studies

A. 4-CzIPN absorption spectrum

4-CzIPN was diluted in acetonitrile and subjected to UV-Vis spectroscopy. MeCN (solvent) removed using double-beam-in-time UV-Vis spectrophotometer with MeCN blank.

Figure S1. 4-CzIPN UV-Vis Absorption Spectrum

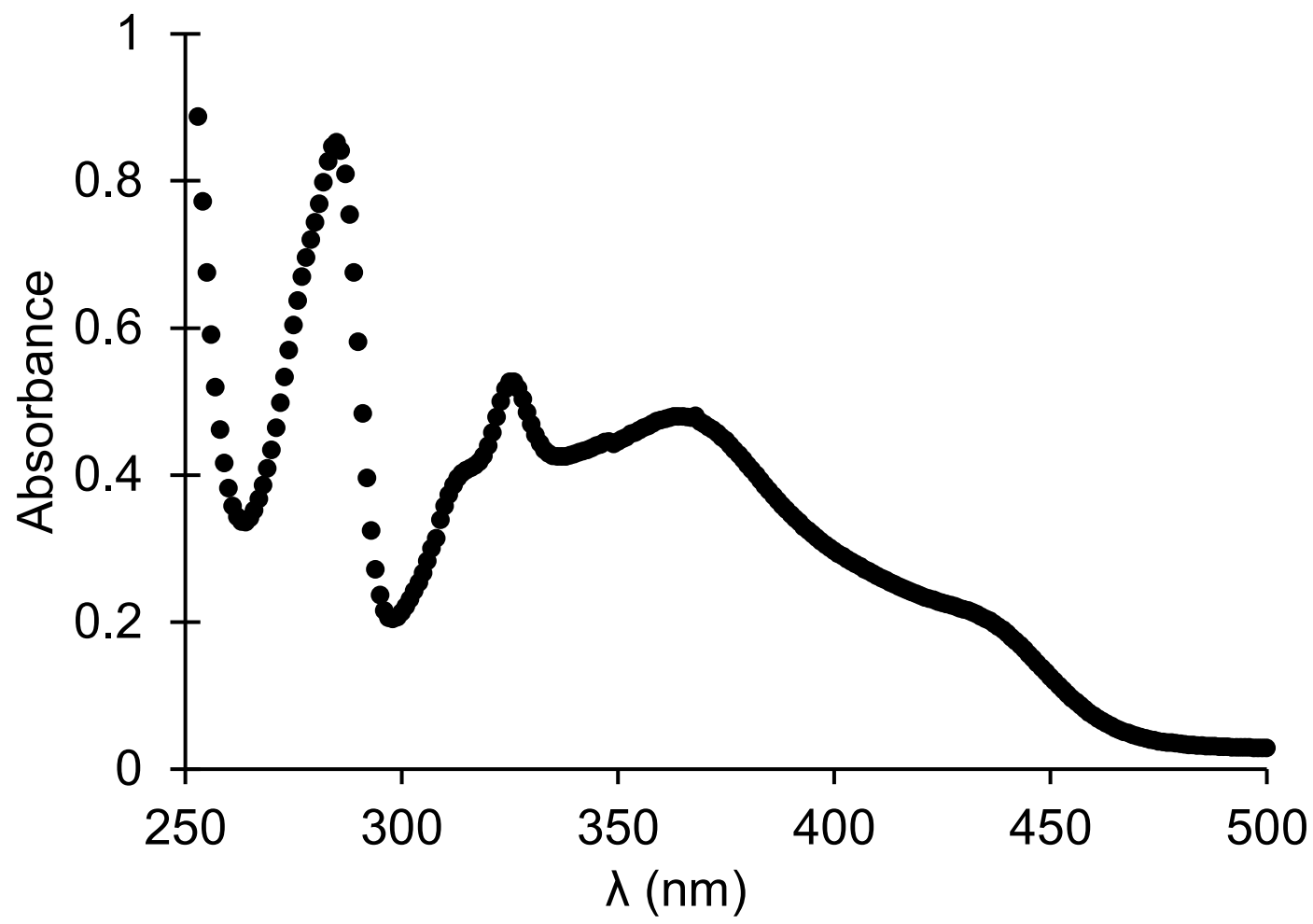

B. Oxoammonium trapping

To a 1-dram vial with a teflon stir bar was added TEMPO **2a** (15.6 mg, 0.1 mmol, 1 equiv), a stock solution of 4-CzIPN (0.10 mM in DCE, 0.5 mL, 0.00005 mmol, 0.0005 equiv), deionized H_2O (0.5 mL), and glacial acetic acid (22.3 µL, 0.2 mmol, 2 equiv). The vial was capped and stirred under irradiation of 400 nm light for 4 h. The aqueous phase was pipetted directly into a quartz cuvette, diluted with deionized water, and subjected to UV-Vis spectroscopy. Deionized H_2O (solvent) removed using double-beam-in-time UV-Vis instrument with deionized H_2O blank.

The spectrum was compared to 2,2,6,6-tetramethyl-1-oxopiperidinium tetrafluoroborate **2b** and commercially available TEMPO **2a**. Their absorption spectra were obtained by diluting the pure compound in acetonitrile and subjecting to UV-Vis spectroscopy. MeCN (solvent) removed using double-beam-in-time UV-Vis spectrophotometer with MeCN blank.

Figure S2. A) Overview of the oxoammonium trapping experiment and reaction conditions. B) UV absorption spectrum of the aqueous phase of the oxoammonium trapping experiment compared to TEMPO and oxoammonium standards.

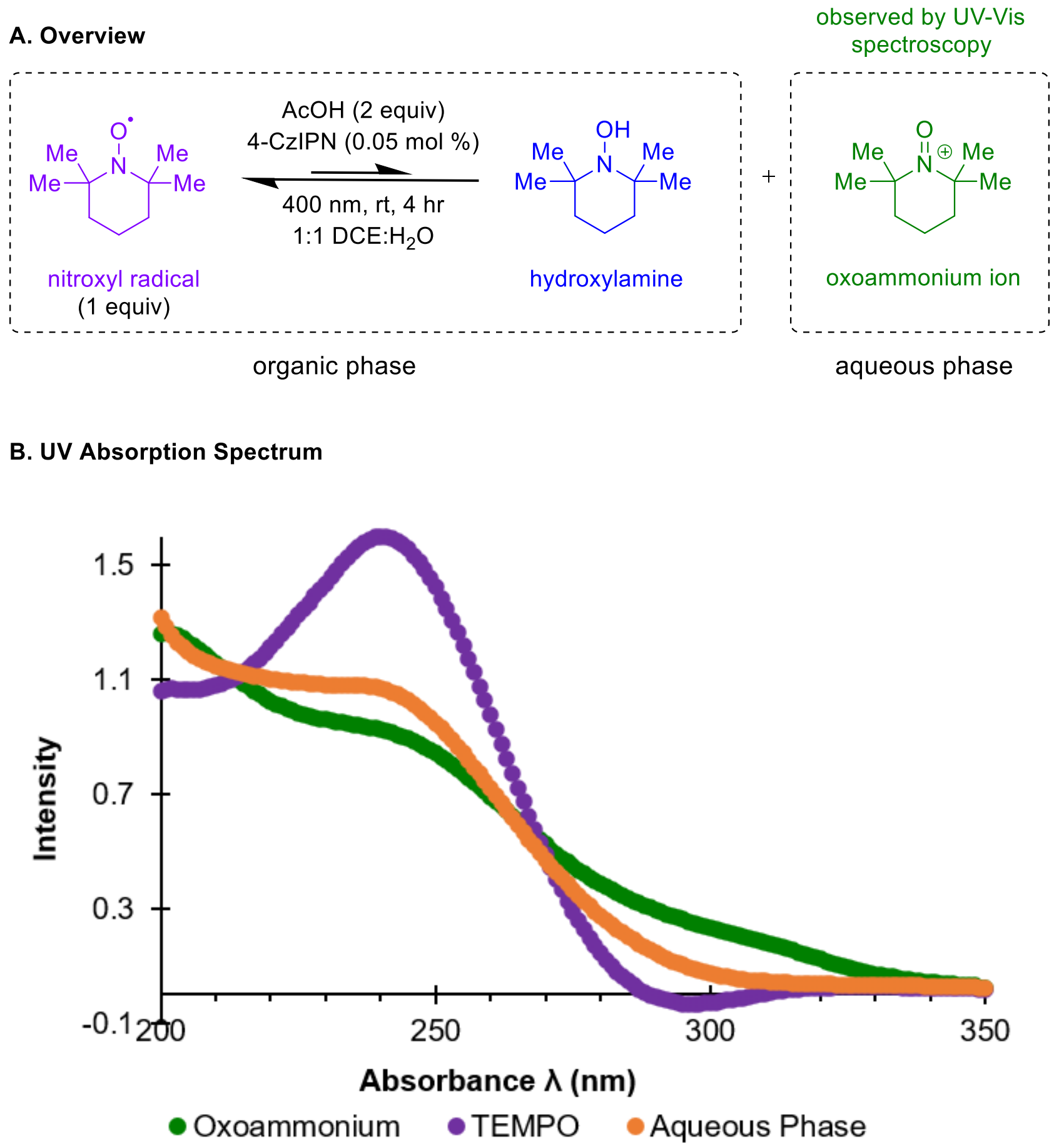

6. Fluorescence Spectroscopy Studies

A. 4-CzIPN emission spectrum

A solution of 4-CzIPN (80 μM in acetonitrile) was subjected to fluorescence spectroscopy with excitations at 365, 400, and 465 nm. An emission spectrum was collected from 450-650 nm with excitation and emission slits each at 10.0 nm. The fluorescence and phosphorescence bands are theorized to blend together in the emission spectrum because 4-CzIPN is a thermally-activated delayed fluorescence chromophore and has a very small energy difference between the S_1 and T_1 excited states.[14]

Table S2. 4-CzIPN Emissions vs Excitation Wavelength

Wavelength (nm)	Max Emission Intensity
365	2280
400	1920
465	456

Figure S3. An emission spectrum of 80 μM 4-CzIPN in MeCN excited at each of 400, 465, and 365 nm.

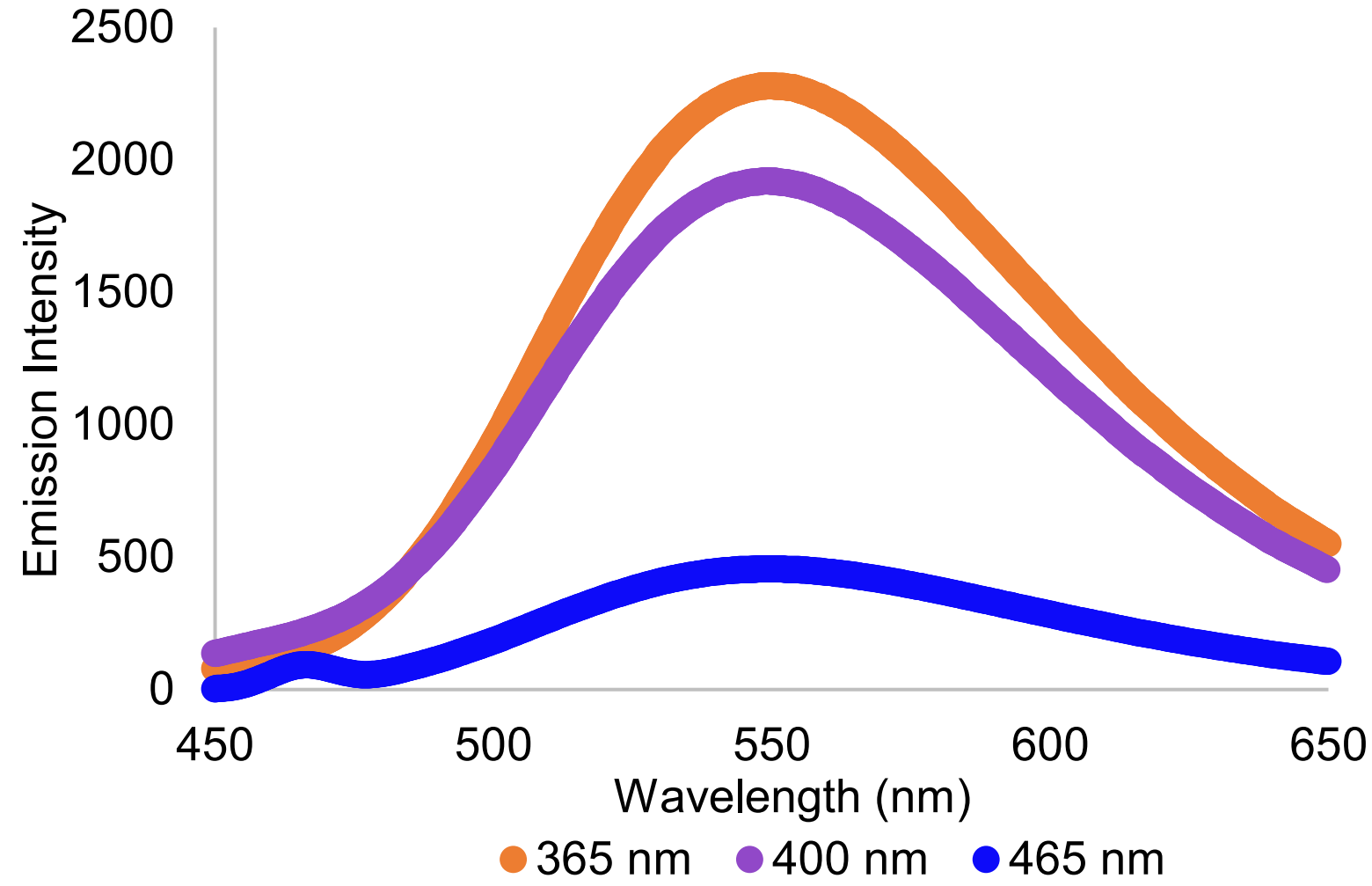

B. Photocatalytic Self-Quenching

4-CzIPN was diluted in acetonitrile at varying concentrations and subjected to fluorescence spectroscopy with excitation at 400 nm. Emission spectrum was collected from 450-650 nm with excitation and emission slits each 10.0 nm.

Table S3. 4-CzIPN Emissions vs Concentration

[4-CzIPN] (mM)	Max Emission Intensity
0.008	819
0.027	1597
0.080	1920
0.150	1856
0.220	1315
0.300	871

Figure S4. 4-CzIPN Emission Intensity with Respect to Concentration

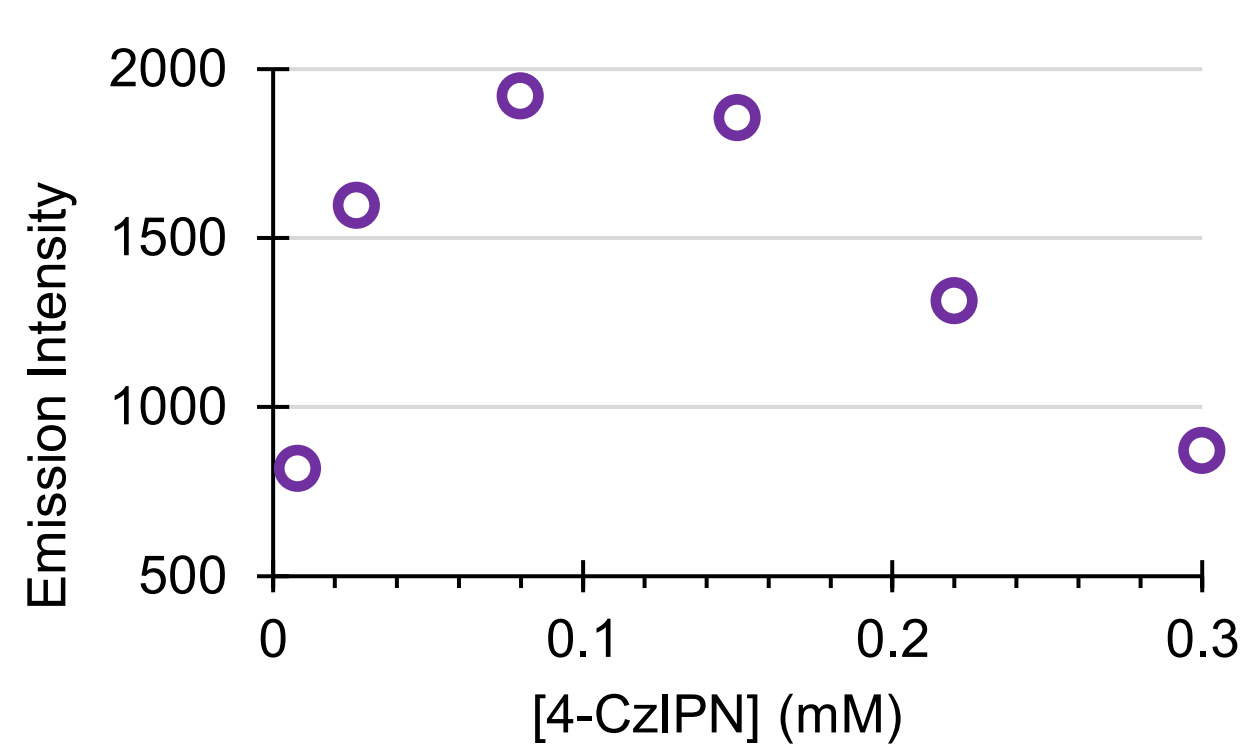

C. Stern-Volmer Luminescence Quenching Analysis

A 4-CzIPN stock solution (80 μM in MeCN) was prepared by dissolving 4-CzIPN (6.3 mg) in MeCN (100 mL). A quencher solution (0.10 M) was prepared by dissolving TEMPO (0.625 g, 4.0 mmol) in a portion of the 4-CzIPN stock solution (40 mL).

Each vial of varying concentrations of **2a** was prepared according to Table S3 and subjected to fluorescence spectroscopy in an aerobic atmosphere with excitation at 400 nm. Emission spectrum was collected from 450-650 nm with excitation and emission slits each 10.0 nm. The emission intensities at each respective concentration of **2a** (I) and quenching, calculated as I_0/I where I_0 is emission intensity in absence of quencher, are also listed.

Table S4. Stern-Volmer Solutions

Vial	[**2a**] (mM)	80 μM Stock Solution (mL)	0.10 M Quencher Solution (mL)	Total (mL)	**I**	**I_0/I**
1	0	1.50	0	1.5	1920	1.00
2	0.5	1.99	0.010	2	1664	1.15
3	1	1.98	0.020	2	1400	1.37
4	3	1.94	0.060	2	1071	1.79
5	25	1.13	0.375	1.505	264	7.27
6	50	0.75	0.750	1.5	100	19.2
7	75	0.38	1.125	1.505	58	33.1

Figure S5. Emission spectrum of 4-CzIPN in MeCN with 25, 50, and 75 mM TEMPO.

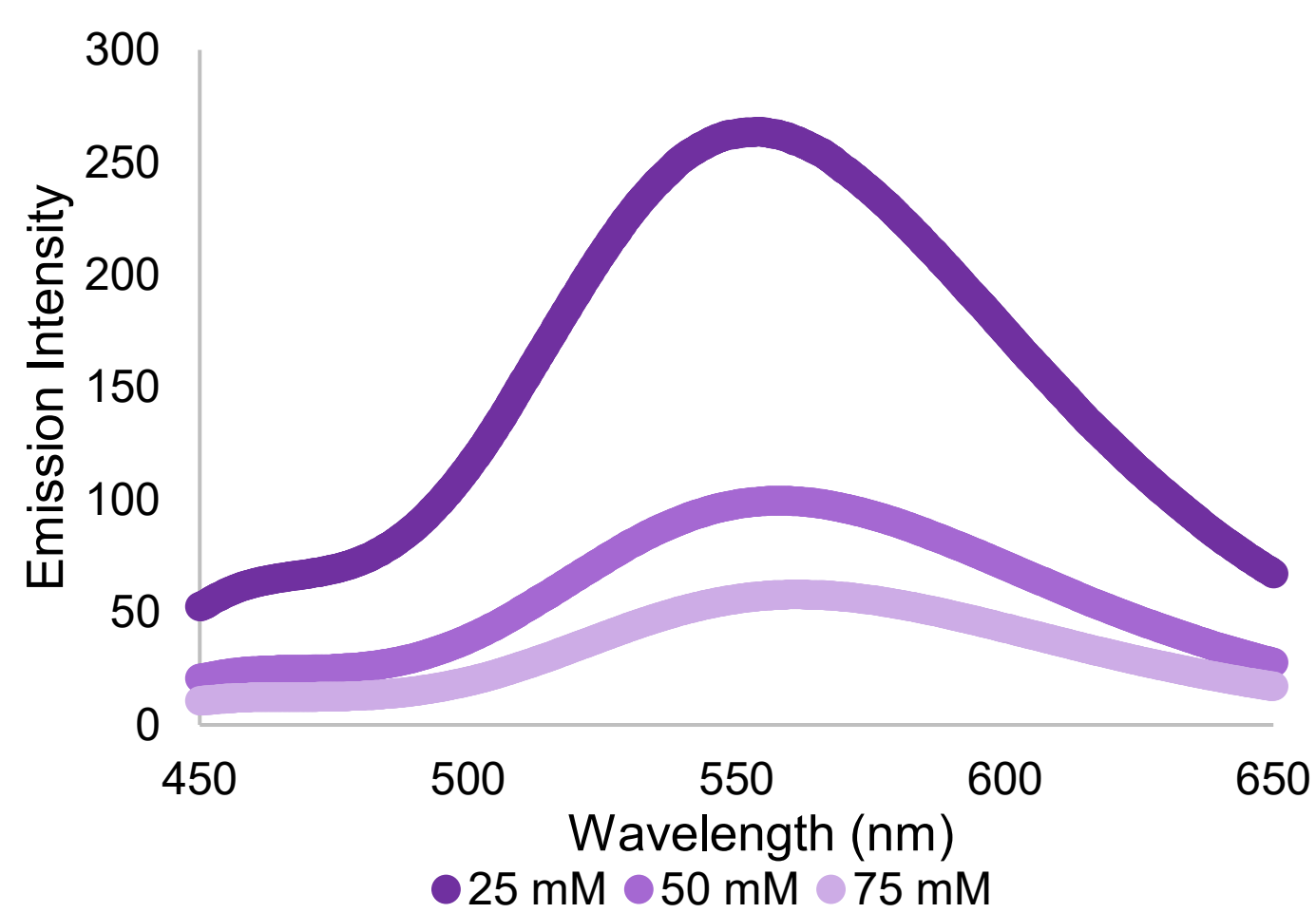

The linear Stern-Volmer equation (equation 1) demonstrates that there is a direct relationship between the degree of S_1 quenching (I_0/I) and the concentration of quencher [Q]. The slope of I_0/I vs [Q] represents the Stern Volmer constant (K^s_{SV}) which is the product of the S_1 quenching rate constant (k_s) and the decay rate of prompt fluorescence (τ_p) (equation 2). τ_p was obtained from the literature as 18.7 ns.[15]

$$I_0/I = K^s{}_{SV}[Q] + 1 \quad (1)$$

$$K^s{}_{SV} = k_s\tau_p \quad (2)$$

Figure S6A is a Stern-Volmer plot using quencher concentrations used in other photocatalytic studies.[16] While a significant correspondance between an increase in quenching and [Q] is observed, the slope gradually increases with [Q]. This is explained by the inner-filter effect: because TEMPO absorbs very slightly at 400 nm, the observed quenching increases with [Q].[17] To circumvent this, a Stern-Volmer plot using smaller quencher concentrations was assembled (Figure S6B). As expected, the slope decreased, verifying the significance of the inner-filter effect. k_s was calculated by K^{S}_{SV}/τ_p as 1.47×10^{10} M^{-1} s^{-1}, demonstrating significant S_1 quenching of 4-CzIPN by TEMPO as the kinetic driving force promoting TEMPO disproportionation.[16a]

Figure S6. Stern-Volmer luminescence quenching plots for A) vials 1-7 and B) data points obtained at low [Q] (vials 1-4) to remove inner-filter effect issues.

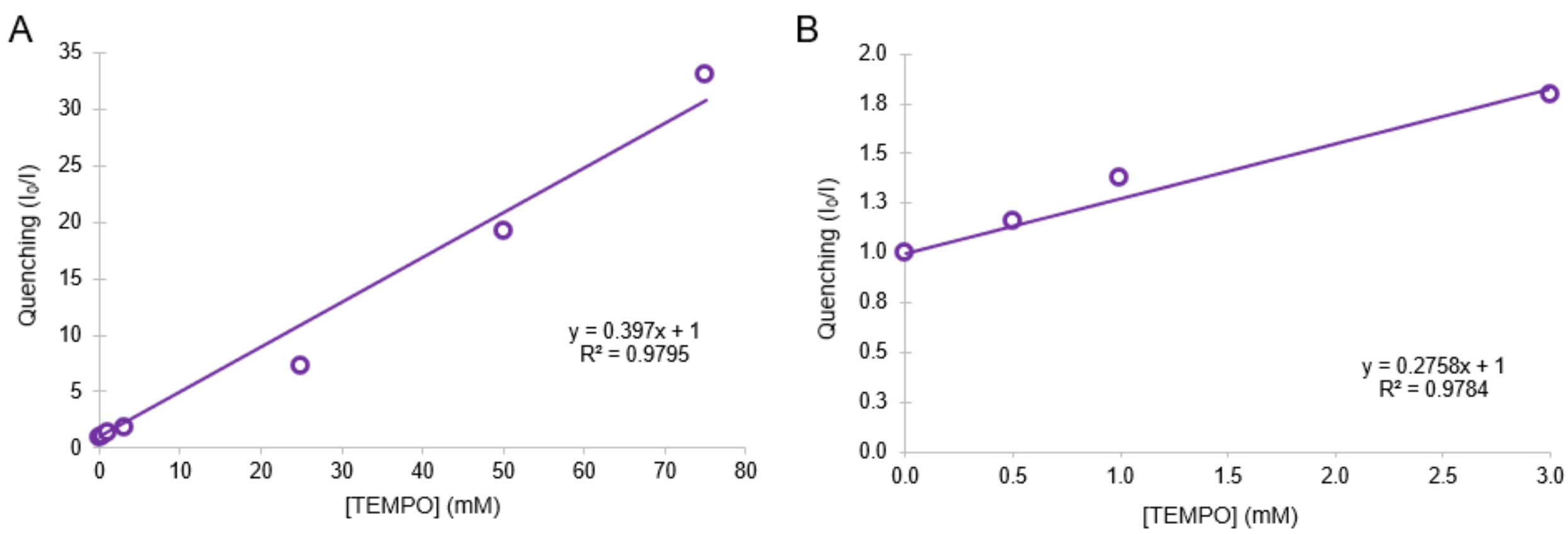

7. Light On/Off Experiment

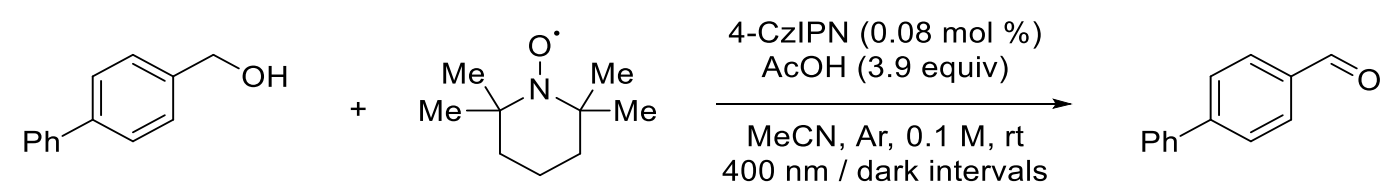

1a, 1 equiv **2a**, 2 equiv **3a**

To a 2-dram vial equipped with a Teflon-coated stir bar was added TEMPO **2a** (93.8 mg, 0.6 mmol, 2 equiv) and 4-biphenylmethanol **1a** (55.3 mg, 0.3 mmol, 1 equiv). The vial was capped with a rubber septum and sealed with parafilm. Using a needle inserted through the rubber septum, air was evacuated and backfilled with argon three times. Under argon using a 1 mL syringe, dry MeCN (2.75 mL) and a stock solution of 4-CzIPN (0.96 mM in MeCN, 0.25 mL, 0.00024 mmol, 0.0008 equiv) were added, followed by glacial acetic acid (67.0 µL, 69.7 mg, 1.16 mmol, 3.9 equiv) and 1,1,2,2-tetrachloroethane (31.55 µL, 0.3 mmol, 1 equiv) as an internal standard. The vial was stirred 800 rpm under irradiation of 400 nm light (2x 100 W LED panels, 1" from vial) which was powered on and off intermittently according to Table S4 and Figure S7. Room temperature was maintained using a circulating fan. Aliquots (0.20 mL) were obtained at the noted time intervals using a syringe with needle inserted through the rubber septum to maintain an inert atmosphere. These aliquots were inserted into an NMR tube alongside $CDCl_3$ (0.3 mL). The reaction was quenched by exposure to air and removal of light source. Reaction progress was monitored by quantitative ^{1}H NMR spectroscopy at a 45° pulse angle and 4 second relaxation delay with 16 scans using 1-peak solvent suppression. The resulting spectra were analyzed in MestReNova, with the 1,1,2,2-tetrachloroethane singlet peak (found at 5.95 ppm) set to a relative integration of 2.0.

Table S5. Light On/Off Experiment Data

Light	Aliquot	Time (min)	[**3a**] (M)
	0	0	0
on	1	20	0.0081
off	2	40	0.0083
on	3	60	0.0153
off	4	80	0.0155
on	5	105	0.0230

Figure S7. Light On/Off Experiment Plot

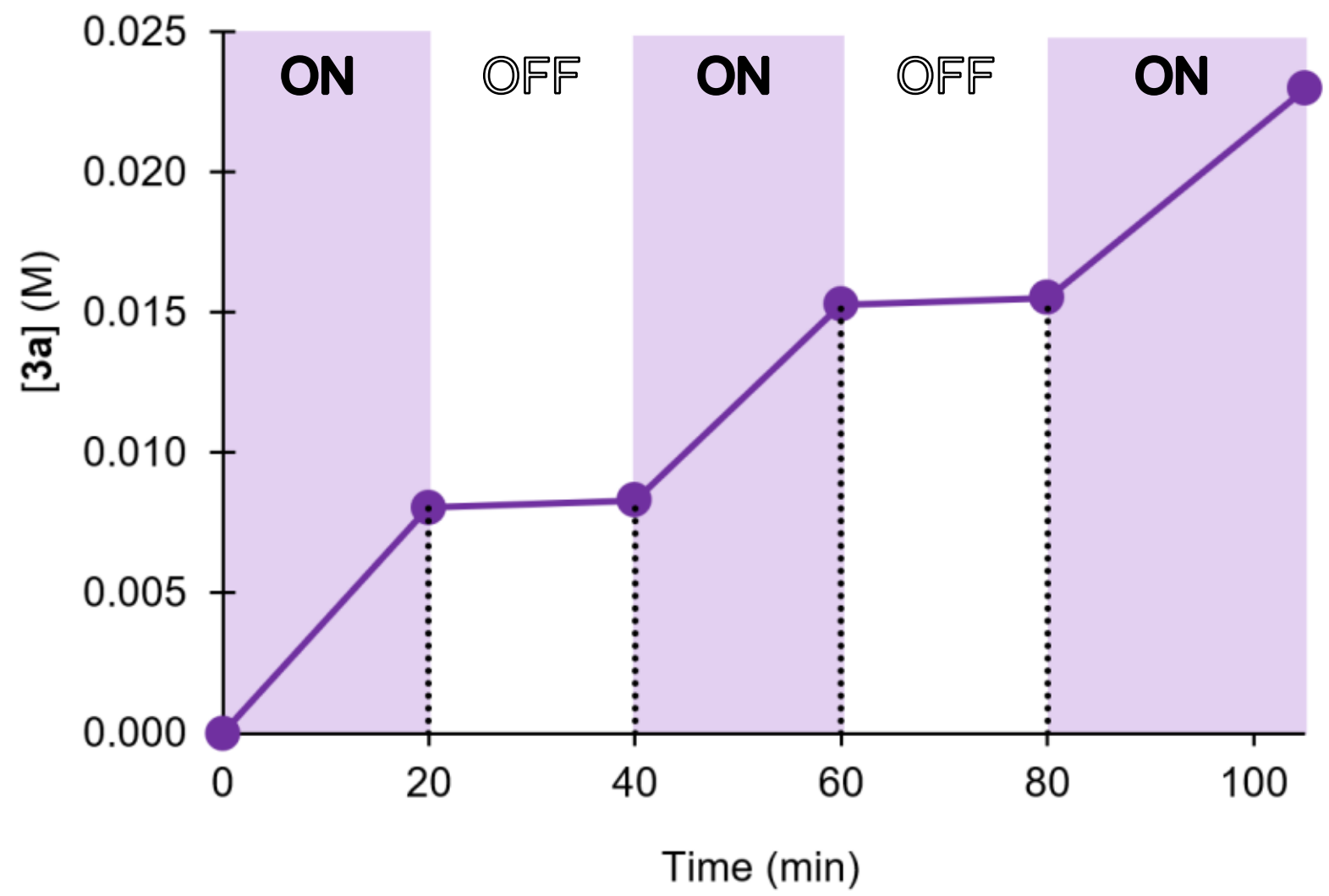

8. Determination of Partial Reaction Order in Acetic Acid.

These procedures were adapted from previous methods.[18]

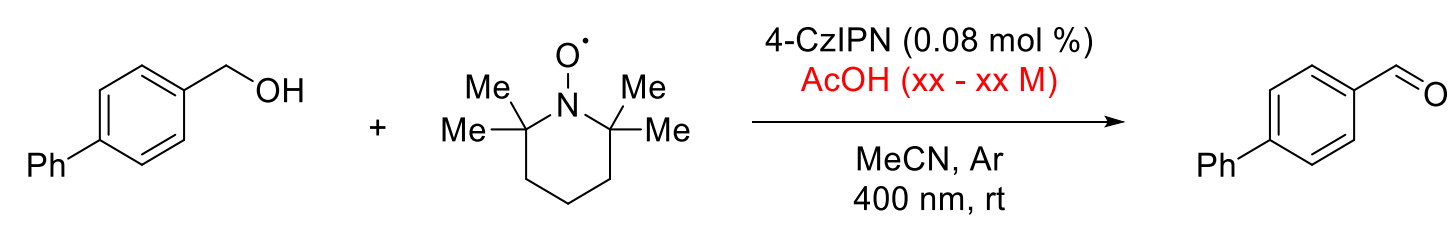

To a 2-dram vial equipped with a Teflon-coated stir bar was added TEMPO **2a** (93.8 mg, 0.6 mmol, 2 equiv) and 4-biphenylmethanol **1a** (55.3 mg, 0.3 mmol, 1 equiv). The vial was capped with a rubber septum and sealed with parafilm. Using a needle inserted through the rubber septum, air was evacuated and backfilled with argon three times. Under argon using a 1 mL syringe, dry MeCN (2.75 mL) and a stock solution of 4-CzIPN (0.96 mM in MeCN, 0.25 mL, 0.00024 mmol, 0.0008 equiv) were added, followed by glacial acetic acid (conc as noted below) and 1,1,2,2-tetrachloroethane (31.55 µL, 0.3 mmol, 1 equiv) as an internal standard. The vial was stirred 800 rpm under irradiation of 400 nm light (2x 100 W LED panels, 1" from vial) which was powered on and off intermittently according to Table S4 and Figure S7. Room temperature was maintained using a circulating fan. Aliquots (0.20 mL) were obtained at the noted time intervals using a

syringe with needle inserted through the rubber septum to maintain an inert atmosphere. These aliquots were inserted into an NMR tube alongside $CDCl_3$ (0.3 mL). The reaction was quenched by exposure to air and removal of light source. Reaction progress was monitored by quantitative ^{1}H NMR spectroscopy at a 45° pulse angle and 4 second relaxation delay with 16 scans using 1-peak solvent suppression. The resulting spectra were analyzed in MestReNova, with the 1,1,2,2-tetrachloroethane singlet peak (found at 5.95 ppm) set to a relative integration of 2.0.

Table S6. Preparation of Samples for Acetic Acid Rate Order Experiment (0.3 mmol scale)

Vial	[AcOH] (M)	AcOH (mmol)	Vol. AcOH (µL)
A	0.2	0.6	34.6
B	0.3	0.9	52.0
C	0.4	1.2	69.3
D	0.5	1.5	86.6
E	0.6	1.8	103.9

The reaction rate at each concentration was determined as the average rate between 0 and 13 minutes, measured by the formation of product (M min^{-1}).

Table S7. Partial Reaction Order in AcOH – A (0.2 M).

Time (min)	4	5.75	7.5	9.2	11.33	13
**3a (M)**	0.00420	0.00400	0.00439	0.00491	0.00528	0.00616

Figure S8. Partial Reaction Order in AcOH – A (0.2 M).

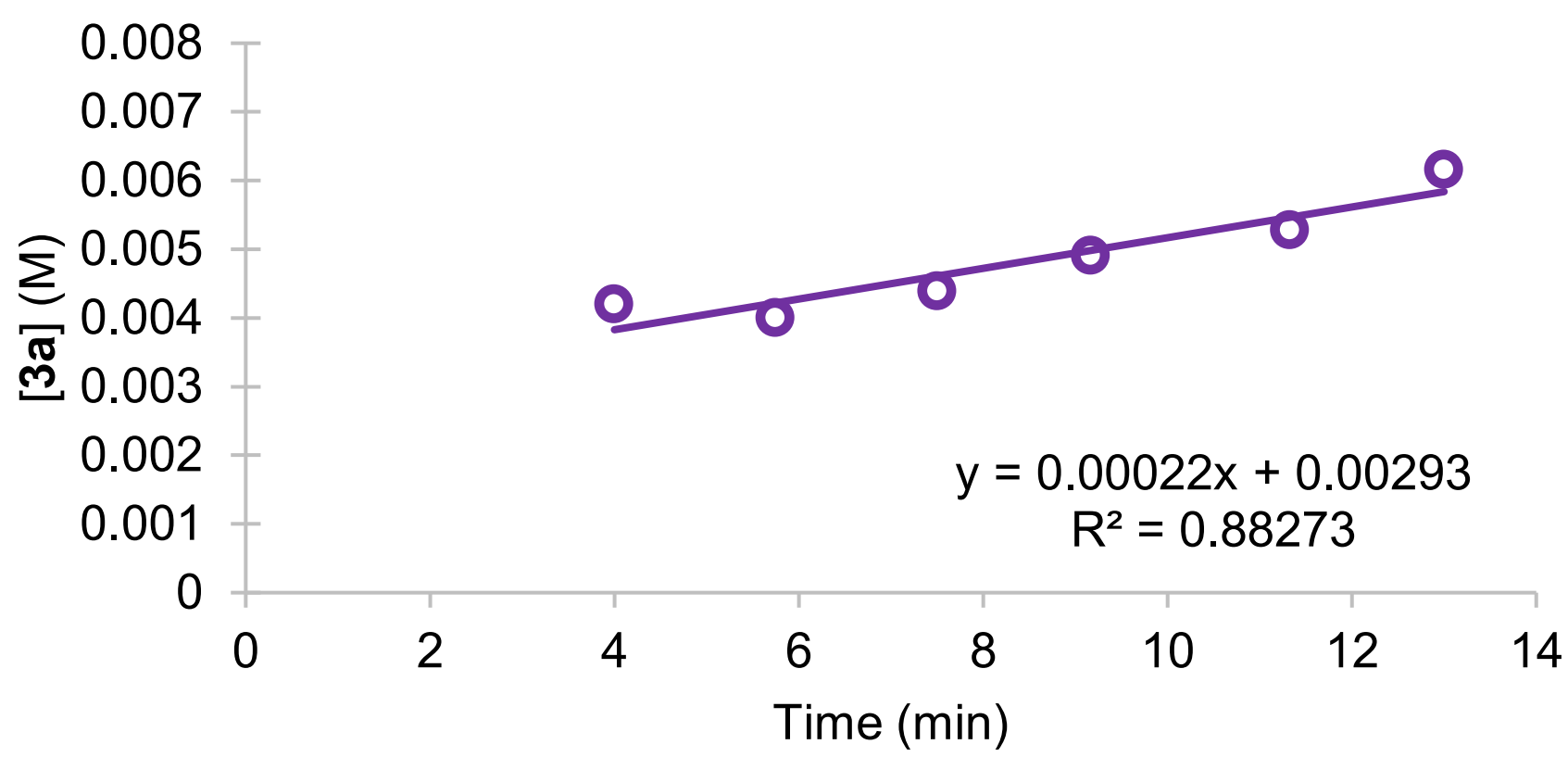

Table S8. Partial Reaction Order in AcOH – B (0.3 M).

Time (min)	1.27	4.33	6.38	8.42	10	11.75	13
**3a (M)**	0.00343	0.00391	0.00435	0.00506	0.00570	0.00618	0.00675

Figure S9. Partial Reaction Order in AcOH – B (0.3 M).

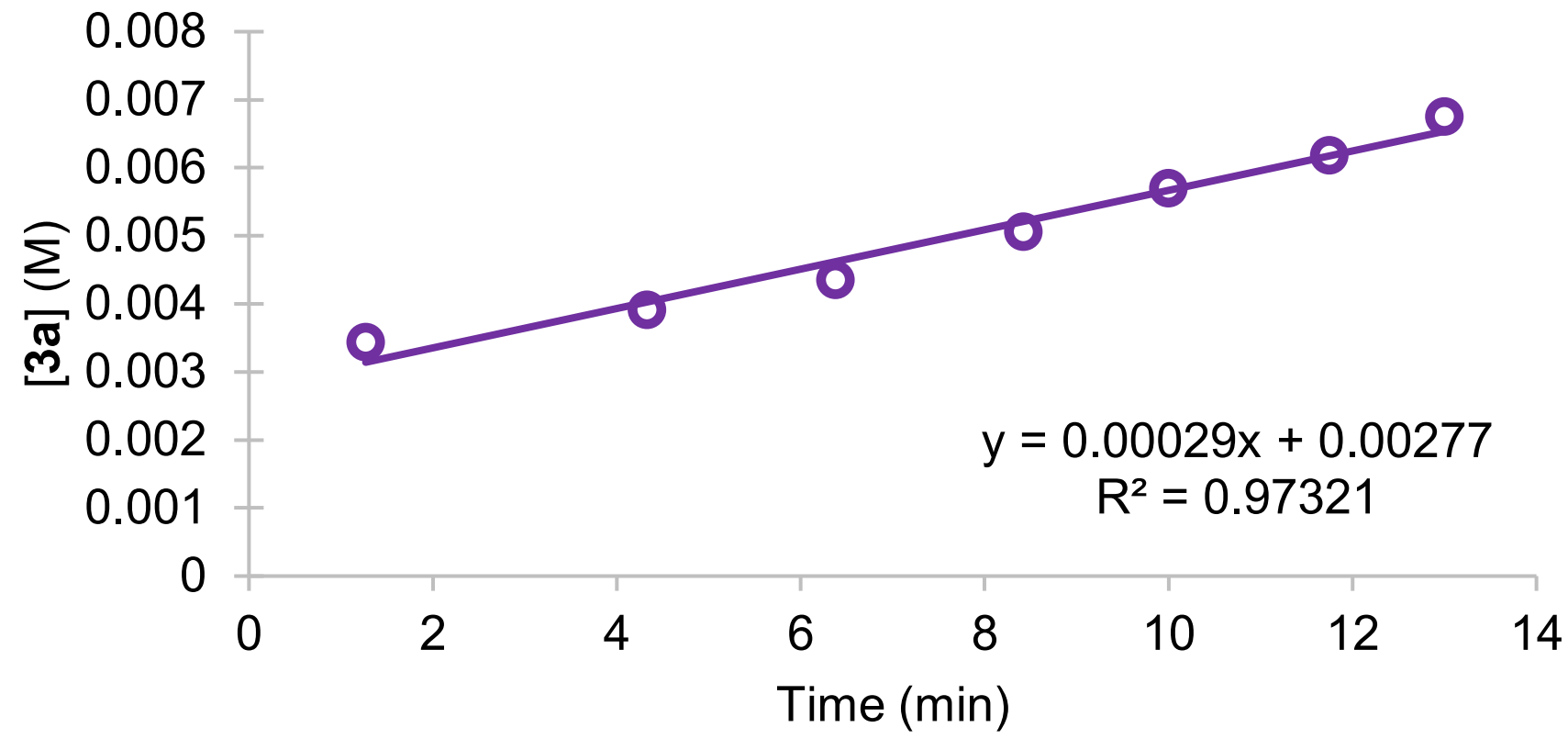

Table S9. Partial Reaction Order in AcOH – C (0.4 M).

Time (min)	3.25	4.75	6.75	8.5	10.2	12.3
**3a (M)**	0.00273	0.00348	0.00384	0.00436	0.00499	0.00614

Figure S10. Partial Reaction Order in AcOH – C. (0.4 M)

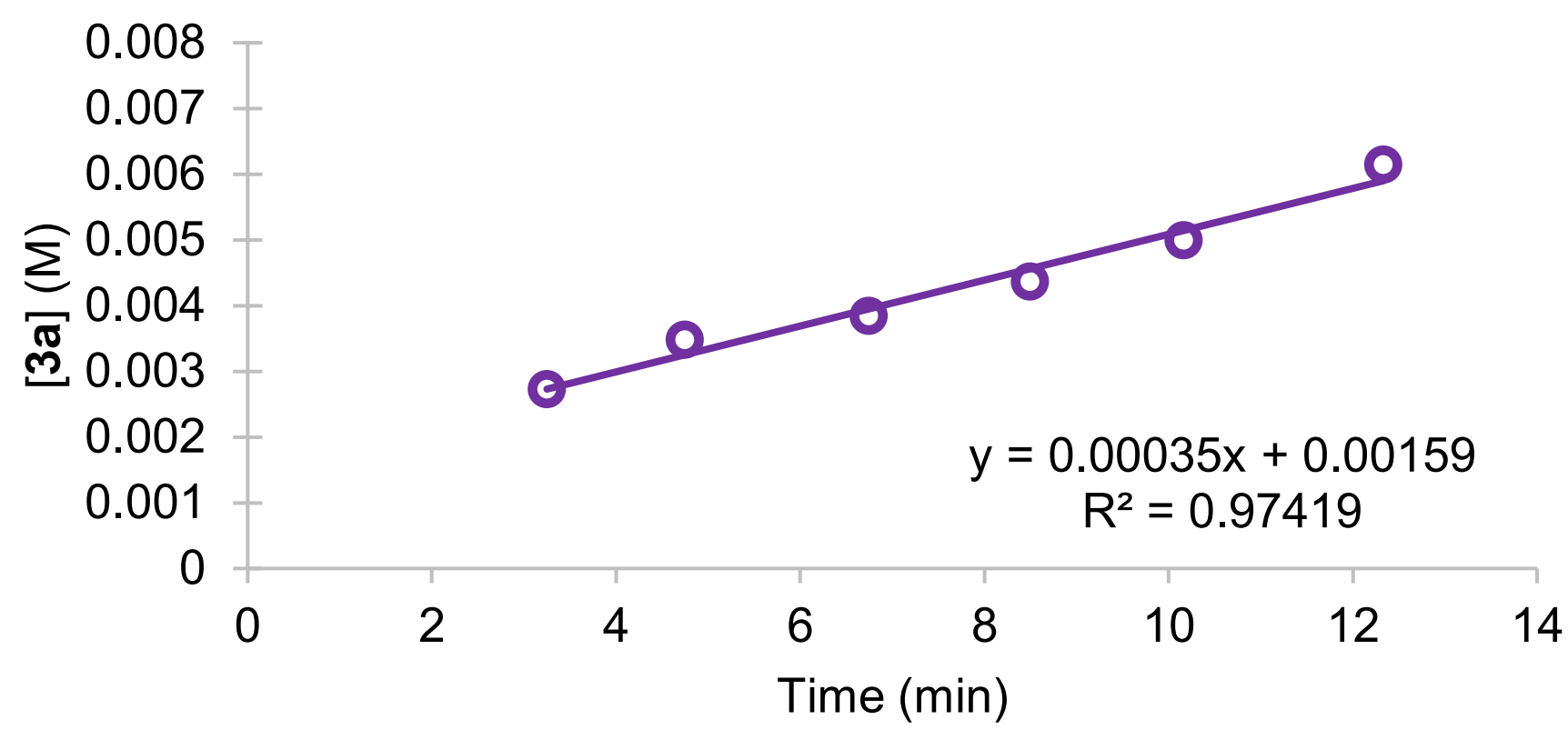

Table S10. Partial Reaction Order in AcOH – D. (0.5 M).

Time (min)	0.5	2	4.5	6	8	10
**3a (M)**	0.00184	0.00257	0.00342	0.00399	0.00462	0.00539

Figure S11. Partial Reaction Order in AcOH – D. (0.5 M)

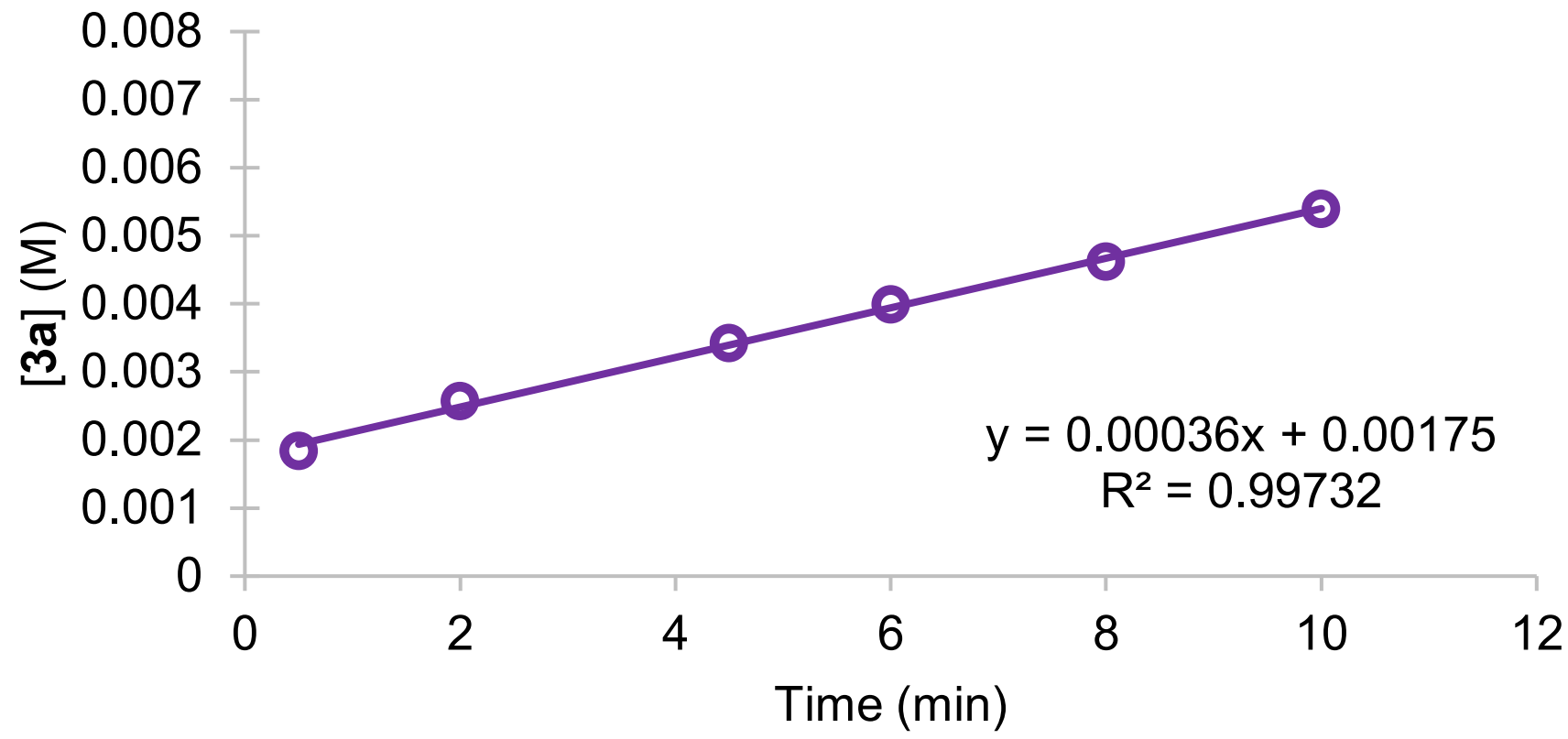

Table S11. Partial Reaction Order in AcOH – E. (0.6 M).

Time (min)	1.75	4.33	5.33	7.17	11.1
**3a (M)**	0.00461	0.00497	0.00531	0.00618	0.00818

Figure S12. Partial Reaction Order in AcOH – E. (0.6 M)

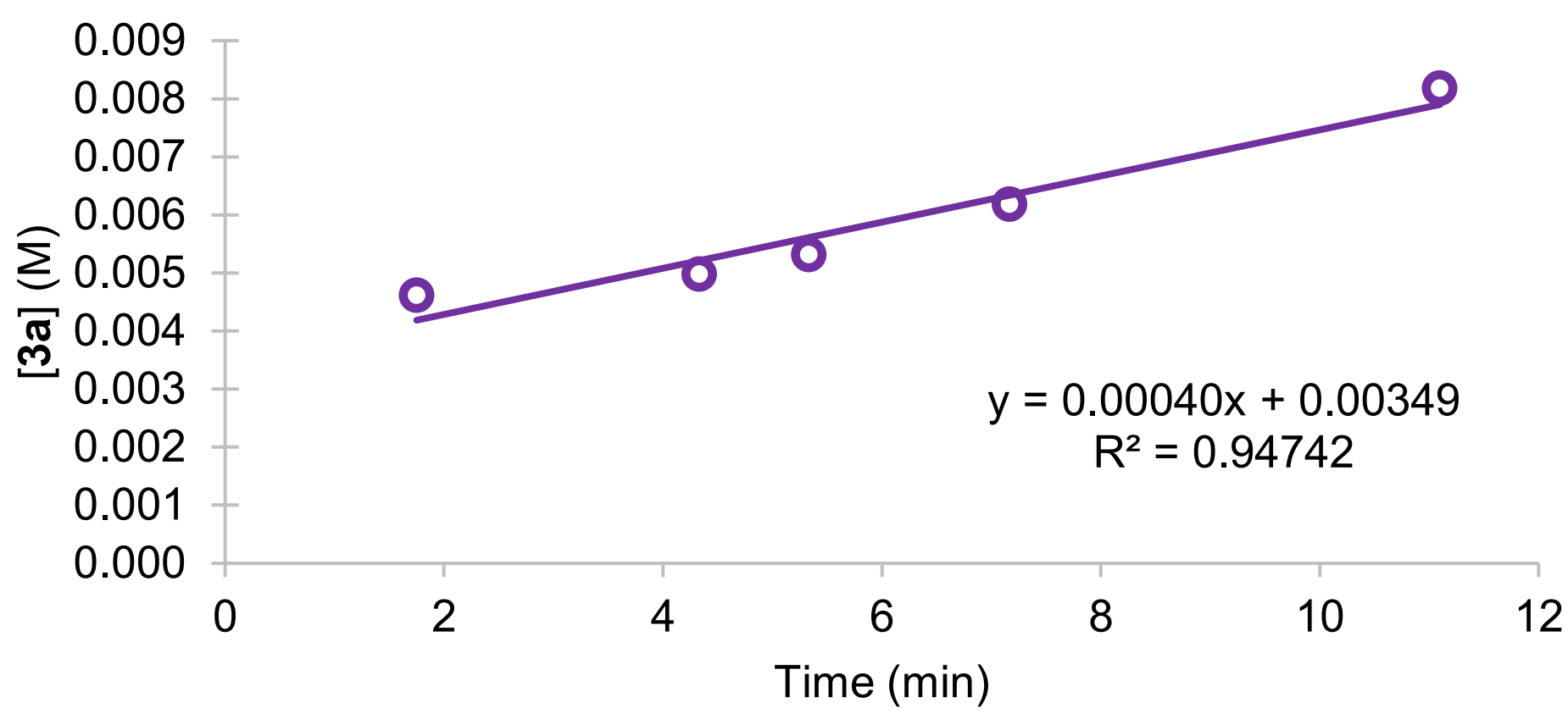

A general equation for the rate of this reaction is shown in equation 3.

$$Rate = k[ROH]^a[TEMPO]^b[AcOH]^c \tag{3}$$

The initial terms are all held constant and can be abbreviated as the observed rate constant k_{obs} for the reaction as shown in equation 4.

$$k[ROH]^a[TEMPO]^b = k_{obs} \tag{4}$$

Hence, this gives a form of the equation that can be modeled by a power fit as shown in equation 5 with [AcOH] as the dependent variable where *c* represents the partial reaction order in AcOH.

$$Rate = k_{obs}[AcOH]^c \tag{5}$$

The partial reaction order in AcOH was determined using a power fit of the initial reaction rates found for each concentration as shown in Figure S13.

Table S12. Rates for AcOH Concentrations for Rate Order Determination

AcOH [M]	0.2	0.3	0.4	0.5	0.6
Rate (M/min)	0.000223	0.000289	0.000349	0.000365	0.00397

Figure S13. Power Fit to Determine the Partial Reaction Order in AcOH. The partial reaction order in AcOH was determined to be 0.52.

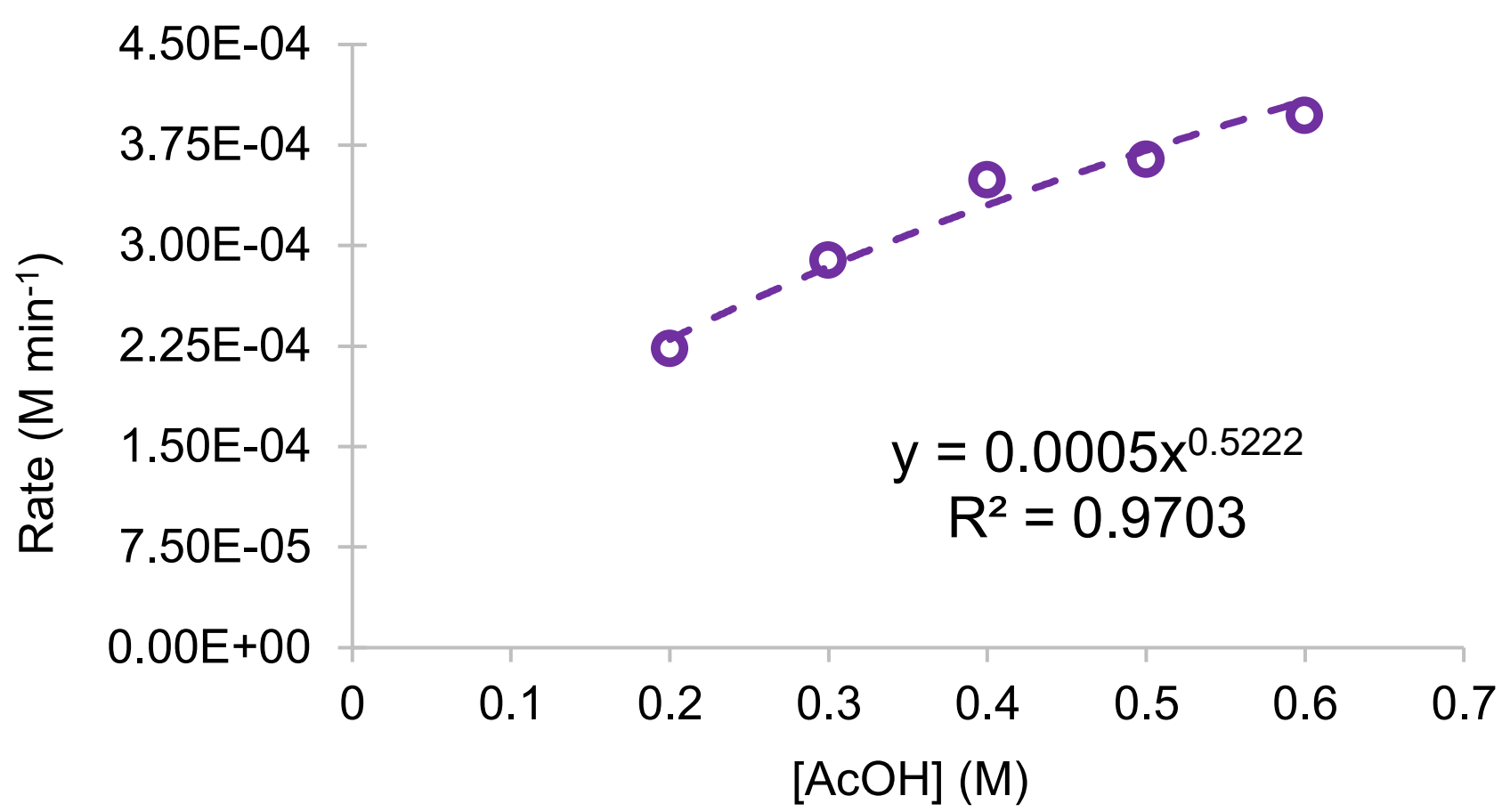

9. Kinetic Isotope Effect

To a 2-dram vial equipped with a Teflon-coated stir bar was added TEMPO **2a** (93.8 mg, 0.6 mmol, 2 equiv) and alcohol **1** (0.3 mmol, 1 equiv). The vial was capped with a rubber septum and sealed with parafilm. Using a needle inserted through the rubber septum, air was evacuated and backfilled with argon three times. Under argon using a 1 mL syringe, dry MeCN (2.75 mL) and a stock solution of 4-CzIPN (0.96 mM in MeCN, 0.25 mL, 0.00024 mmol, 0.0008 equiv) were added, followed by glacial acetic acid (67.0 µL, 69.7 mg, 1.16 mmol, 3.9 equiv) and 1,1,2,2-tetrachloroethane (31.55 µL, 0.3 mmol, 1 equiv) as an internal standard. The vial was stirred 800 rpm under irradiation of 400 nm light (2x 100 W LED panels, 1” from vial) which was powered on and off intermittently according to Table S4 and Figure S7. Room temperature was maintained using a circulating fan. Aliquots (0.20 mL) were obtained at the noted time intervals using a syringe with needle inserted through the rubber septum to maintain an inert atmosphere. These aliquots were inserted into an NMR tube alongside $CDCl_3$ (0.3 mL). The reaction was quenched by exposure to air and removal of light source. Reaction progress was monitored by quantitative ^{1}H NMR spectroscopy at a 45° pulse angle and 4 second relaxation delay with 16 scans using 1-peak solvent suppression. The resulting spectra were analyzed in MestReNova, with the 1,1,2,2-tetrachloroethane singlet peak (found at 5.95 ppm) set to a relative integration of 2.0.

Figure S14. Kinetic Isotope Effect (**3a** vs **3a-*d***)

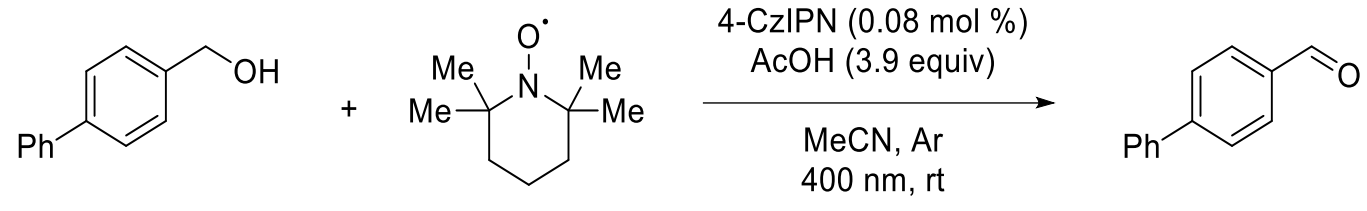

1a (0.1 M, 1 equiv) **2a** (0.2 M, 2 equiv) **3a**

Time (min)	4.33	17.5	25.75	35.5	45.67	56.5
**3a (M)**	0.00395	0.00635	0.00829	0.01033	0.01224	0.01381

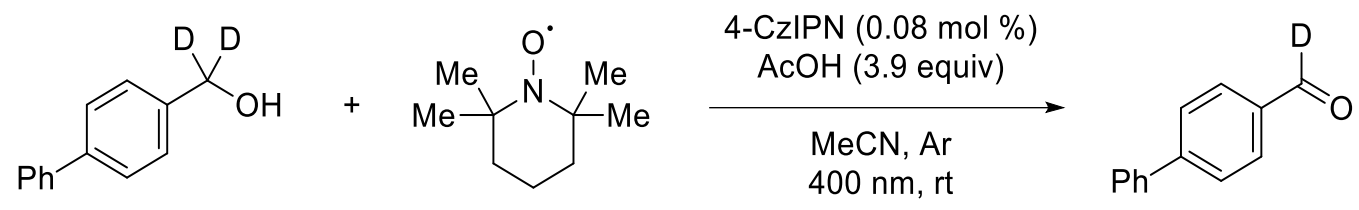

1a-*d*$_2$ (0.1 M, 1 equiv) **2a** (0.2 M, 2 equiv) **3a-*d***

Time (min)	5.83	18	27.2	36	47.17	57
**3a-*d* (M)**	0.00407	0.00556	0.00650	0.00778	0.00967	0.01169

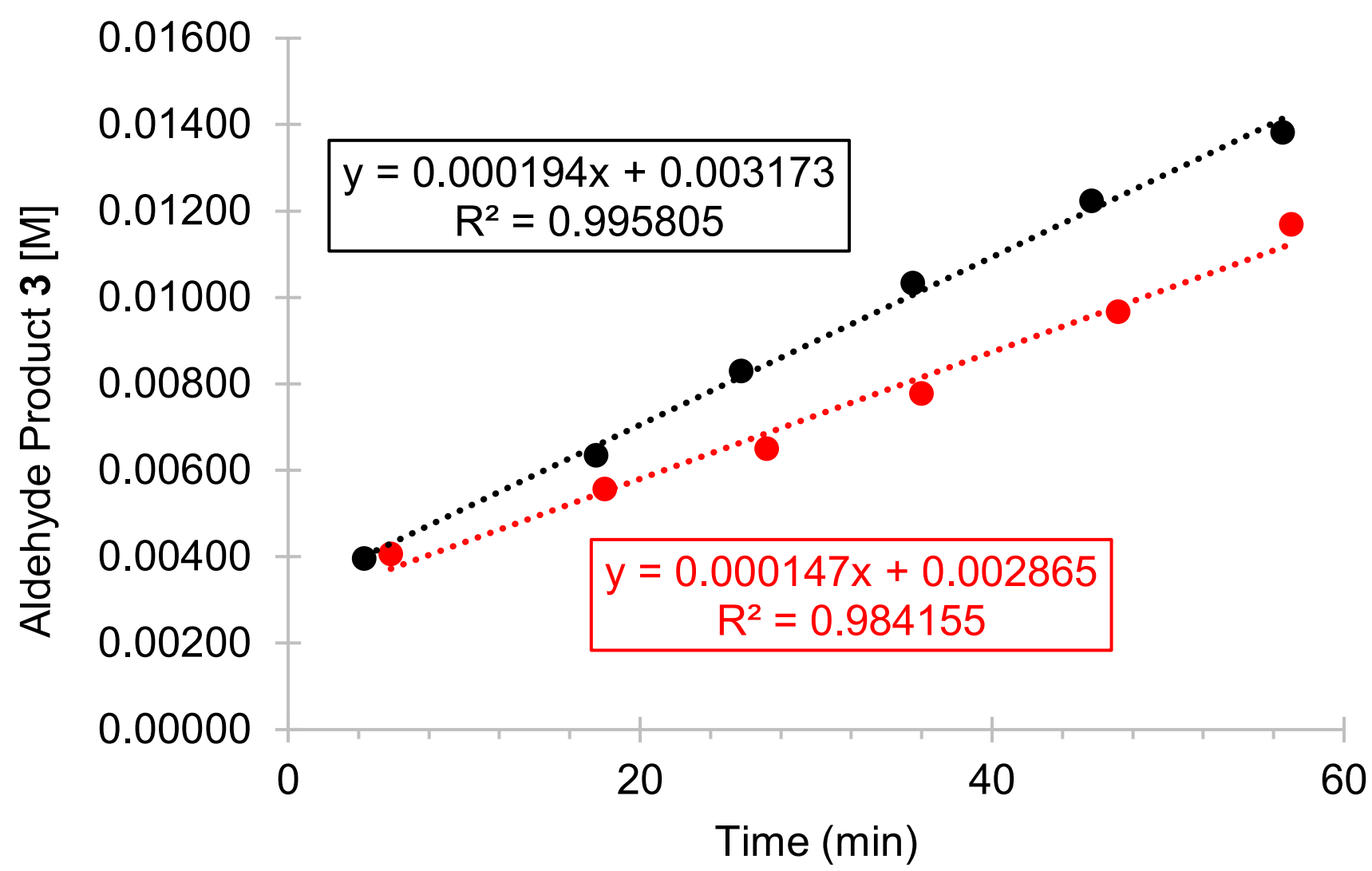

Using the general reaction rate order from equation 3, the ratio of these two reaction rates can be written as shown in equation 6.

$$\frac{Rate_H}{Rate_D} = \frac{k_H[TEMPO]^a[ROH]^b[AcOH]^c}{k_D[TEMPO]^a[ROH]^b[AcOH]^c} = \frac{k_H}{k_D} = \frac{0.000194}{0.000147} = 1.32 \qquad (6)$$

Hence, k_H/k_D was determined to be 1.32.

10. References

1. Khatua, H.; Das, S.; Patra, S.; Das, S. K.; Roy, S.; Chattopadhyay, B., "Iron-Catalyzed Intermolecular Amination of Benzylic C(sp3)–H Bonds." *J. Am. Chem. Soc.* **2022**, *144*, 21858-21866.
2. Jessen, H. J.; Schulz, T.; Balzarini, J.; Meier, C., "Bioreversible Protection of Nucleoside Diphosphates." *Angew. Chem. Int. Ed.* **2008**, *47*, 8719-8722.
3. Zhang, M.-J.; Tan, D.-W.; Li, H.-X.; Young, D. J.; Wang, H.-F.; Li, H.-Y.; Lang, J.-P., "Switchable Chemoselective Transfer Hydrogenations of Unsaturated Carbonyls Using Copper(I) N-Donor Thiolate Clusters." *J. Org. Chem.* **2018**, *83*, 1204-1215.
4. Wang, R.; Tang, Y.; Xu, M.; Meng, C.; Li, F., "Transfer Hydrogenation of Aldehydes and Ketones with Isopropanol under Neutral Conditions Catalyzed by a Metal–Ligand Bifunctional Catalyst [Cp*Ir(2,2′-bpyO)(H2O)]." *J. Org. Chem.* **2018**, *83*, 2274-2281.
5. Lölsberg, W.; Ye, S.; Schmalz, H.-G., "Enantioselective Copper-Catalysed Allylic Alkylation of Cinnamyl Chlorides by Grignard Reagents using Chiral Phosphine-Phosphite Ligands." *Adv. Synth. Catal.* **2010**, *352*, 2023-2031.
6. Hong, B.-C.; Tseng, H.-C.; Chen, S.-H., "Synthesis of aromatic aldehydes by organocatalytic [4+2] and [3+3] cycloaddition of α,β-unsaturated aldehydes." *Tetrahedron* **2007**, *63*, 2840-2850.
7. Lin, C.-K.; Lu, T.-J., "A simple method for the oxidation of primary alcohols with o-iodoxybenzoic acid (IBX) in the presence of acetic acid." *Tetrahedron* **2010**, *66*, 9688-9693.
8. Hayashi, M.; Shibuya, M.; Iwabuchi, Y., "Oxidation of Alcohols to Carbonyl Compounds with Diisopropyl Azodicarboxylate Catalyzed by Nitroxyl Radicals." *J. Org. Chem.* **2012**, *77*, 3005-3009.
9. Liu, J.; Zhu, J.; Jiang, H.; Wang, W.; Li, J., "Direct Oxidation of β-Aryl Substituted Aldehydes to α,β-Unsaturated Aldehydes Promoted by an o-Anisidine–Pd(OAc)2 Co-catalyst." *Chem. Asian J.* **2009**, *4*, 1712-1716.
10. (a) Ren, K.; Hu, B.; Zhao, M.; Tu, Y.; Xie, X.; Zhang, Z., "Ruthenium-Catalyzed Oxidation of Allyl Alcohols with Intermolecular Hydrogen Transfer: Synthesis of α,β-Unsaturated Carbonyl Compounds." *J. Org. Chem.* **2014**, *79*, 2170-2177; (b) Maity, A.; Hyun, S.-M.; Wortman, A. K.; Powers, D. C., "Oxidation Catalysis by an Aerobically Generated Dess–Martin Periodinane Analogue." *Angew. Chem. Int. Ed.* **2018**, *57*, 7205-7209; (c) Rasson, C.; Stouse, A.; Boreux, A.; Cirriez, V.; Riant, O., "Copper-Catalyzed One-Pot Borylative Aldolisation β-Fluoride Elimination for the Formal Addition of Acrylates to Carbonyl Moieties." *Chem. Eur. J.* **2018**, *24*, 9234-9237.
11. Lin, D.; Lv, L.; Wang, J.; Ding, X.; Jiang, H.; Liu, H., "Preparation of α-Alkyl-β-Amino Acids via β-Alanine Ni(II) Complex." *J. Org. Chem.* **2011**, *76*, 6649-6656.
12. Wiseman, J. M.; McDonald, F. E.; Liotta, D. C., "1-Deoxy-5-hydroxysphingolipids as New Anticancer Principles: An Efficient Procedure for Stereoselective Syntheses of 2-Amino-3,5-diols." *Org. Lett.* **2005**, *7*, 3155-3157.
13. Chen, S.; Liu, Z.; Shi, E.; Chen, L.; Wei, W.; Li, H.; Cheng, Y.; Wan, X., "Ruthenium-Catalyzed Oxidation of Alkenes at Room Temperature: A Practical and Concise Approach to α-Diketones." *Org. Lett.* **2011**, *13*, 2274-2277.
14. (a) Sun, L.; Hua, W.; Liu, Y.; Tian, G.; Chen, M.; Chen, M.; Yang, F.; Wang, S.; Zhang, X.; Luo, Y.; Hu, W., "Thermally Activated Delayed Fluorescence in an Organic Cocrystal: Narrowing the Singlet–Triplet Energy Gap via Charge Transfer." *Angew. Chem. Int. Ed.* **2019**, *58*, 11311-11316; (b) Fina, F.; Bellatreccia, C.; Wu, X.; Cozzi, P. G.; Troisi, A.; Vinogradov, S.; Ceroni, P., "Reactivity of organic photocatalysts displaying thermally activated delayed fluorescence (TADF): rationalizing unexpected differences between rates of quenching of the lowest singlet and triplet states." *Chem. Sci.* **2025**, *16*, 20536-20543.
15. Sneha, M.; Thornton, G. L.; Lewis-Borrell, L.; Ryder, A. S. H.; Espley, S. G.; Clark, I. P.; Cresswell, A. J.; Grayson, M. N.; Orr-Ewing, A. J., "Photoredox-HAT Catalysis for Primary Amine α-C–H Alkylation: Mechanistic Insight with Transient Absorption Spectroscopy." *ACS Catal.* **2023**, *13*, 8004-8013.
16. (a) Motz, R. N.; Sun, A. C.; Lehnherr, D.; Ruccolo, S., "High-Throughput Determination of Stern–Volmer Quenching Constants for Common Photocatalysts and Quenchers." *ACS Organic & Inorganic Au* **2023**, *3*, 266-273; (b) Seliger, J.; Fries, L. R.; Meinhardt, J. M.; Knowles, R. R., "Photoredox-Catalyzed Nucleophilic Aromatic Substitution of Halophenols with Azoles via Oligomeric Phenylene Oxide Radicals." *J. Am. Chem. Soc.* **2025**, *147*, 9931-9938; (c) Stache, E. E.; Ertel, A. B.; Rovis, T.; Doyle, A. G., "Generation of Phosphoranyl Radicals via Photoredox Catalysis Enables Voltage–Independent Activation of Strong C–O Bonds." *ACS Catal.* **2018**, *8*, 11134-11139.
17. Weitner, T.; Friganović, T.; Šakić, D., "Inner Filter Effect Correction for Fluorescence Measurements in Microplates Using Variable Vertical Axis Focus." *Analytical Chemistry* **2022**, *94*, 7107-7114.
18. Delaney, C. P.; Marron, D. P.; Shved, A. S.; Zare, R. N.; Waymouth, R. M.; Denmark, S. E., "Potassium Trimethylsilanolate-Promoted, Anhydrous Suzuki–Miyaura Cross-Coupling Reaction Proceeds via the “Boronate Mechanism”: Evidence for the Alternative Fork in the Trail." *J. Am. Chem. Soc.* **2022**, *144*, 4345-4364.

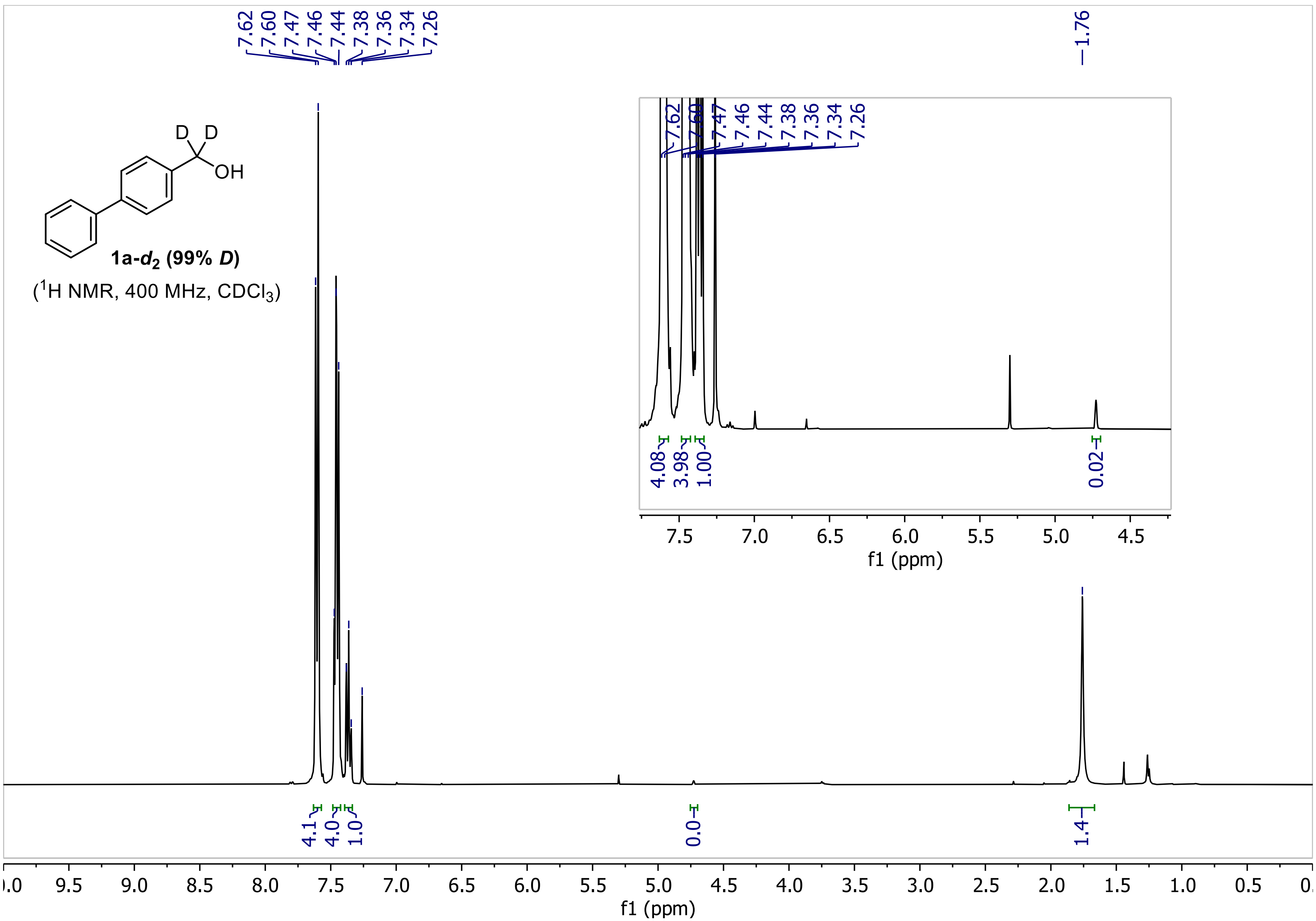
7.62 7.60 7.47 7.46 7.44 7.38 7.36 7.34 7.26
1.76
D D
OH
1a-d_2 (99% D)
(^{1}H NMR, 400 MHz, $CDCl_3$)
7.62 7.60 7.47 7.46 7.44 7.38 7.36 7.34 7.26
4.08 3.98 1.00
0.02
7.5 7.0 6.5 6.0 5.5 5.0 4.5
f1 (ppm)
4.1 4.0 1.0
0.0
1.4
10.0 9.5 9.0 8.5 8.0 7.5 7.0 6.5 6.0 5.5 5.0 4.5 4.0 3.5 3.0 2.5 2.0 1.5 1.0 0.5 0
f1 (ppm)

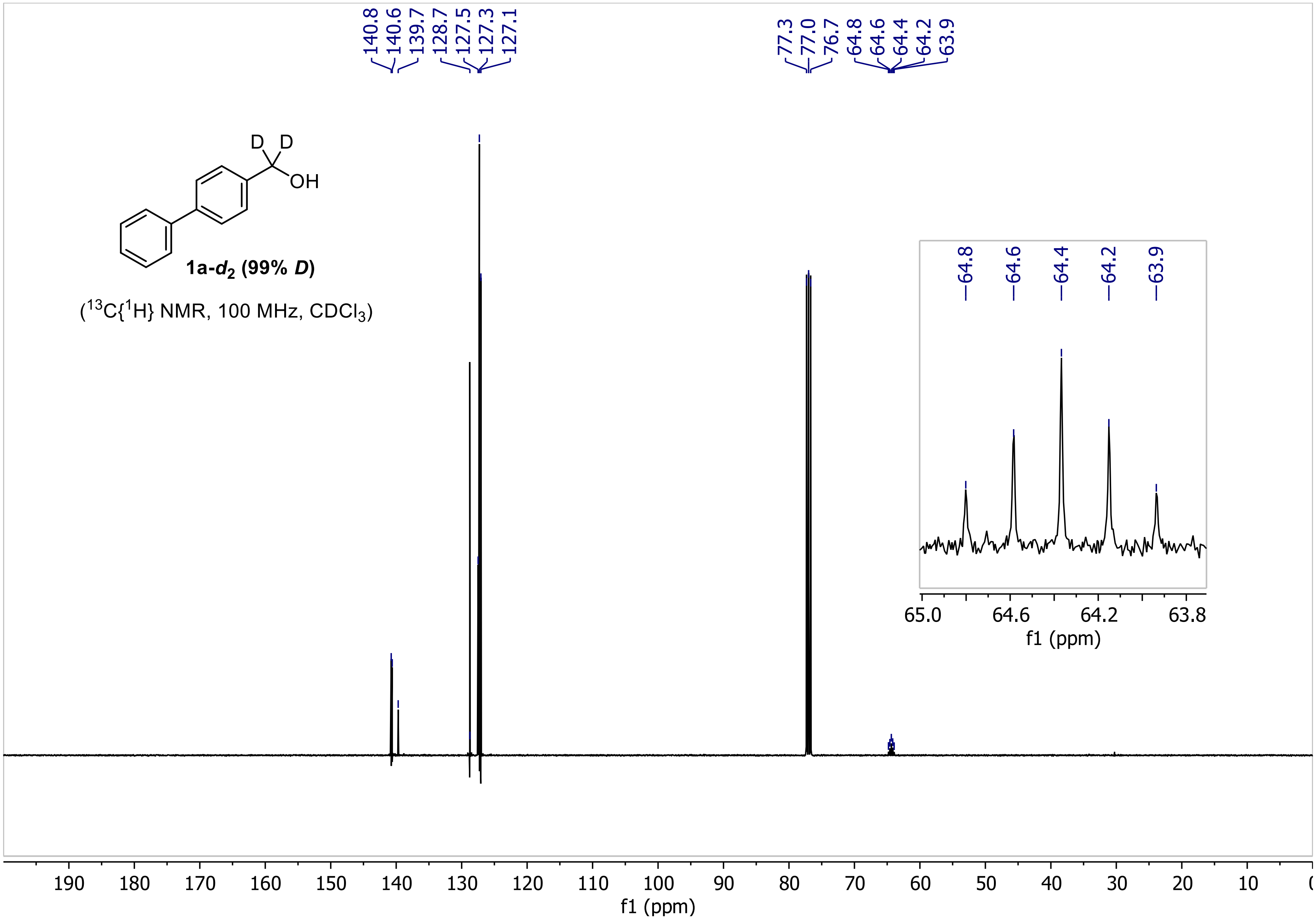

140.8
140.6
139.7
128.7
127.5
127.3
127.1
77.3
77.0
76.7
64.8
64.6
64.4
64.2
63.9
D D
OH
1a-*d*₂ (99% *D*)
($^{13}C\{^{1}H\}$ NMR, 100 MHz, $CDCl_3$)
65.0
64.6
64.2
63.8
f1 (ppm)
190 180 170 160 150 140 130 120 110 100 90 80 70 60 50 40 30 20 10 0
f1 (ppm)

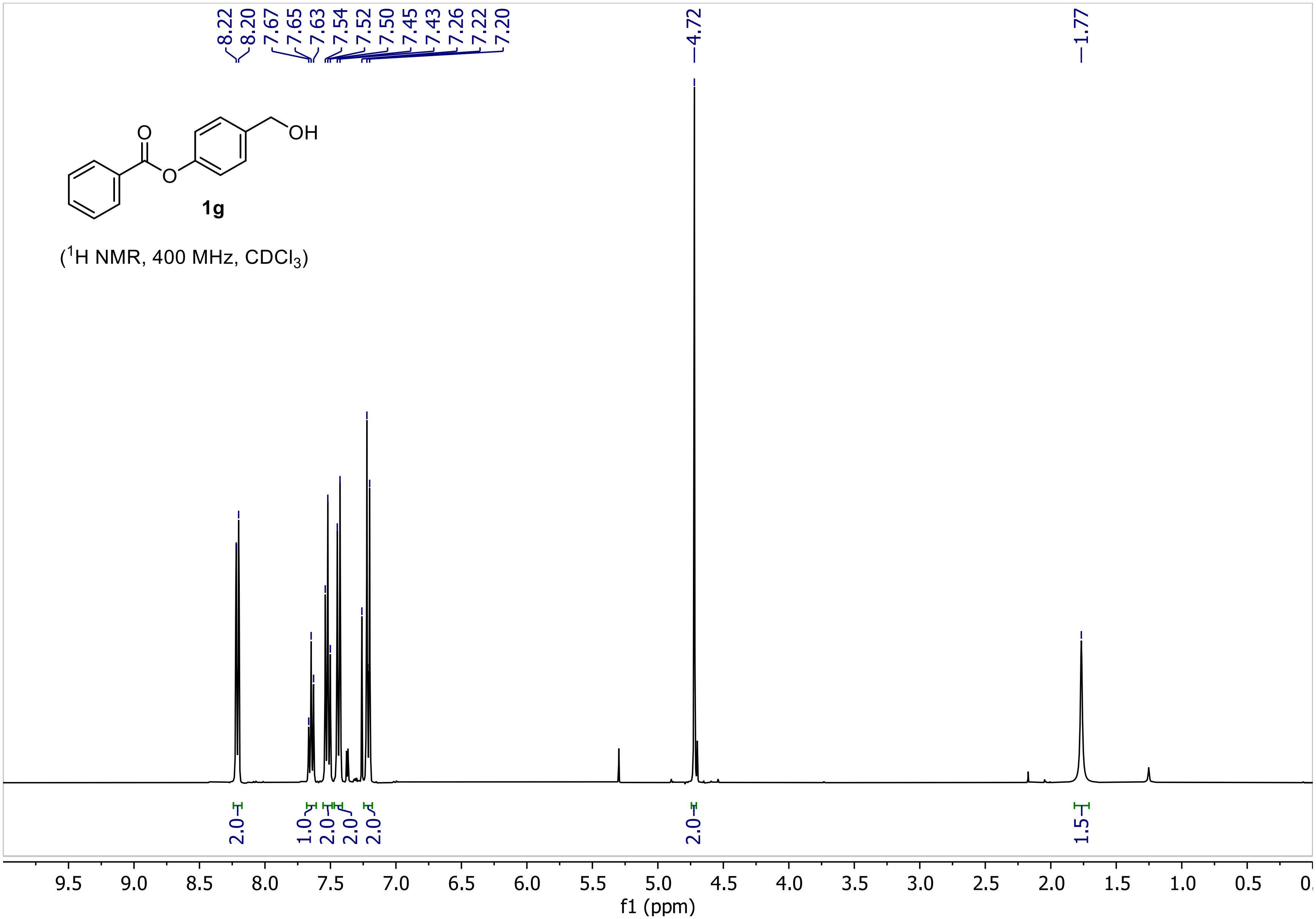
8.22
8.20
7.67
7.65
7.63
7.54
7.52
7.50
7.45
7.43
7.26
7.22
7.20
4.72
1.77
OH
O
O
1g
(1H NMR, 400 MHz, CDCl3)
2.0
1.0
2.0
2.0
2.0
2.0
1.5
9.5
9.0
8.5
8.0
7.5
7.0
6.5
6.0
5.5
5.0
4.5
4.0
3.5
3.0
2.5
2.0
1.5
1.0
0.5
f1 (ppm)

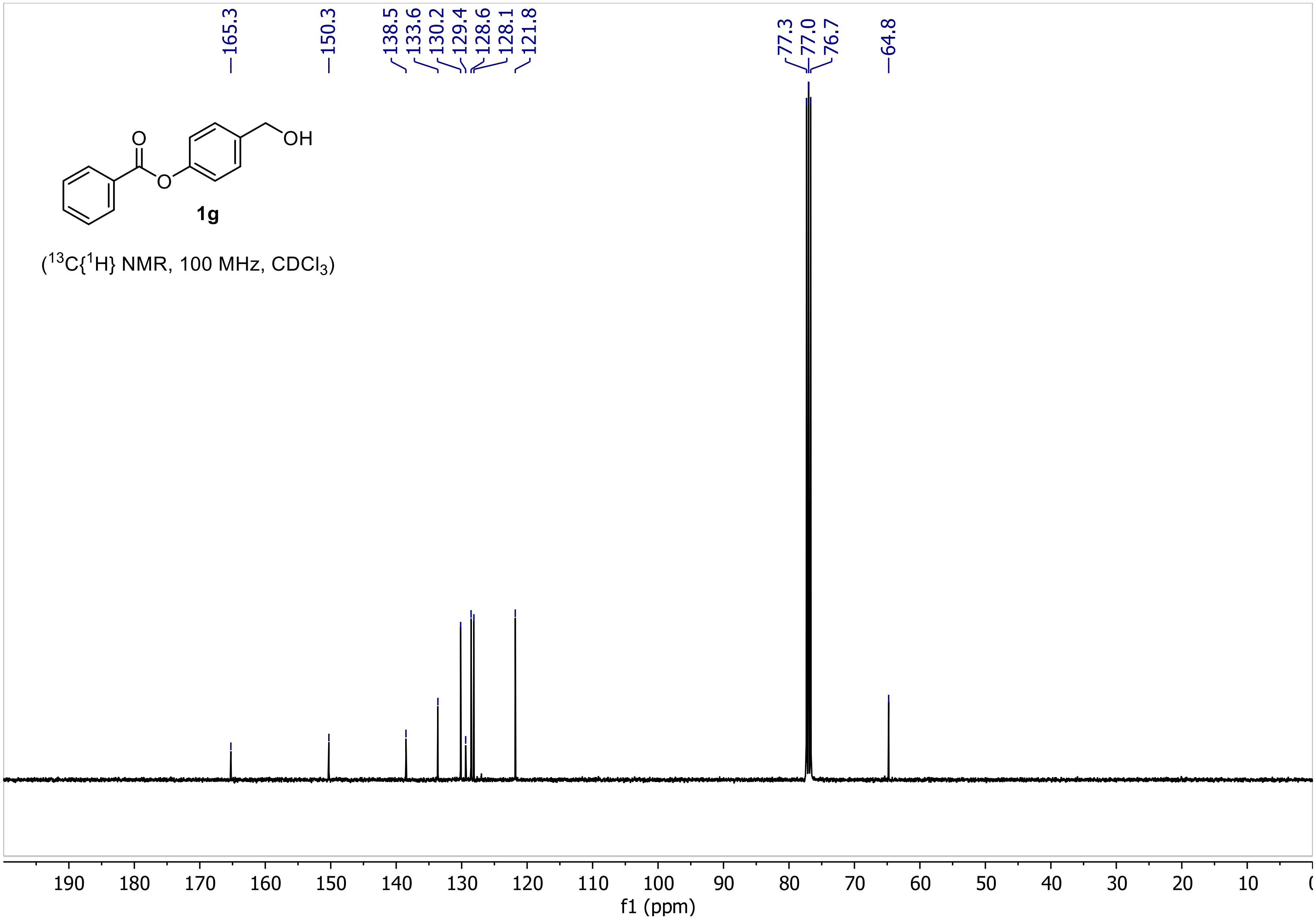
165.3
150.3
138.5
133.6
130.2
129.4
128.6
128.1
121.8
77.3
77.0
76.7
64.8
O
OH
O
1g
($^{13}C\{^{1}H\}$ NMR, 100 MHz, $CDCl_3$)
190
180
170
160
150
140
130
120
110
100
90
80
70
60
50
40
30
20
10
f1 (ppm)

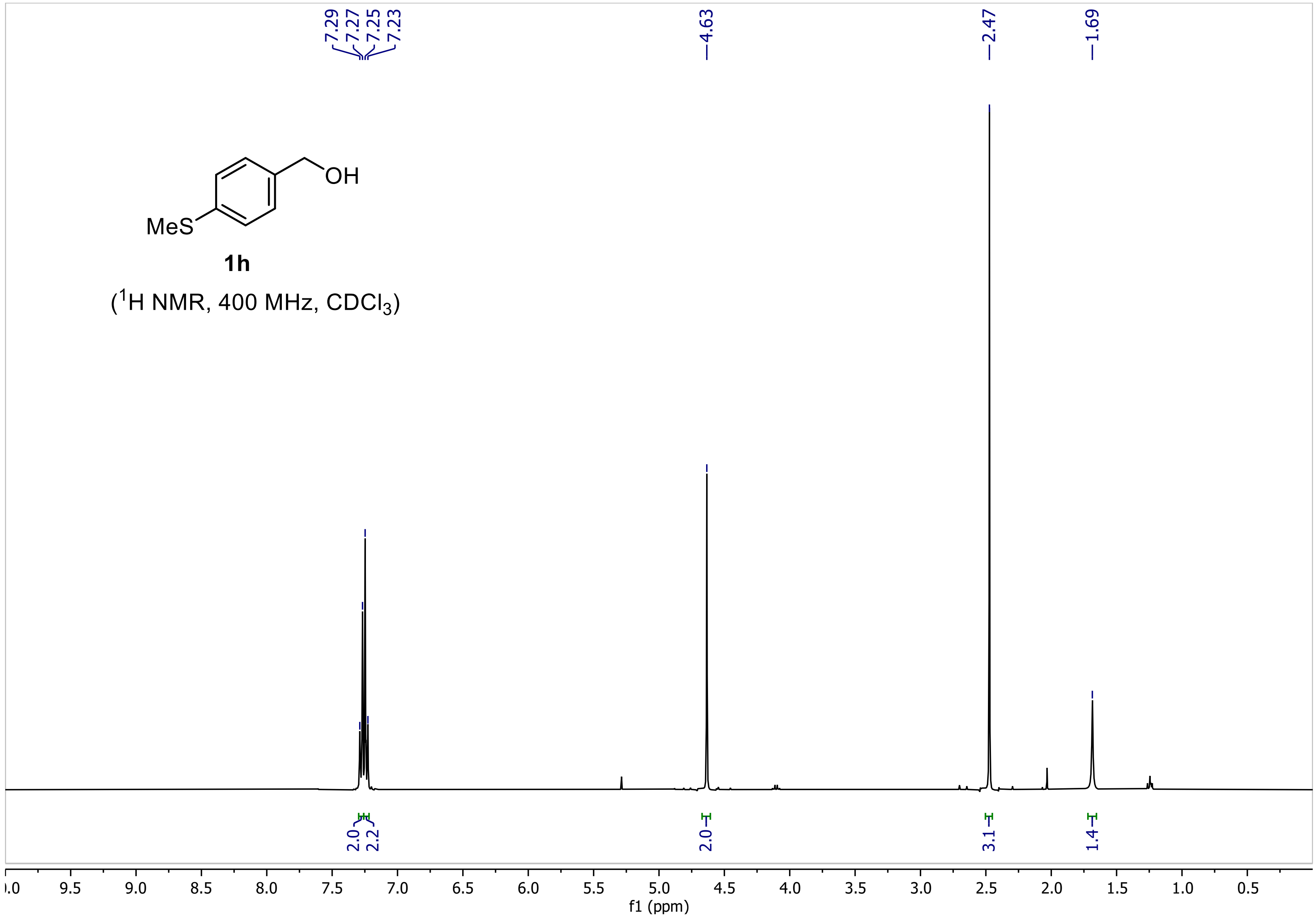

1h

(^{1}H NMR, 400 MHz, $CDCl_3$)

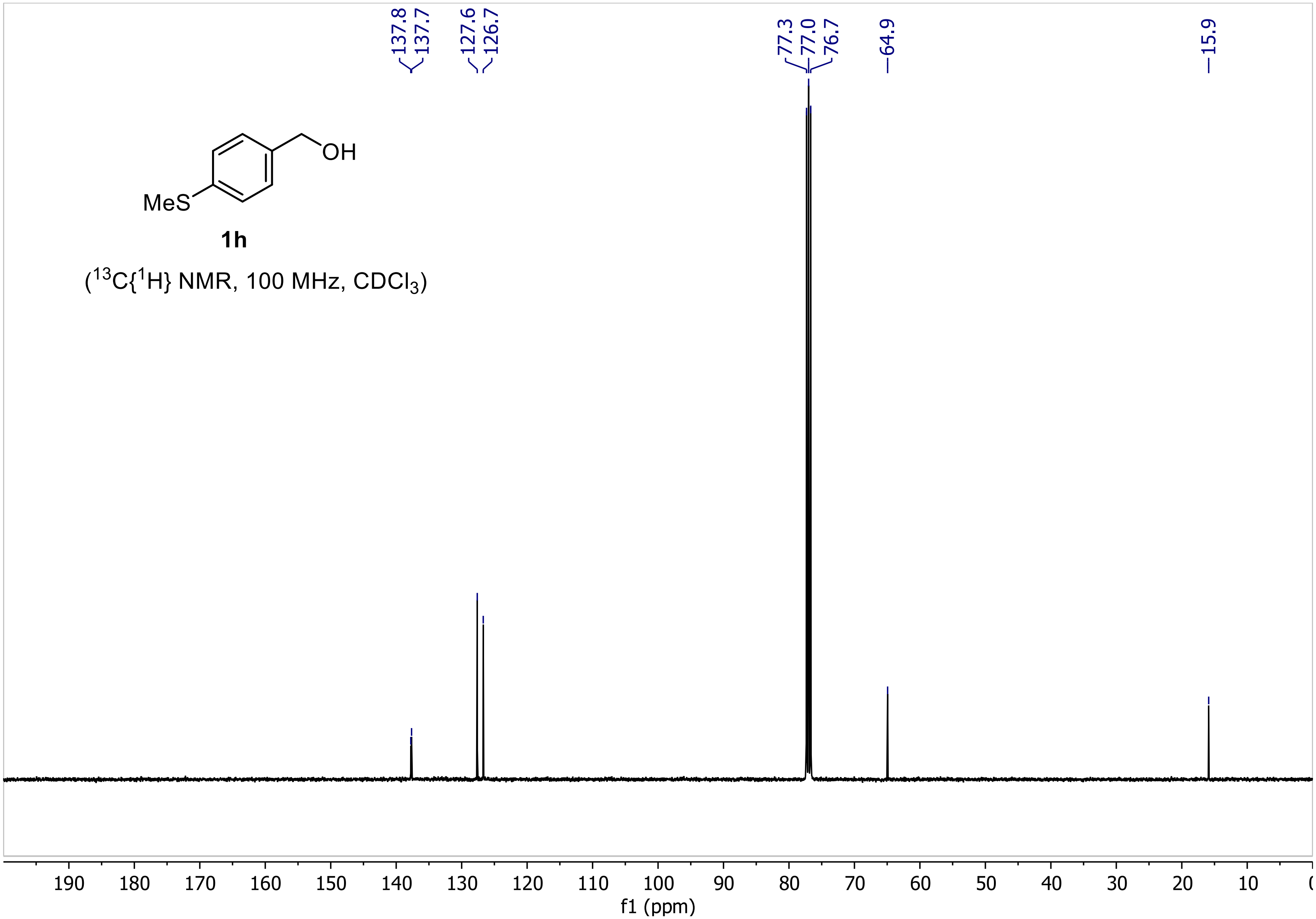

1h

($^{13}C\{^{1}H\}$ NMR, 100 MHz, $CDCl_3$)

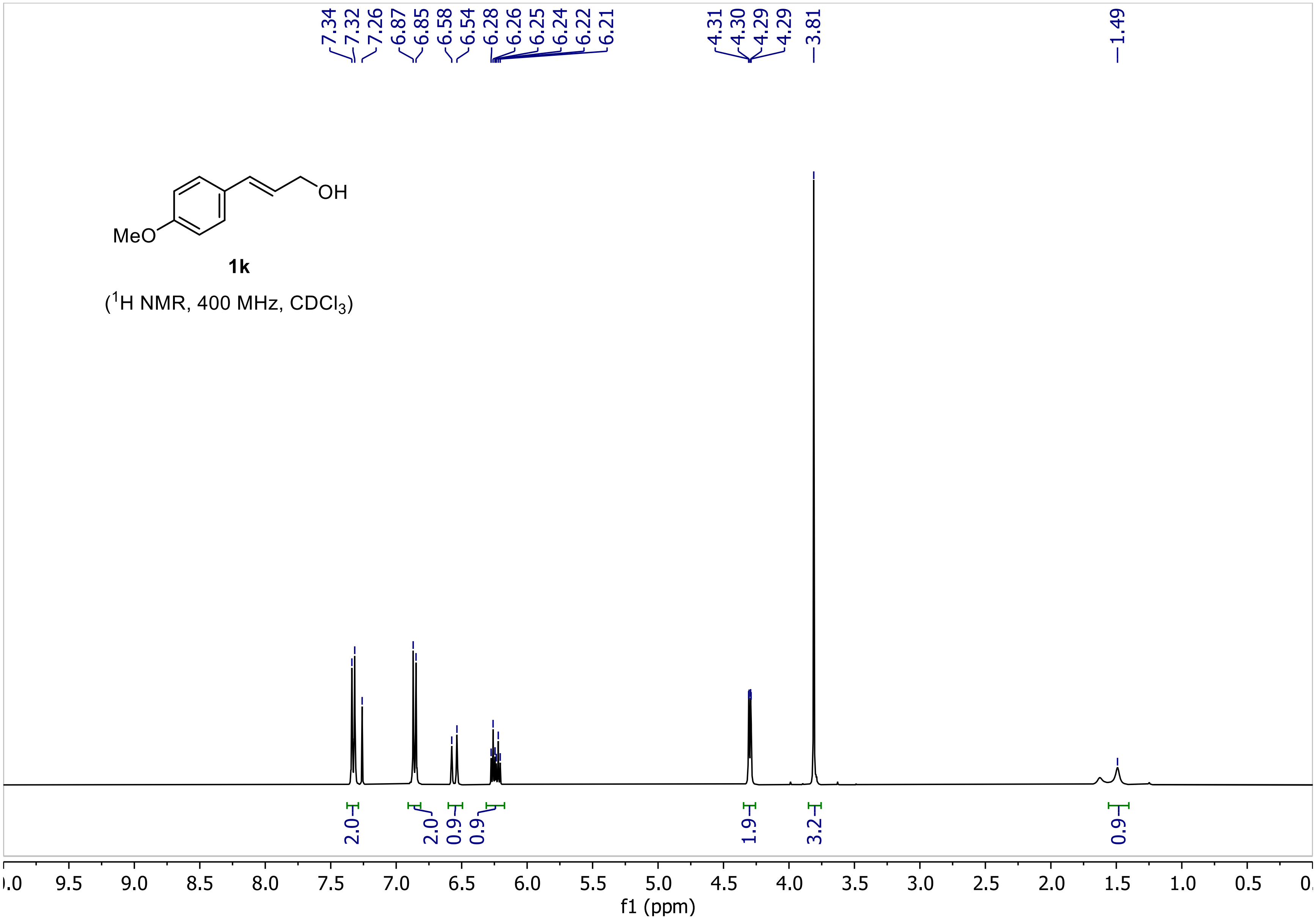

7.34
7.32
7.26
6.87
6.85
6.58
6.54
6.28
6.26
6.25
6.24
6.22
6.21
4.31
4.30
4.29
4.29
3.81
1.49
OH
MeO
1k
(^{1}H NMR, 400 MHz, CDCl$_3$)
2.0
2.0
0.9
0.9
1.9
3.2
0.9
10.0
9.5
9.0
8.5
8.0
7.5
7.0
6.5
6.0
5.5
5.0
4.5
4.0
3.5
3.0
2.5
2.0
1.5
1.0
0.5
0
f1 (ppm)

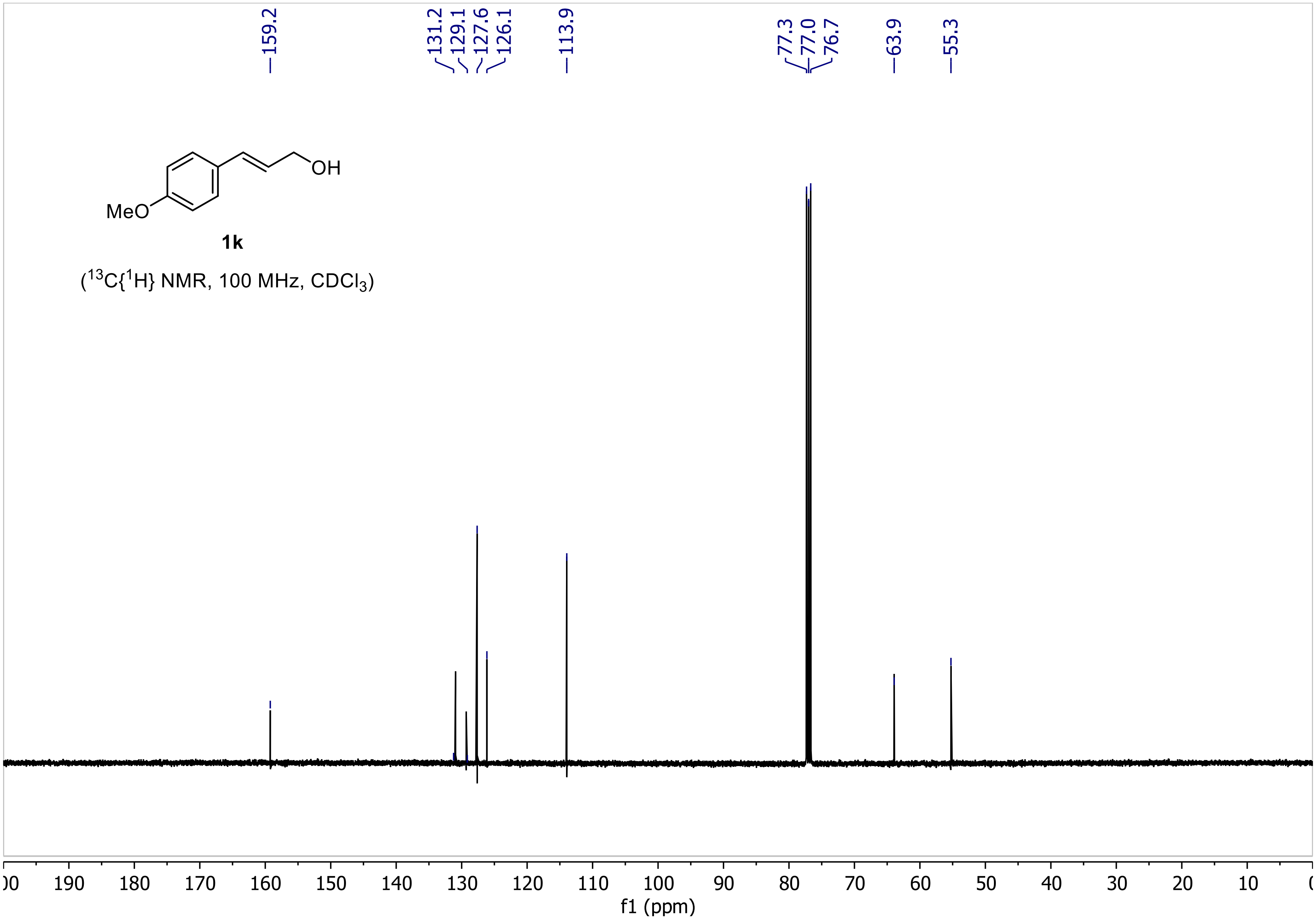

1k

($^{13}C\{^{1}H\}$ NMR, 100 MHz, $CDCl_3$)

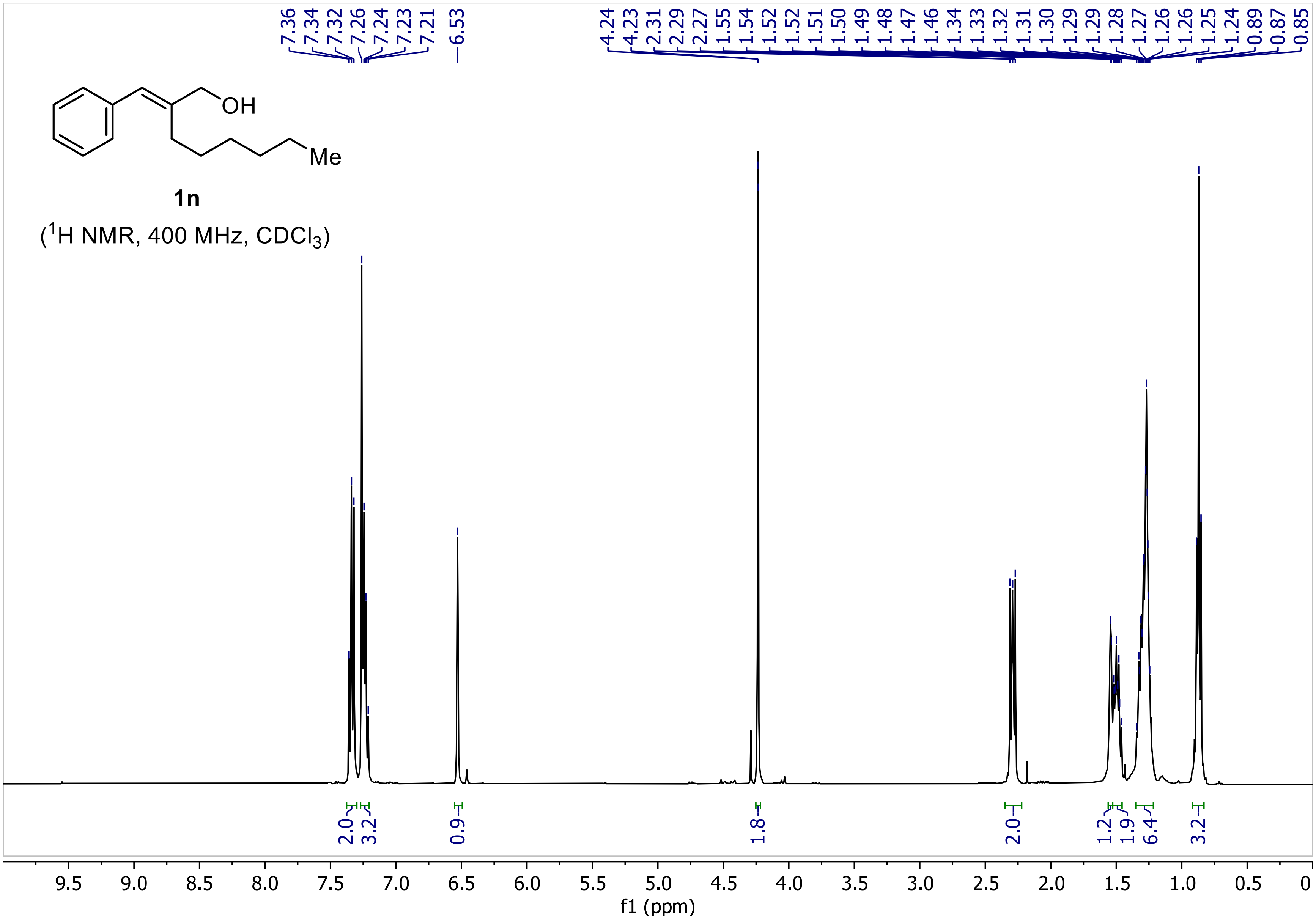
7.36
7.34
7.32
7.26
7.24
7.23
7.21
6.53
4.24
4.23
2.31
2.29
2.27
1.55
1.54
1.52
1.52
1.51
1.50
1.49
1.48
1.47
1.46
1.34
1.33
1.32
1.31
1.30
1.29
1.29
1.28
1.27
1.26
1.26
1.25
1.24
0.89
0.87
0.85
OH
Me
1n
(1H NMR, 400 MHz, CDCl3)
2.0
3.2
0.9
1.8
2.0
1.2
1.9
6.4
3.2
9.5
9.0
8.5
8.0
7.5
7.0
6.5
6.0
5.5
5.0
4.5
4.0
3.5
3.0
2.5
2.0
1.5
1.0
0.5
f1 (ppm)

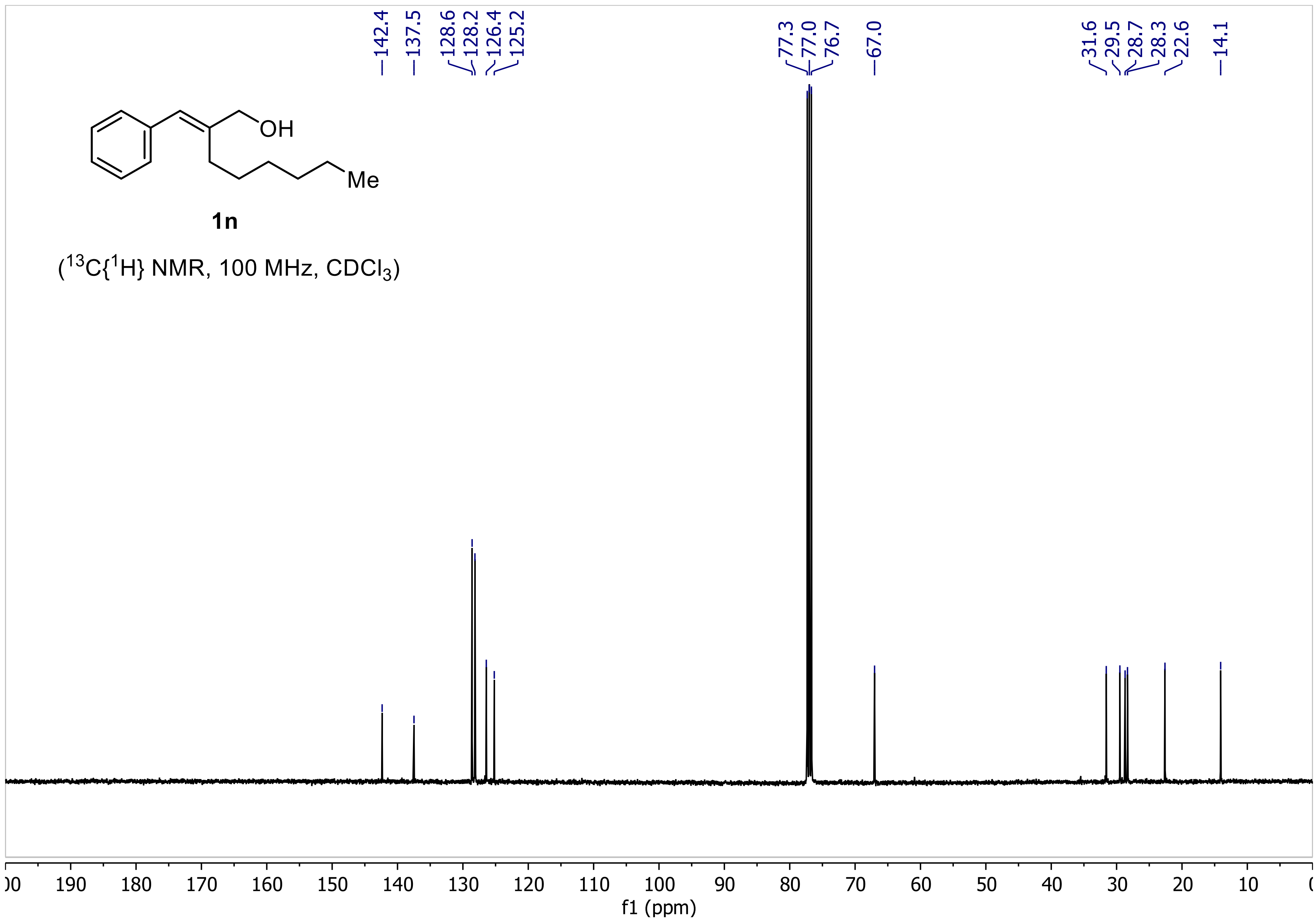

142.4
137.5
128.6
128.2
126.4
125.2
77.3
77.0
76.7
67.0
31.6
29.5
28.7
28.3
22.6
14.1
OH
Me
1n
(13C{1H} NMR, 100 MHz, CDCl3)
f1 (ppm)

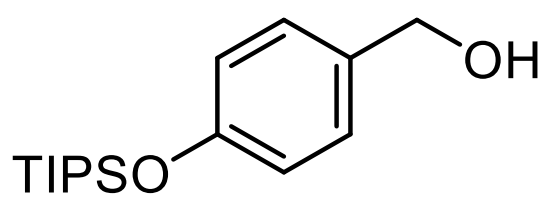

1e

(^{1}H NMR, 400 MHz, $CDCl_3$)

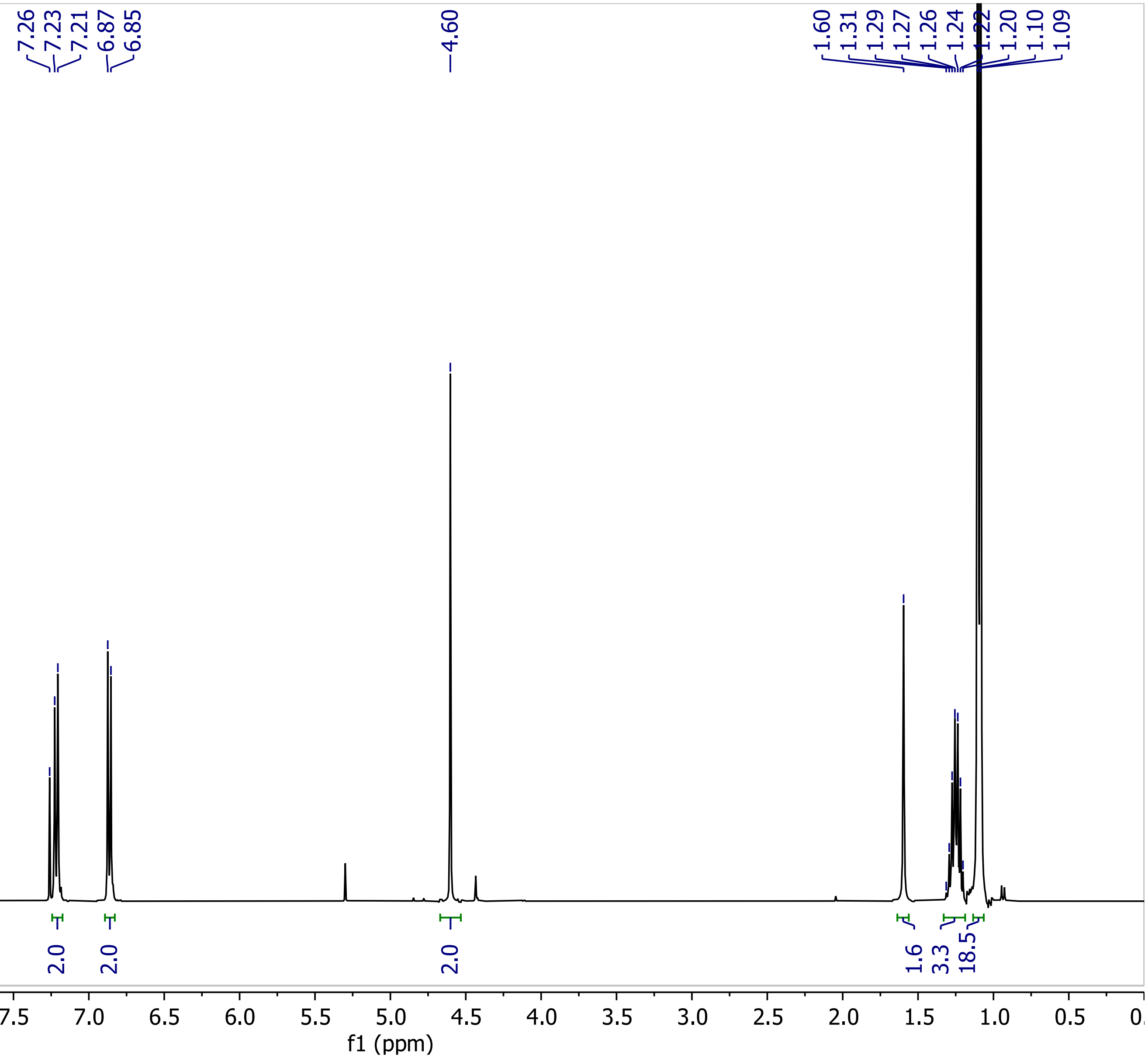

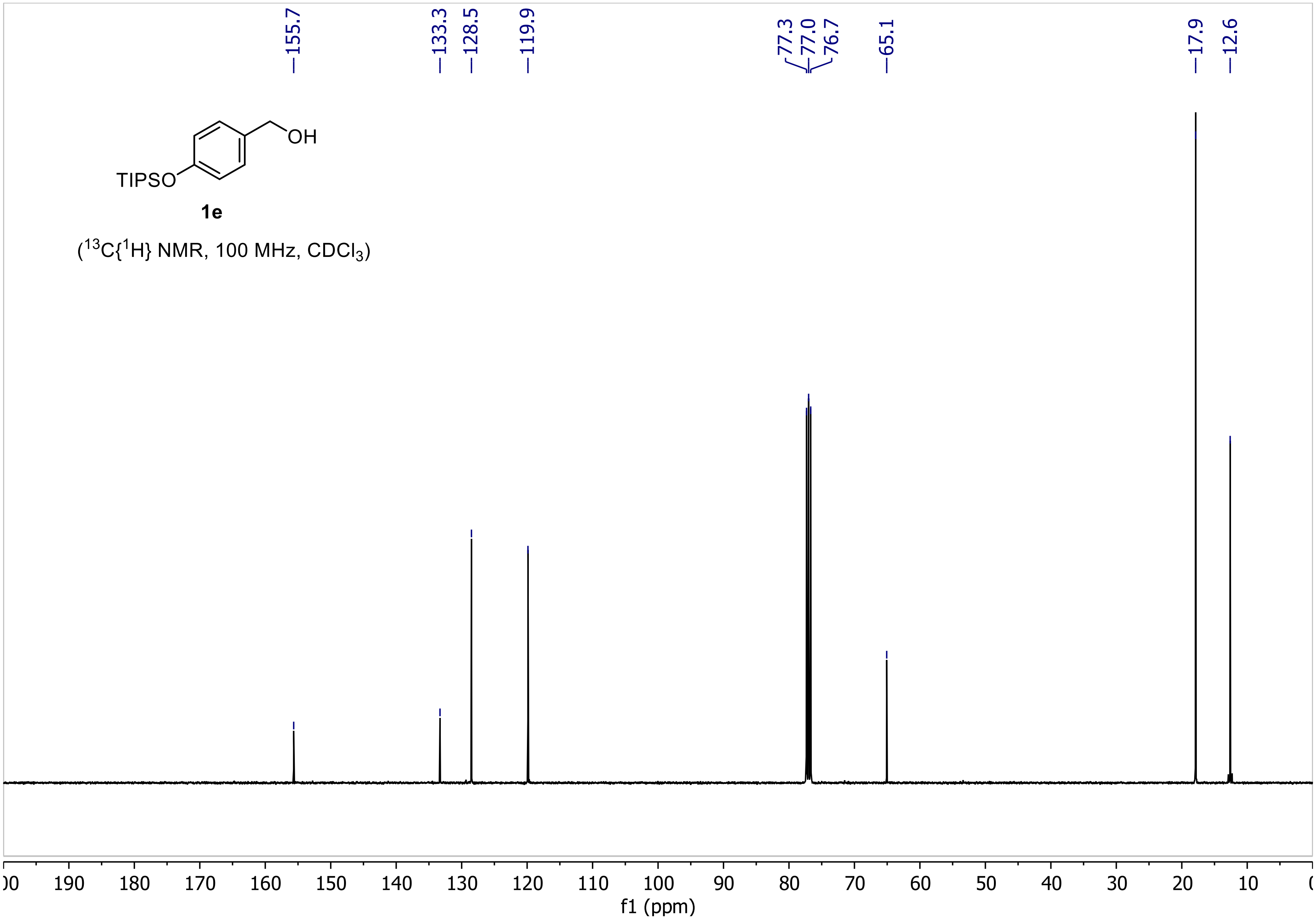
155.7
133.3
128.5
119.9
77.3
77.0
76.7
65.1
17.9
12.6
OH
TIPSO
1e
(^{13}C{^{1}H} NMR, 100 MHz, $CDCl_3$)
190
180
170
160
150
140
130
120
110
100
90
80
70
60
50
40
30
20
10
f1 (ppm)

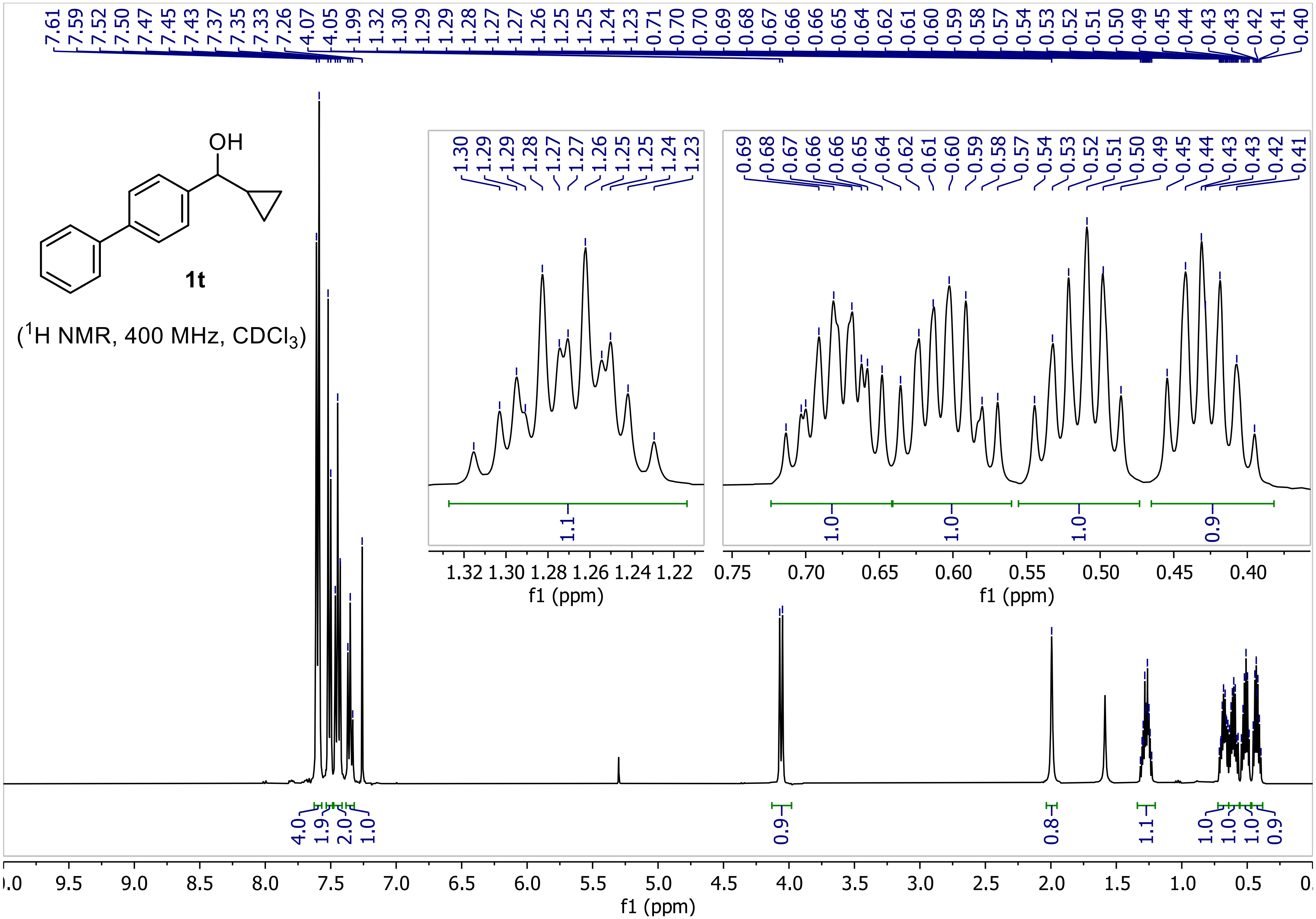
OH
1t
(1H NMR, 400 MHz, CDCl3)
f1 (ppm)

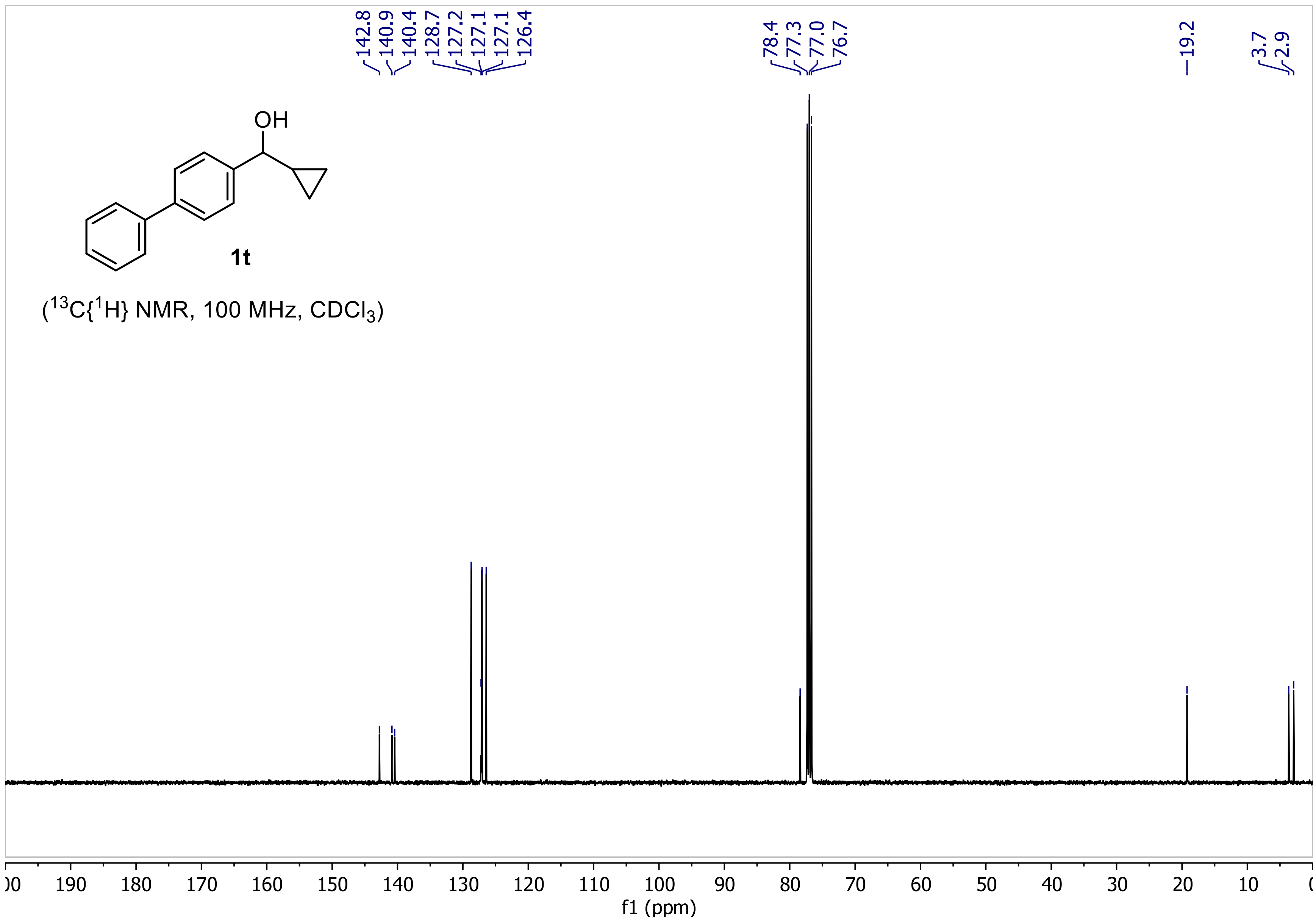

OH
1t
(13C{1H} NMR, 100 MHz, CDCl3)
142.8
140.9
140.4
128.7
127.2
127.1
127.1
126.4
78.4
77.3
77.0
76.7
19.2
3.7
2.9
f1 (ppm)

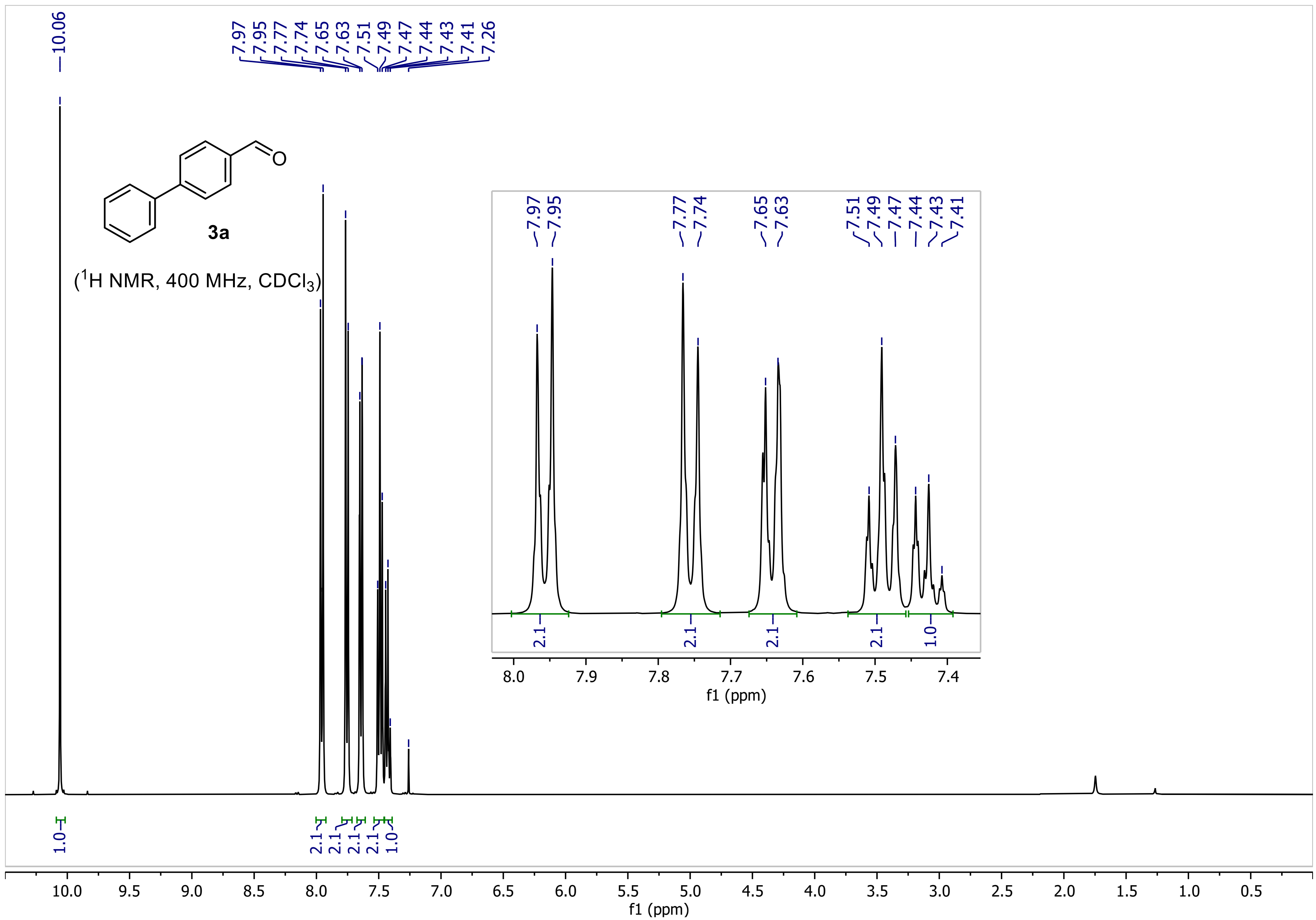
10.06
7.97
7.95
7.77
7.74
7.65
7.63
7.51
7.49
7.47
7.44
7.43
7.41
7.26
3a
(¹H NMR, 400 MHz, CDCl₃)
1.0
2.1
2.1
2.1
2.1
1.0
f1 (ppm)

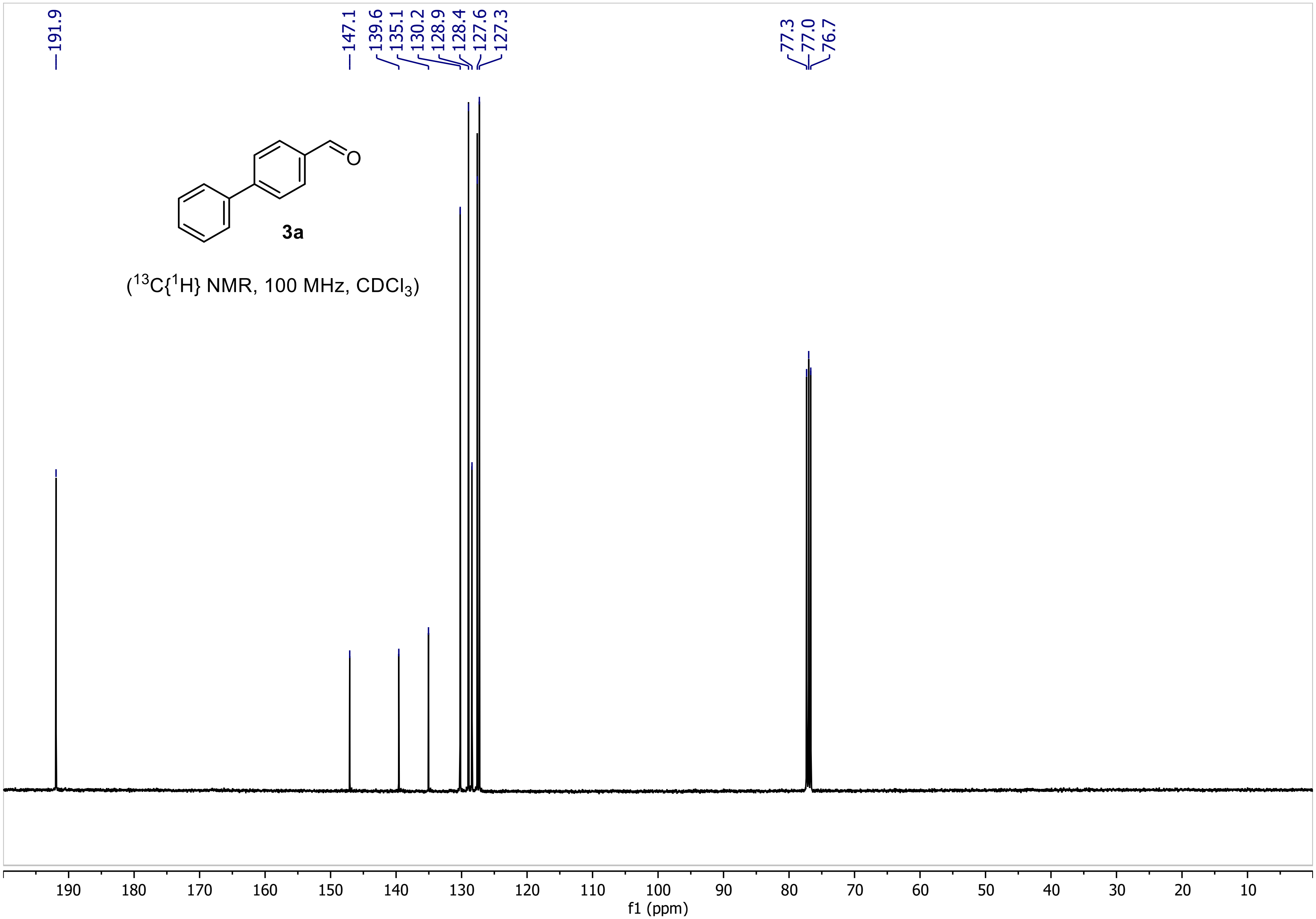
191.9
147.1
139.6
135.1
130.2
128.9
128.4
127.6
127.3
77.3
77.0
76.7
O
3a
(13C{1H} NMR, 100 MHz, CDCl3)
190
180
170
160
150
140
130
120
110
100
90
80
70
60
50
40
30
20
10
f1 (ppm)

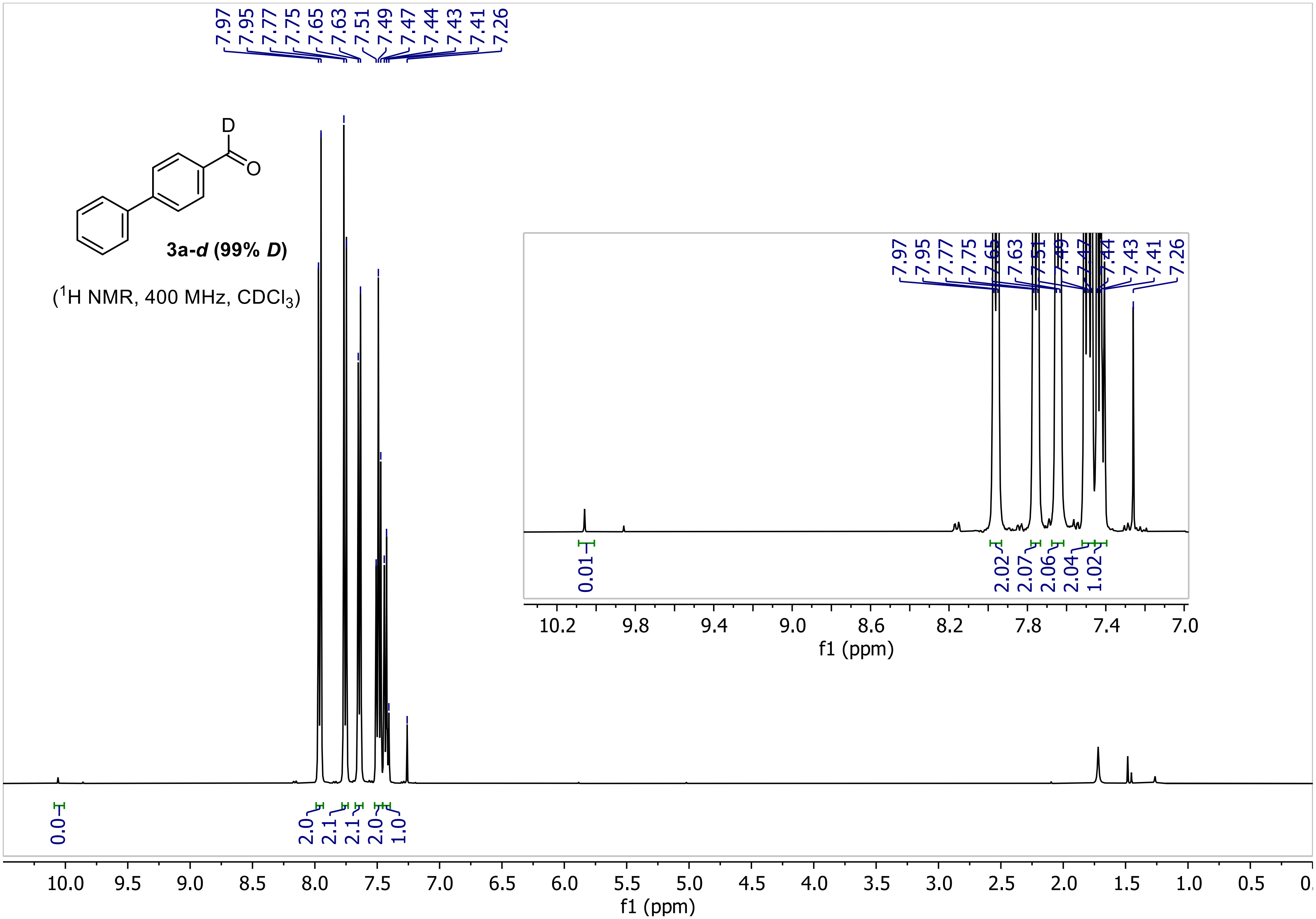

D
O
3a-*d* (99% *D*)
(^{1}H NMR, 400 MHz, $CDCl_3$)
7.97 7.95 7.77 7.75 7.65 7.63 7.51 7.49 7.47 7.44 7.43 7.41 7.26
0.01 2.02 2.07 2.06 2.04 1.02
10.2 9.8 9.4 9.0 8.6 8.2 7.8 7.4 7.0
f1 (ppm)
0.0 2.0 2.1 2.1 2.0 1.0
10.0 9.5 9.0 8.5 8.0 7.5 7.0 6.5 6.0 5.5 5.0 4.5 4.0 3.5 3.0 2.5 2.0 1.5 1.0 0.5 0
f1 (ppm)

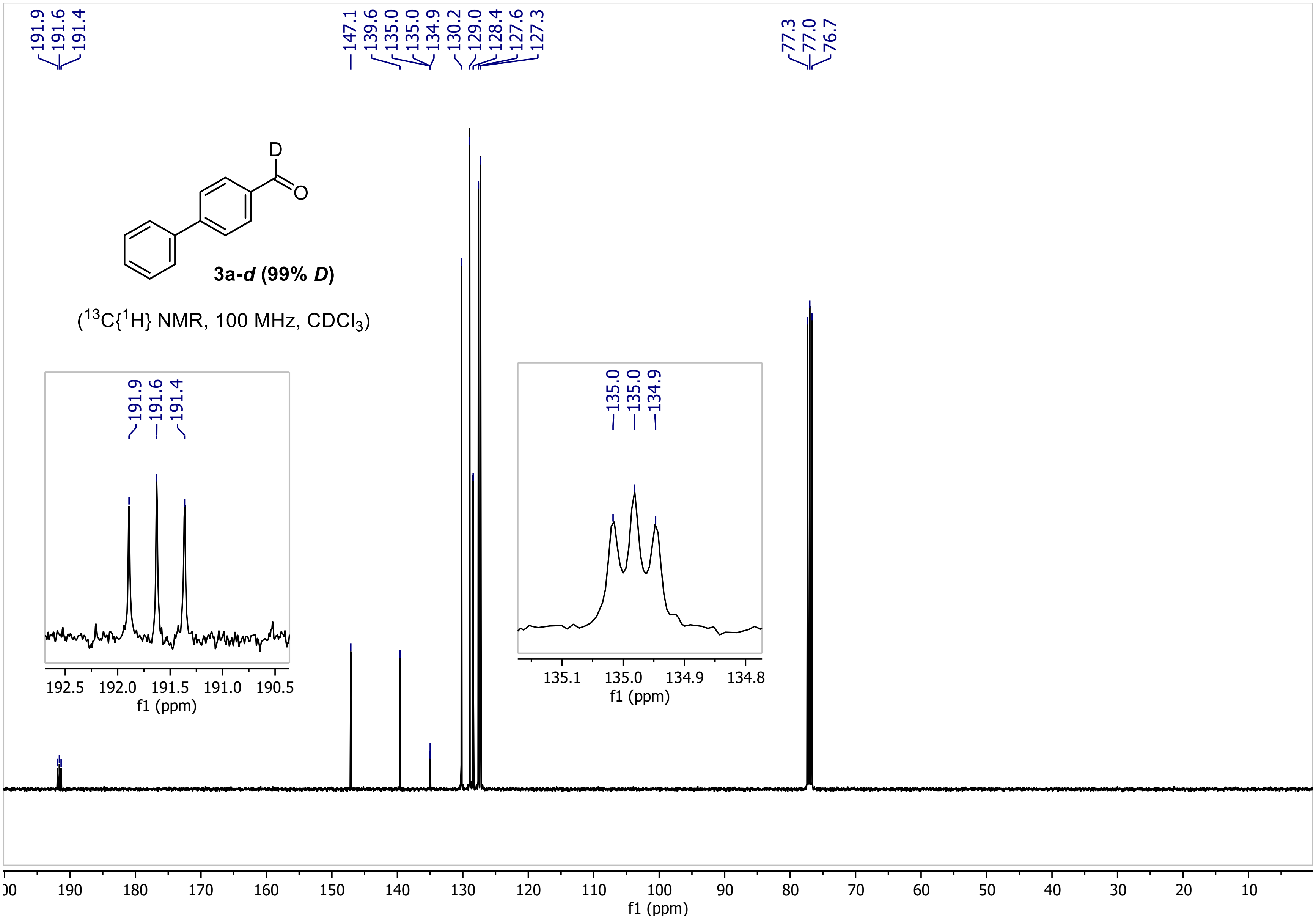
191.9
191.6
191.4
147.1
139.6
135.0
135.0
134.9
130.2
129.0
128.4
127.6
127.3
77.3
77.0
76.7
D
O
3a-*d* (99% *D*)
(^{13}C{^{1}H} NMR, 100 MHz, CDCl$_3$)
192.5 192.0 191.5 191.0 190.5
f1 (ppm)
135.1 135.0 134.9 134.8
f1 (ppm)
190 180 170 160 150 140 130 120 110 100 90 80 70 60 50 40 30 20 10
f1 (ppm)

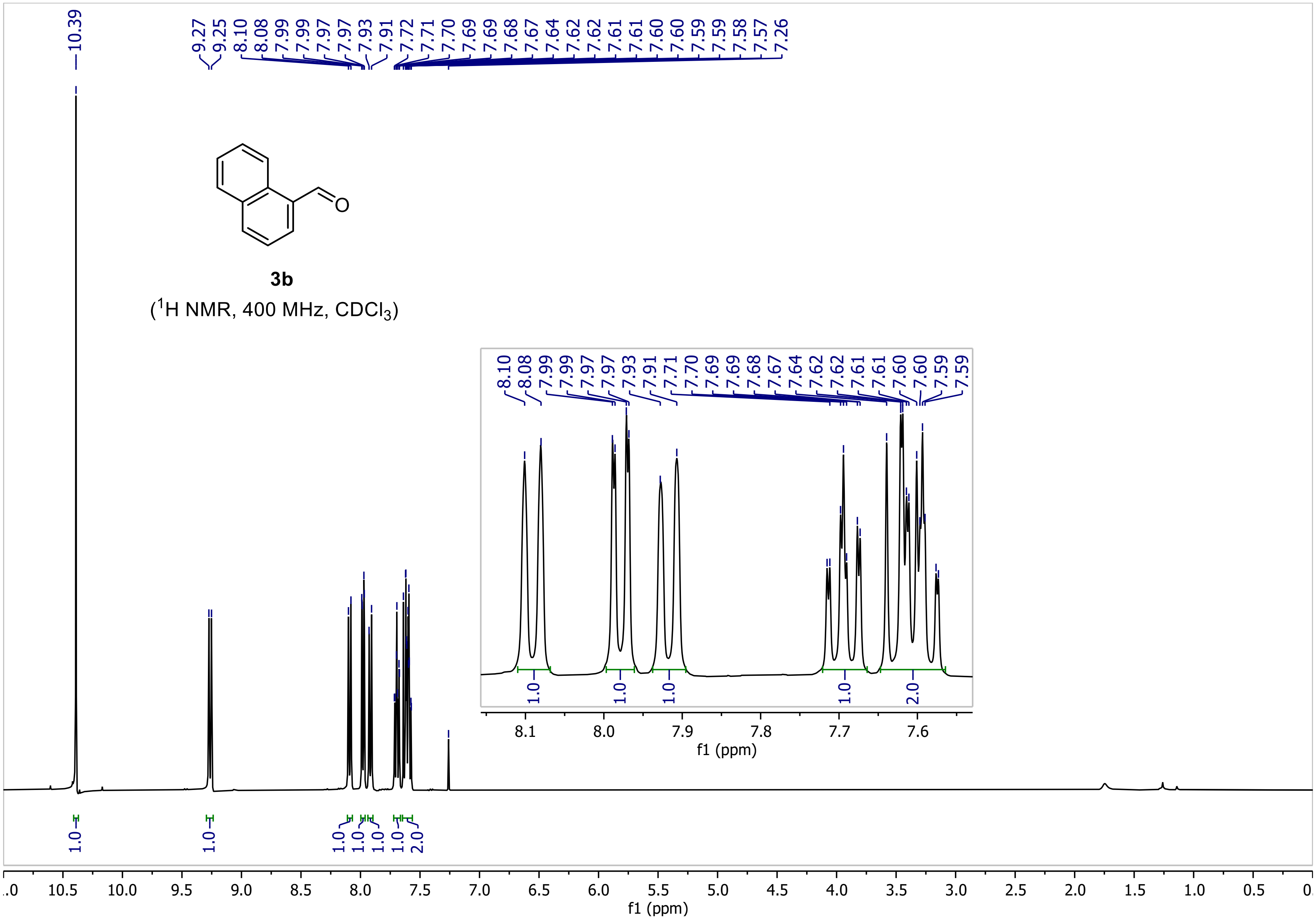
10.39
9.27
9.25
8.10
8.08
7.99
7.99
7.97
7.97
7.93
7.91
7.72
7.71
7.70
7.69
7.69
7.68
7.67
7.64
7.62
7.62
7.61
7.61
7.60
7.60
7.59
7.59
7.58
7.57
7.26
3b
(^{1}H NMR, 400 MHz, $CDCl_3$)
1.0
1.0
1.0
1.0
1.0
1.0
2.0
f1 (ppm)

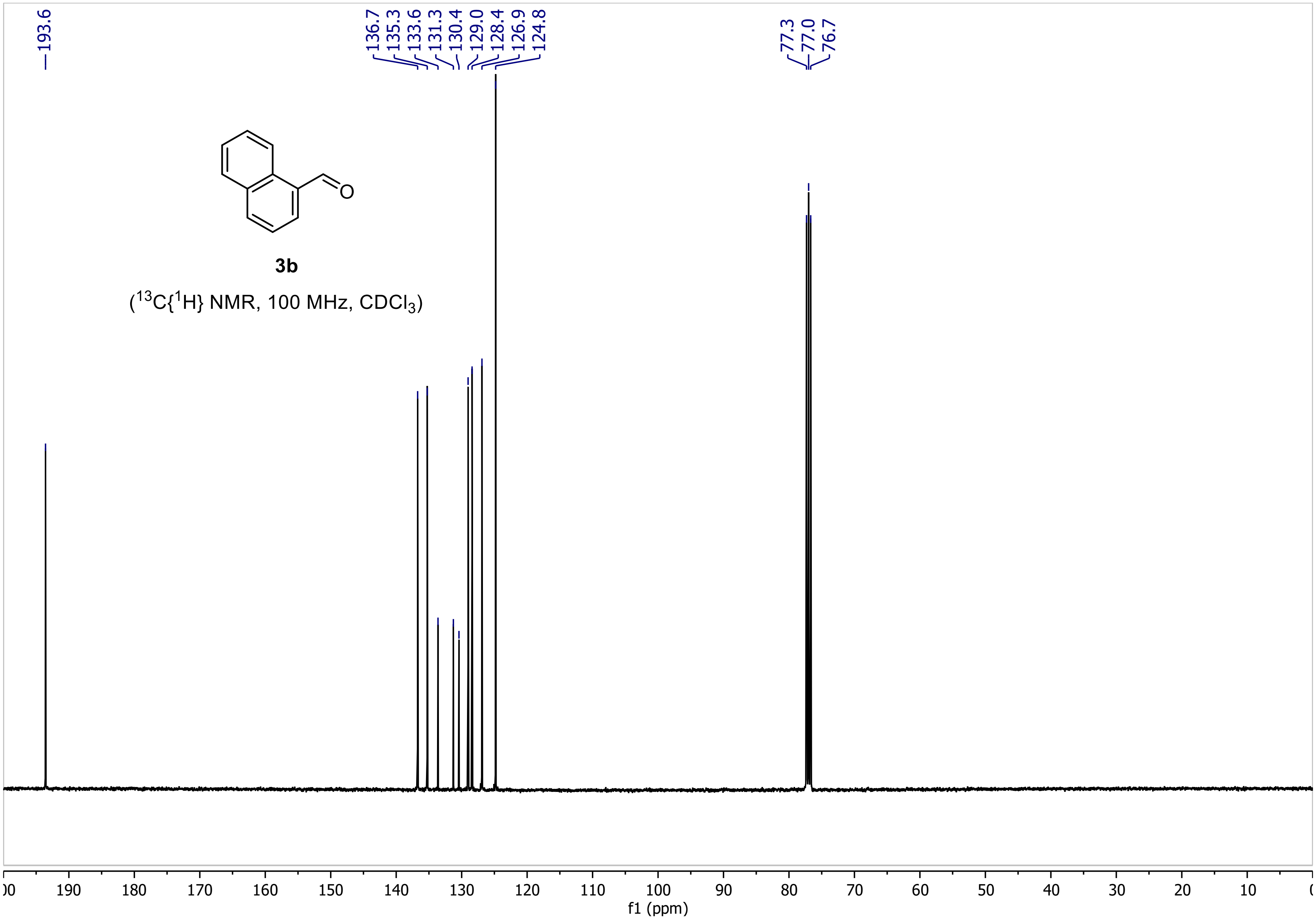

3b

($^{13}C\{^{1}H\}$ NMR, 100 MHz, $CDCl_3$)

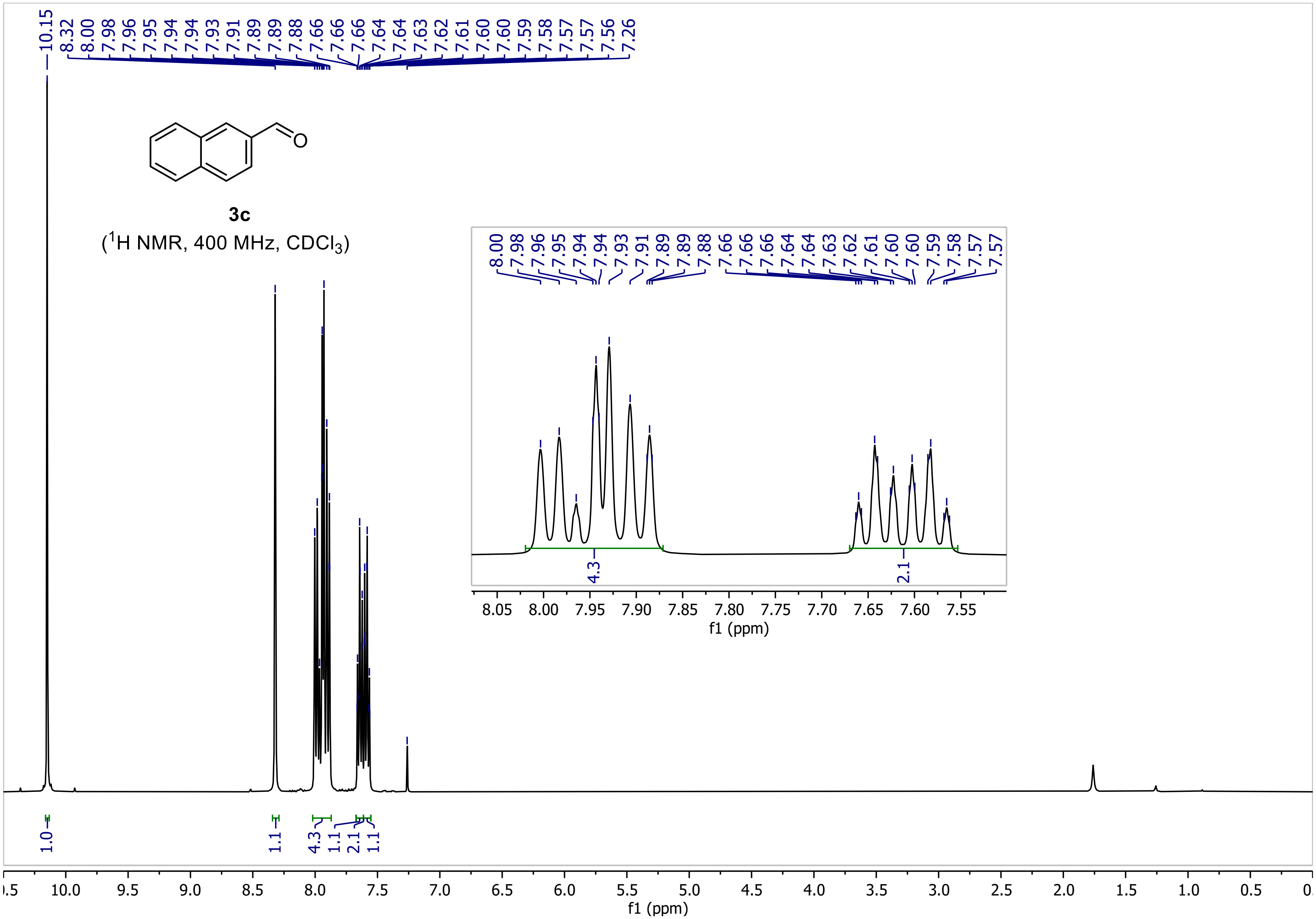
3c
(1H NMR, 400 MHz, CDCl3)
f1 (ppm)

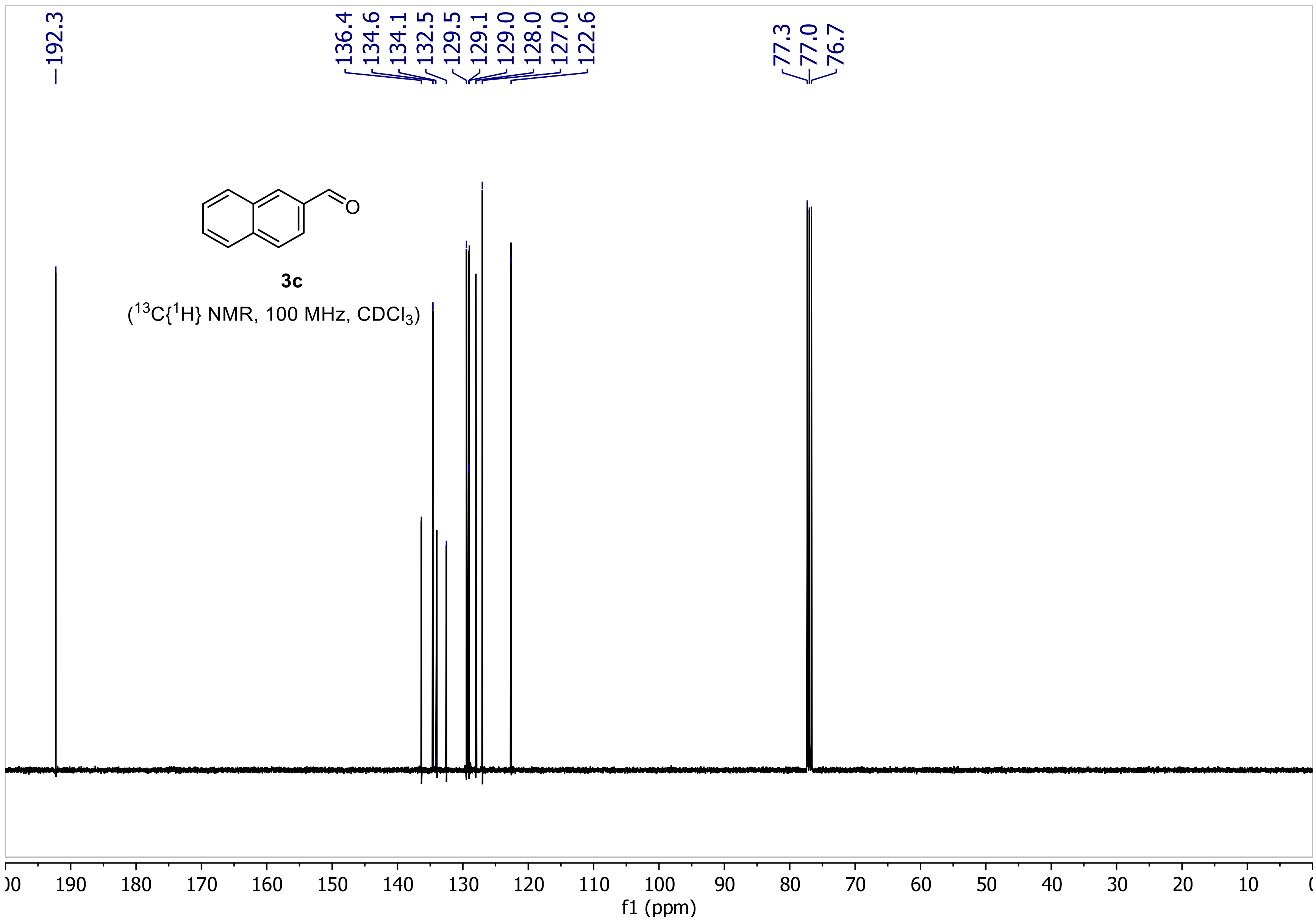
192.3
136.4
134.6
134.1
132.5
129.5
129.1
129.0
128.0
127.0
122.6
77.3
77.0
76.7
O
3c
($^{13}C\{^{1}H\}$ NMR, 100 MHz, $CDCl_3$)
190
180
170
160
150
140
130
120
110
100
90
80
70
60
50
40
30
20
10
f1 (ppm)

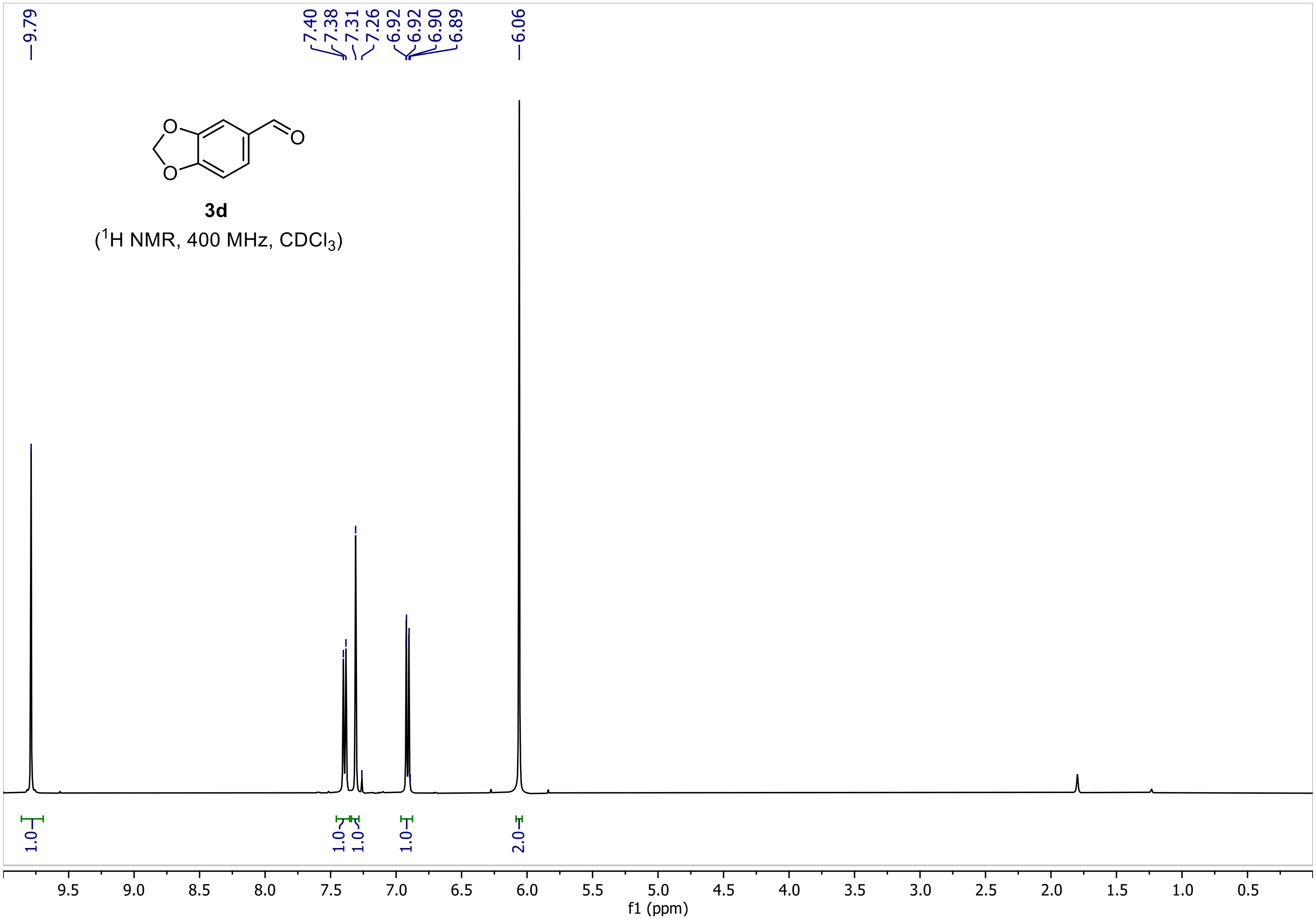

3d
(1H NMR, 400 MHz, CDCl3)
9.79
7.40
7.38
7.31
7.26
6.92
6.92
6.90
6.89
6.06
1.0
1.0
1.0
1.0
2.0
9.5
9.0
8.5
8.0
7.5
7.0
6.5
6.0
5.5
5.0
4.5
4.0
3.5
3.0
2.5
2.0
1.5
1.0
0.5
f1 (ppm)

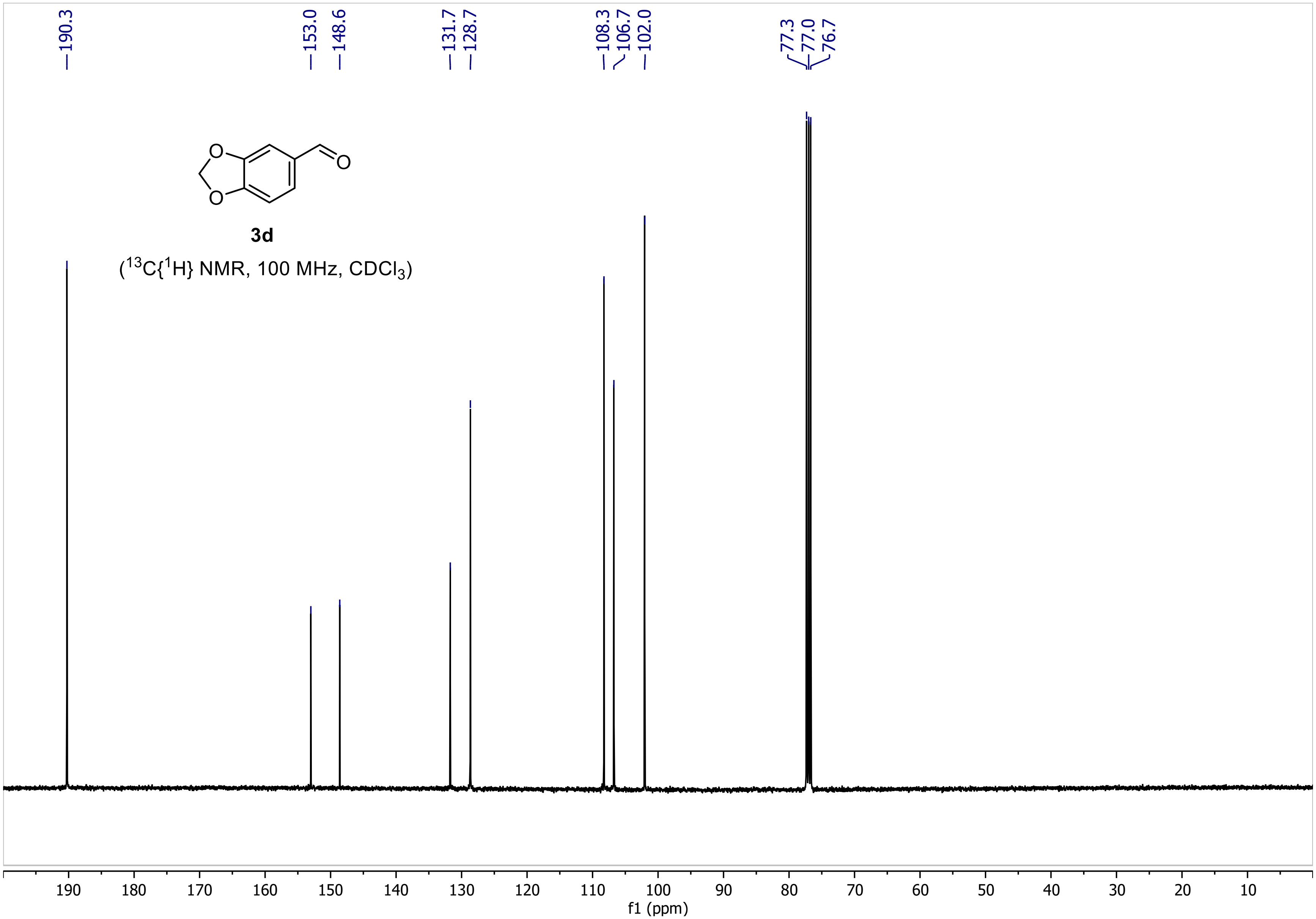
190.3
153.0
148.6
131.7
128.7
108.3
106.7
102.0
77.3
77.0
76.7
3d
(^{13}C{^{1}H} NMR, 100 MHz, CDCl$_3$)
190
180
170
160
150
140
130
120
110
100
90
80
70
60
50
40
30
20
10
f1 (ppm)

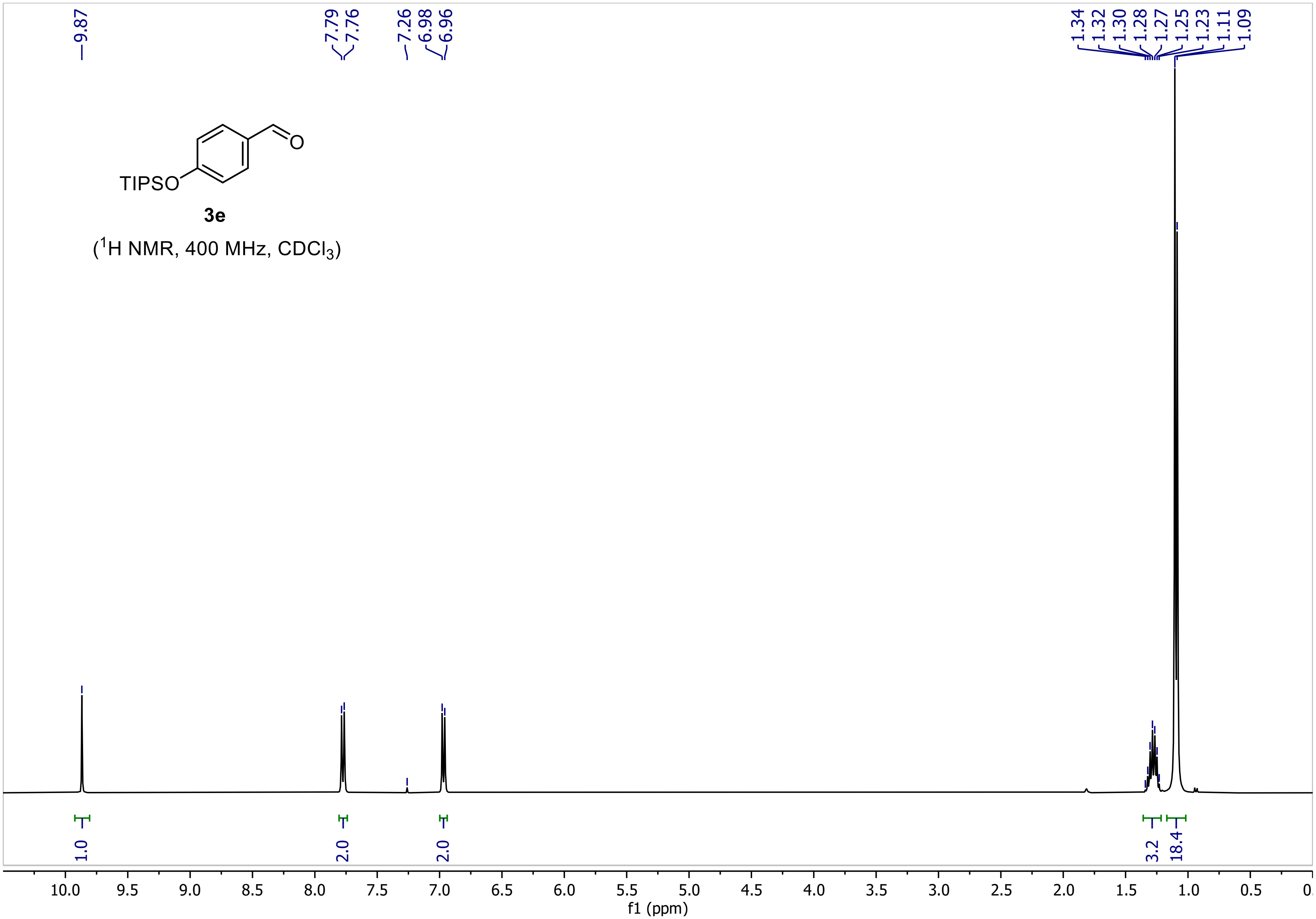

3e

(^{1}H NMR, 400 MHz, $CDCl_3$)

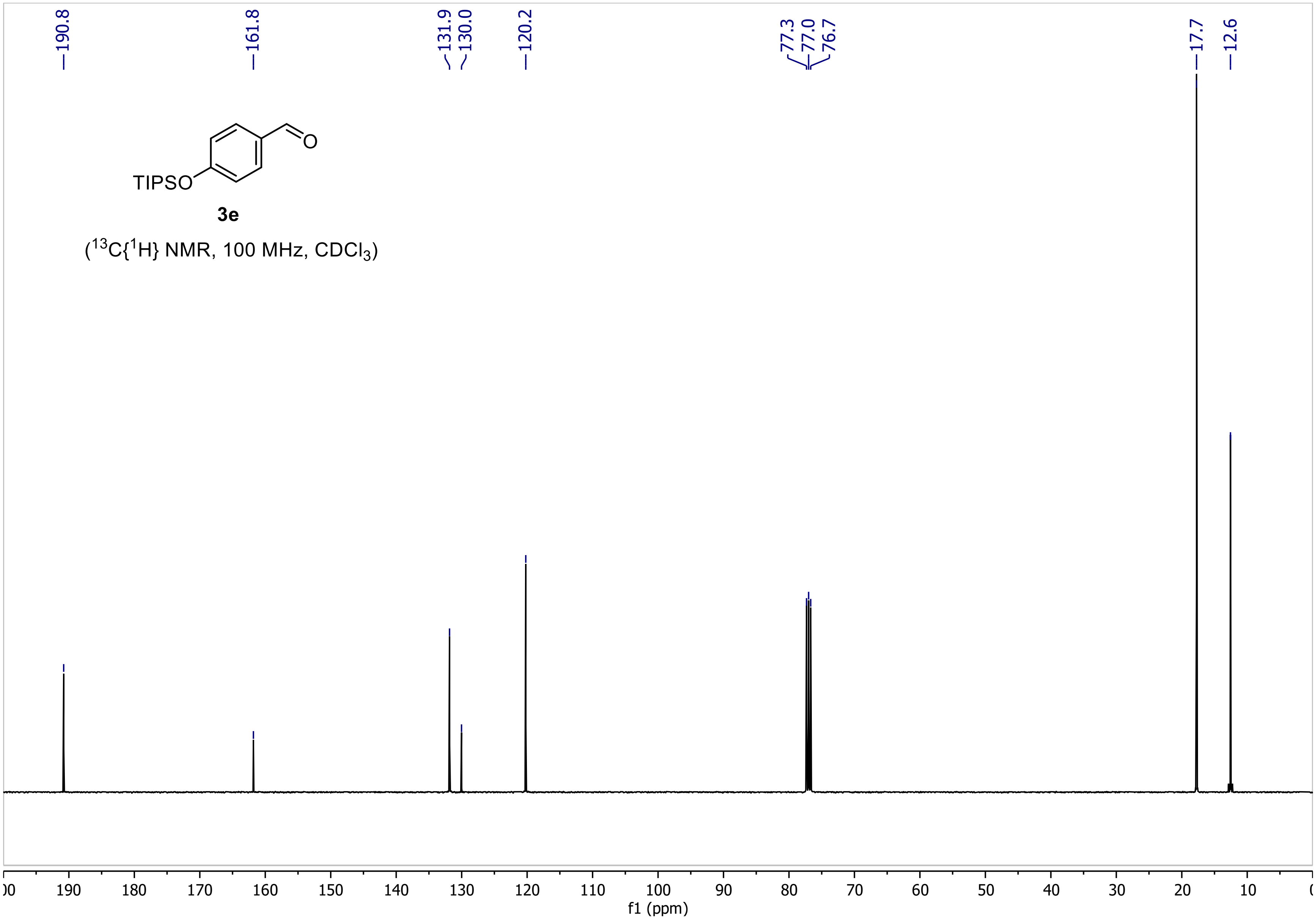
TIPSO
O
3e
($^{13}C\{^{1}H\}$ NMR, 100 MHz, $CDCl_3$)
190.8
161.8
131.9
130.0
120.2
77.3
77.0
76.7
17.7
12.6
f1 (ppm)

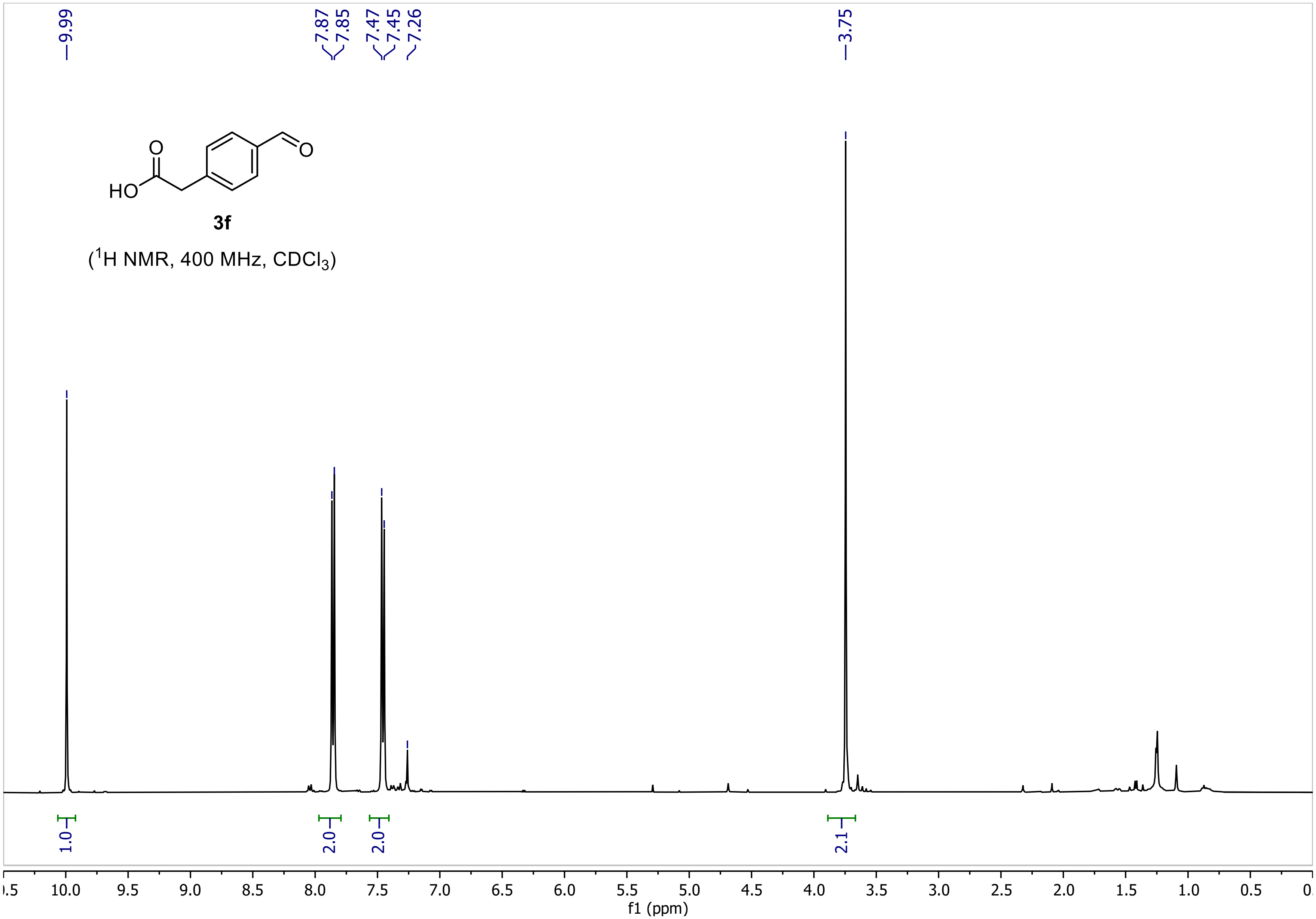
9.99
7.87
7.85
7.47
7.45
7.26
3.75
HO
O
O
3f
(1H NMR, 400 MHz, CDCl3)
1.0
2.0
2.0
2.1
f1 (ppm)

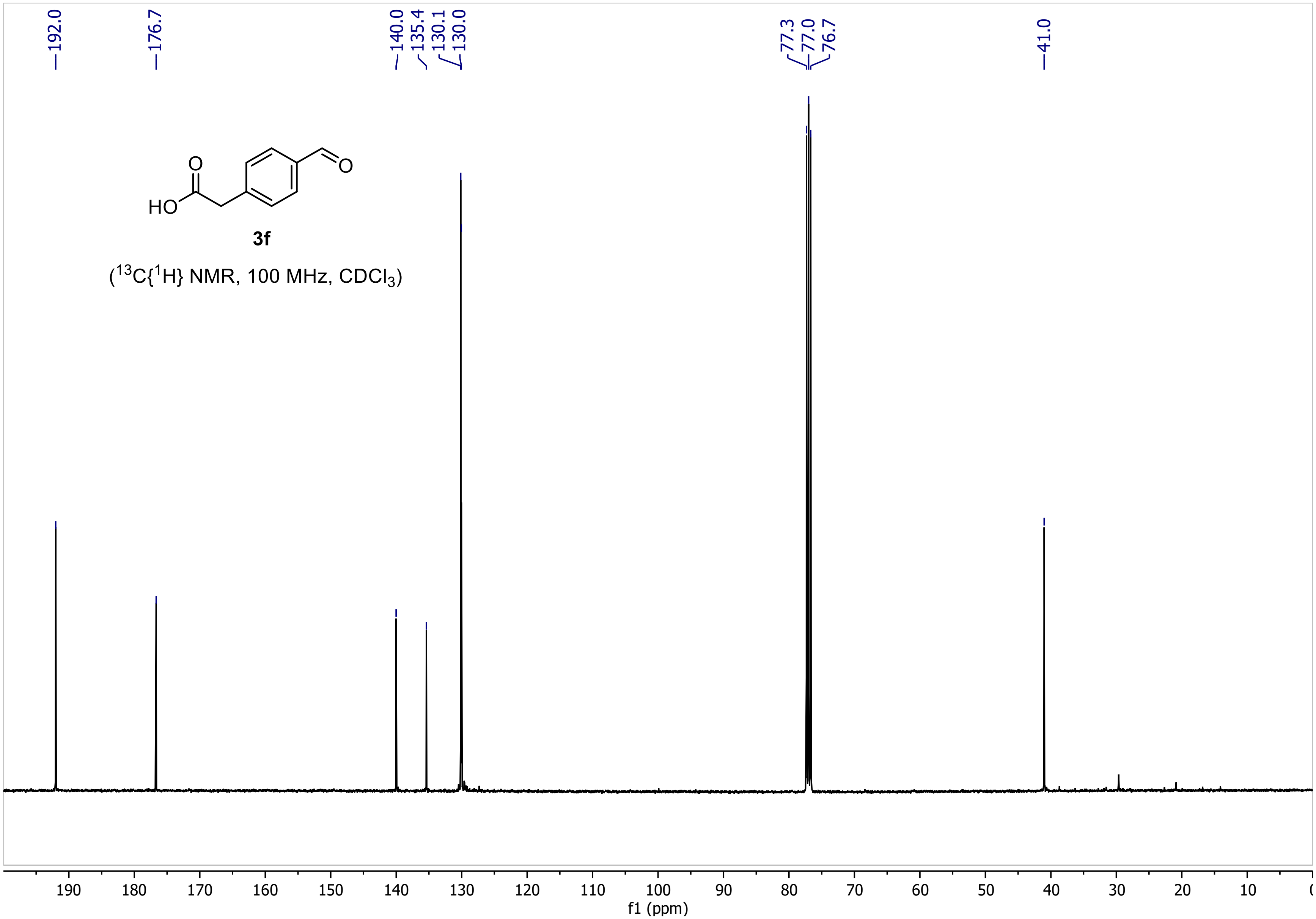

192.0
176.7
140.0
135.4
130.1
130.0
77.3
77.0
76.7
41.0
HO
O
O
3f
(13C{1H} NMR, 100 MHz, CDCl3)
190
180
170
160
150
140
130
120
110
100
90
80
70
60
50
40
30
20
10
f1 (ppm)

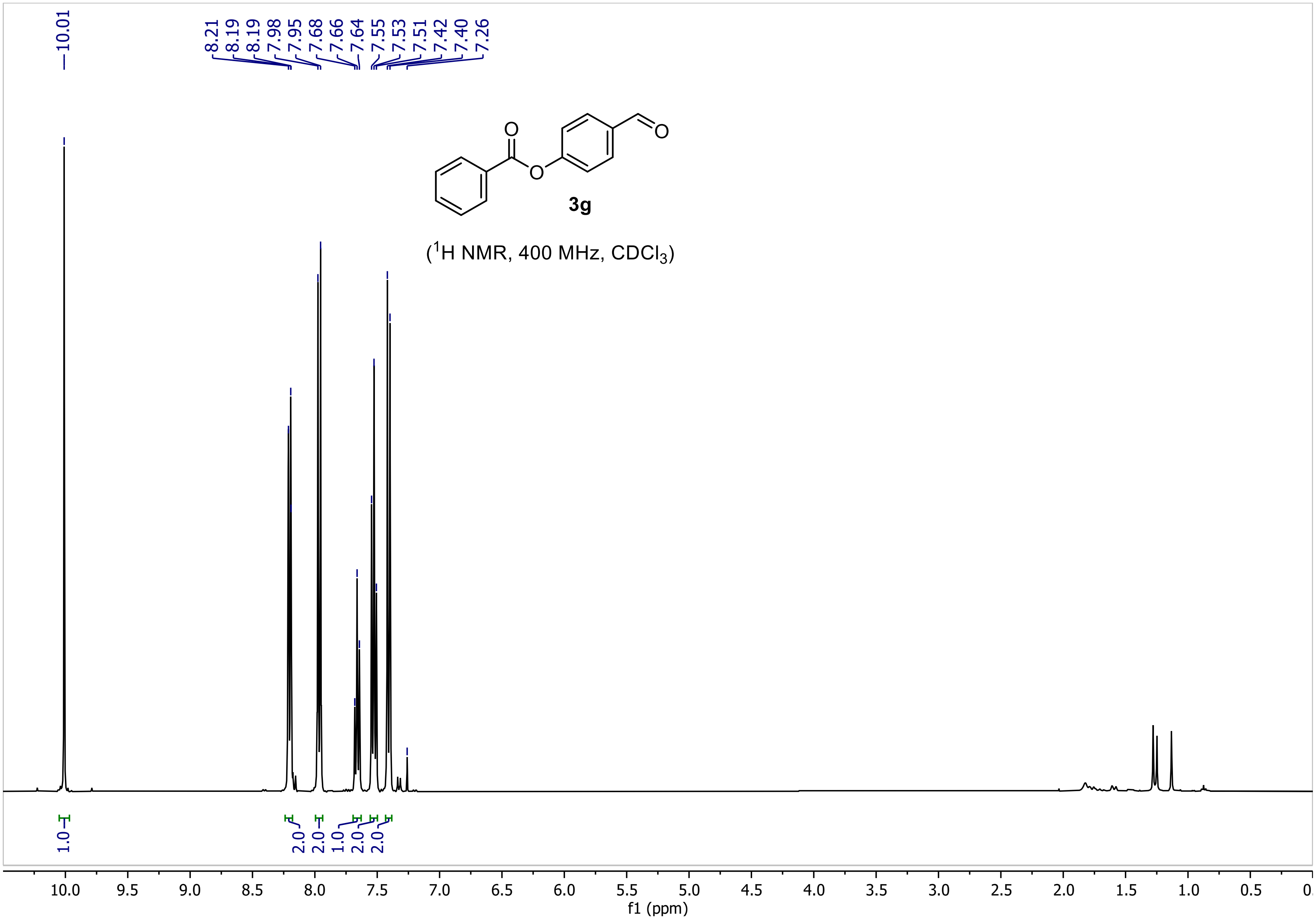
10.01
8.21
8.19
8.19
7.98
7.95
7.68
7.66
7.64
7.55
7.53
7.51
7.42
7.40
7.26
O
O
O
3g
(1H NMR, 400 MHz, CDCl3)
1.0
2.0
2.0
1.0
2.0
2.0
10.0
9.5
9.0
8.5
8.0
7.5
7.0
6.5
6.0
5.5
5.0
4.5
4.0
3.5
3.0
2.5
2.0
1.5
1.0
0.5
f1 (ppm)

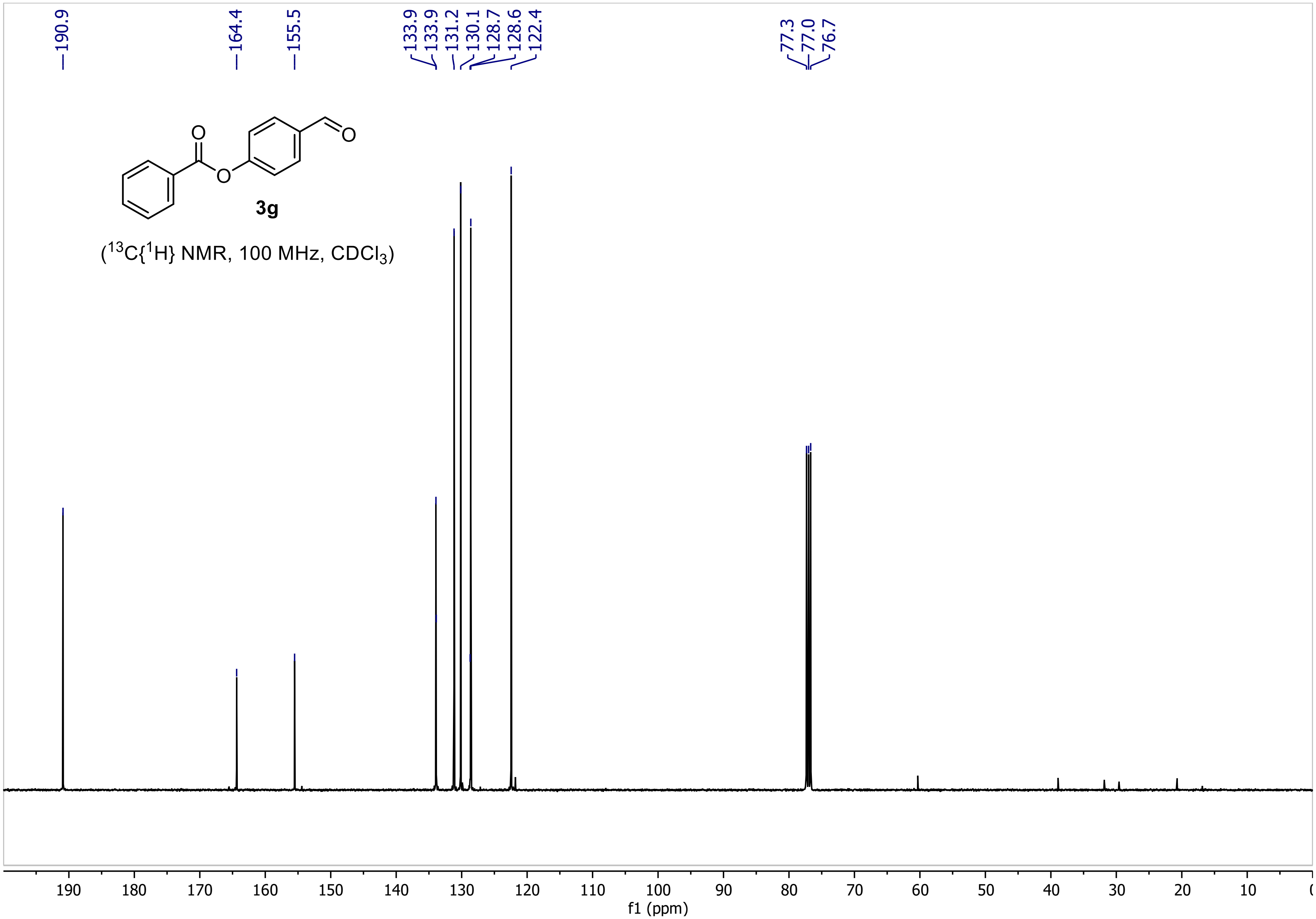
190.9
164.4
155.5
133.9
133.9
131.2
130.1
128.7
128.6
122.4
77.3
77.0
76.7
O
O
O
3g
(^{13}C{^{1}H} NMR, 100 MHz, $CDCl_3$)
190
180
170
160
150
140
130
120
110
100
90
80
70
60
50
40
30
20
10
f1 (ppm)

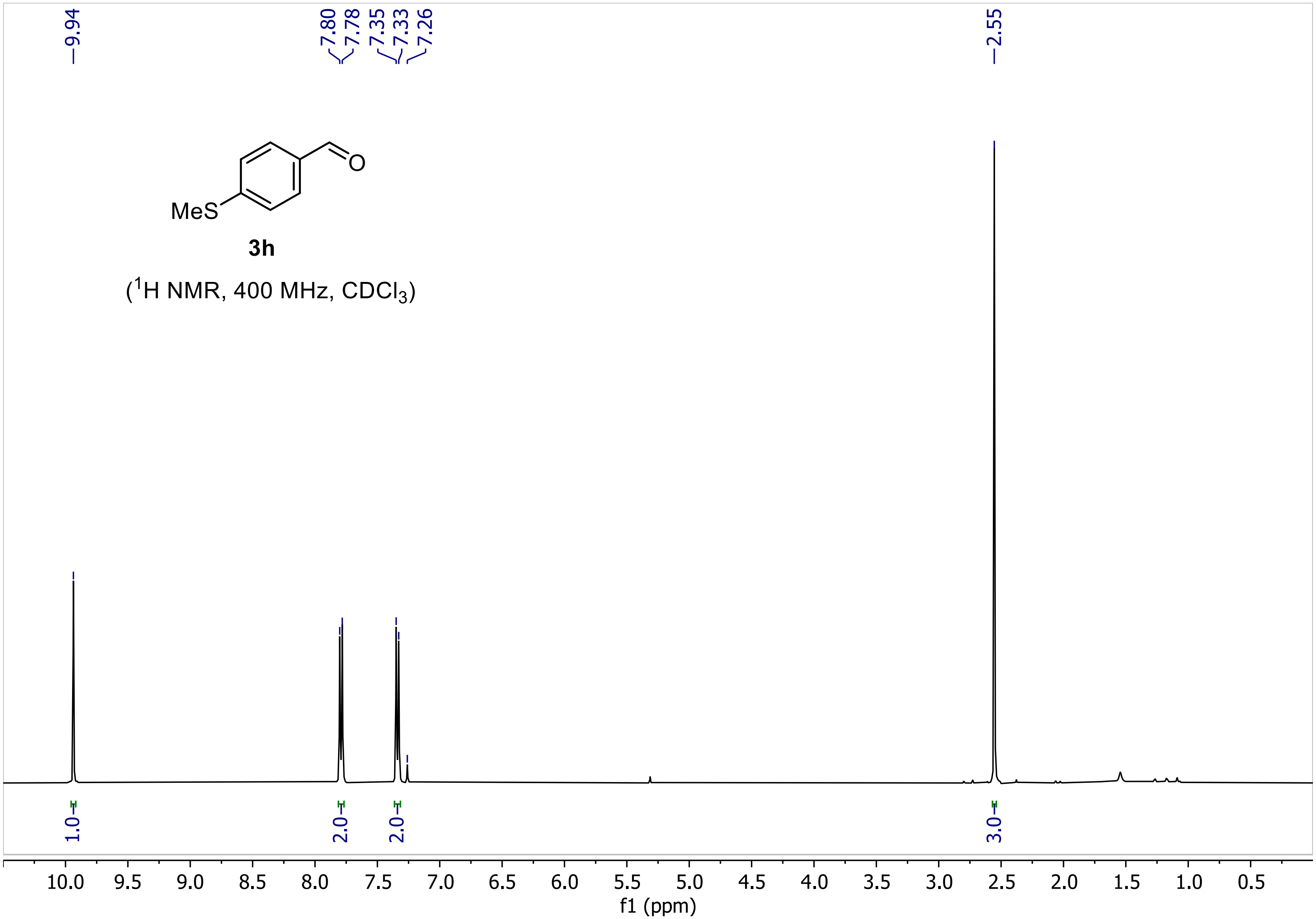

9.94
7.80
7.78
7.35
7.33
7.26
2.55
MeS
O
3h
(1H NMR, 400 MHz, CDCl3)
1.0
2.0
2.0
3.0
10.0 9.5 9.0 8.5 8.0 7.5 7.0 6.5 6.0 5.5 5.0 4.5 4.0 3.5 3.0 2.5 2.0 1.5 1.0 0.5
f1 (ppm)

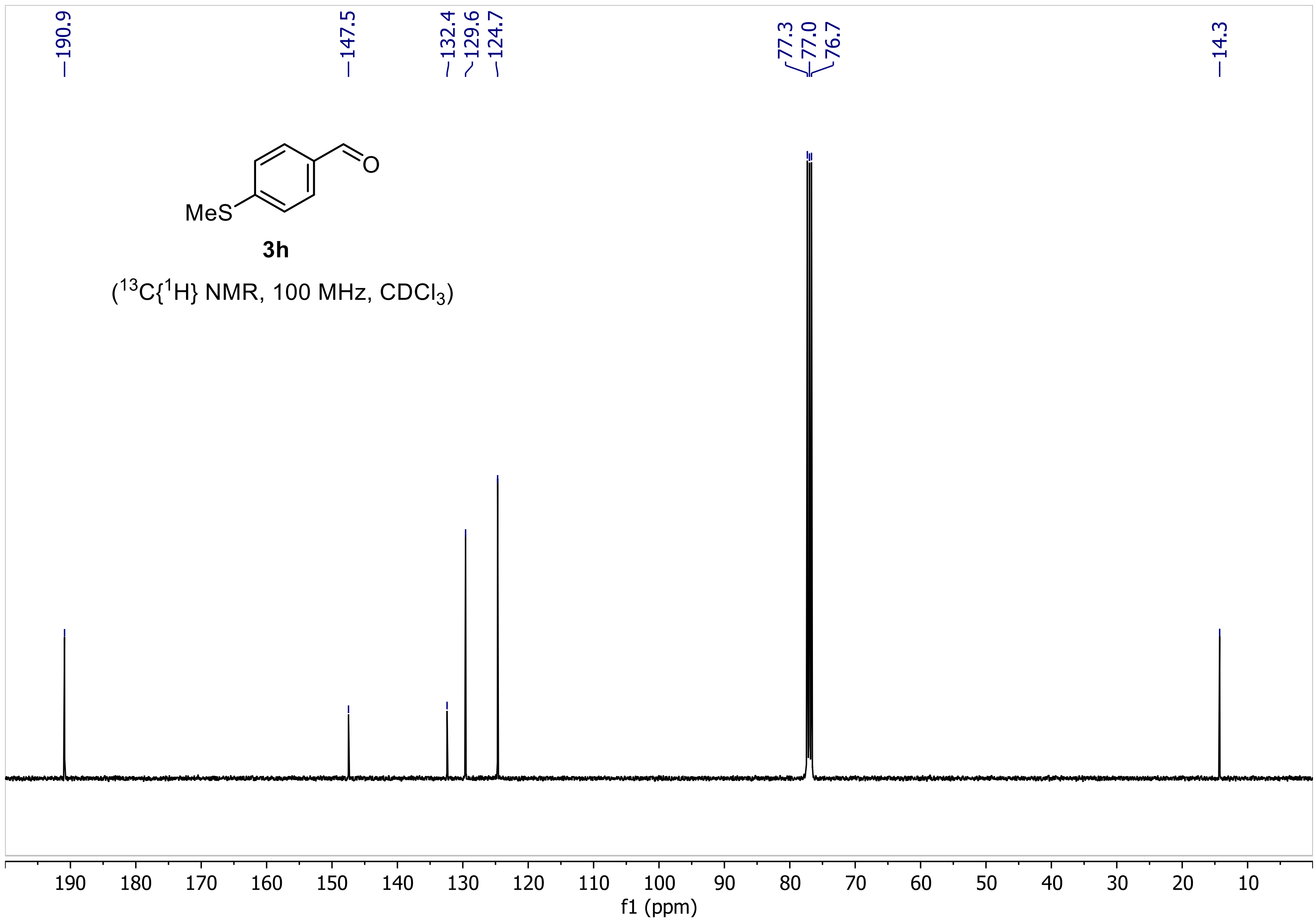

3h

($^{13}C\{^{1}H\}$ NMR, 100 MHz, $CDCl_3$)

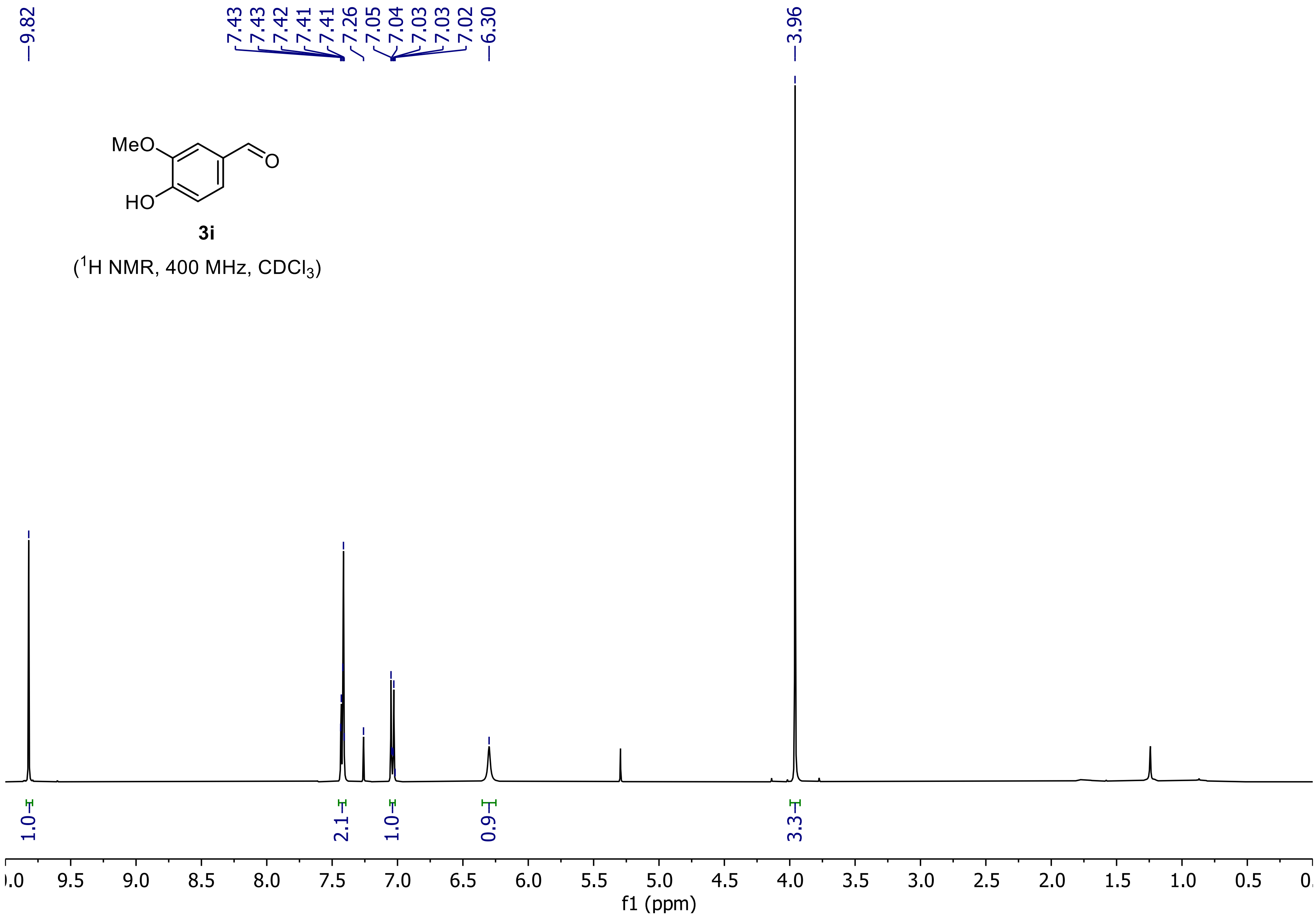

3i

(^{1}H NMR, 400 MHz, $CDCl_3$)

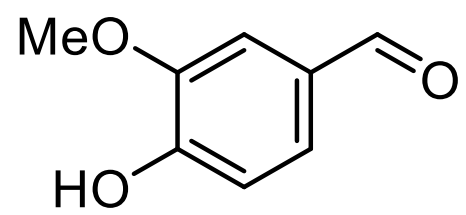

3i

($^{13}C\{^{1}H\}$ NMR, 100 MHz, $CDCl_3$)

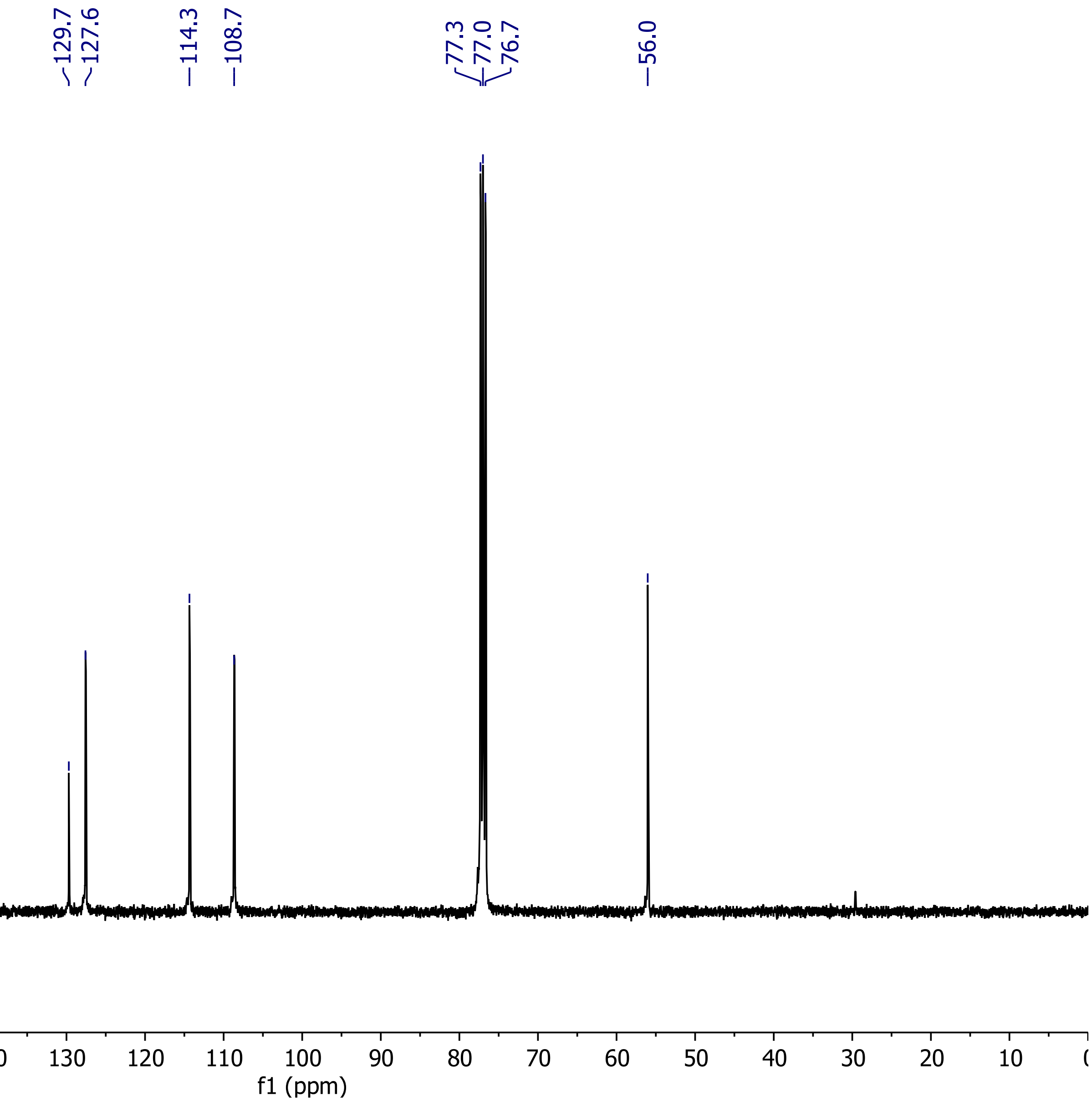

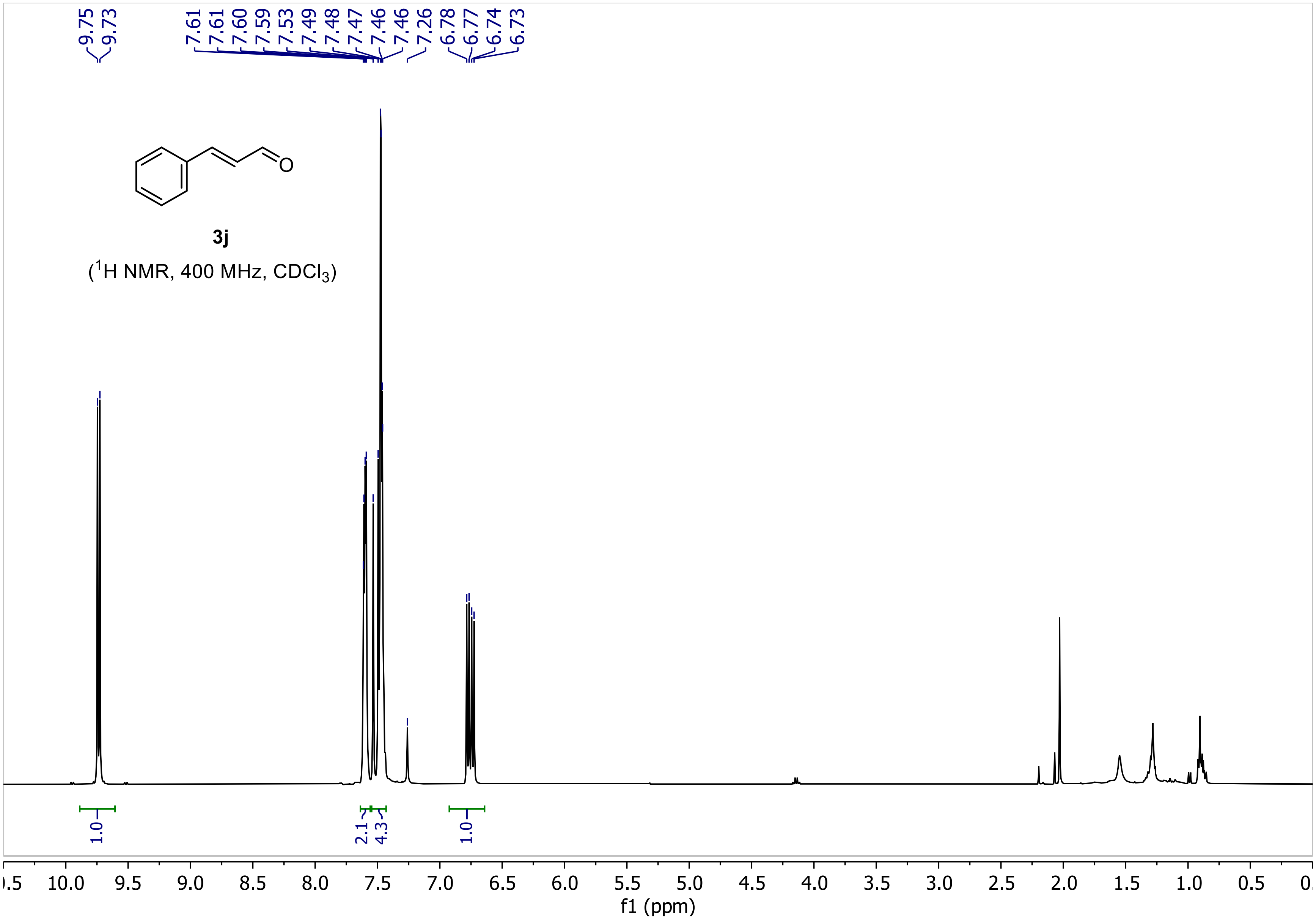

3j

(^{1}H NMR, 400 MHz, $CDCl_3$)

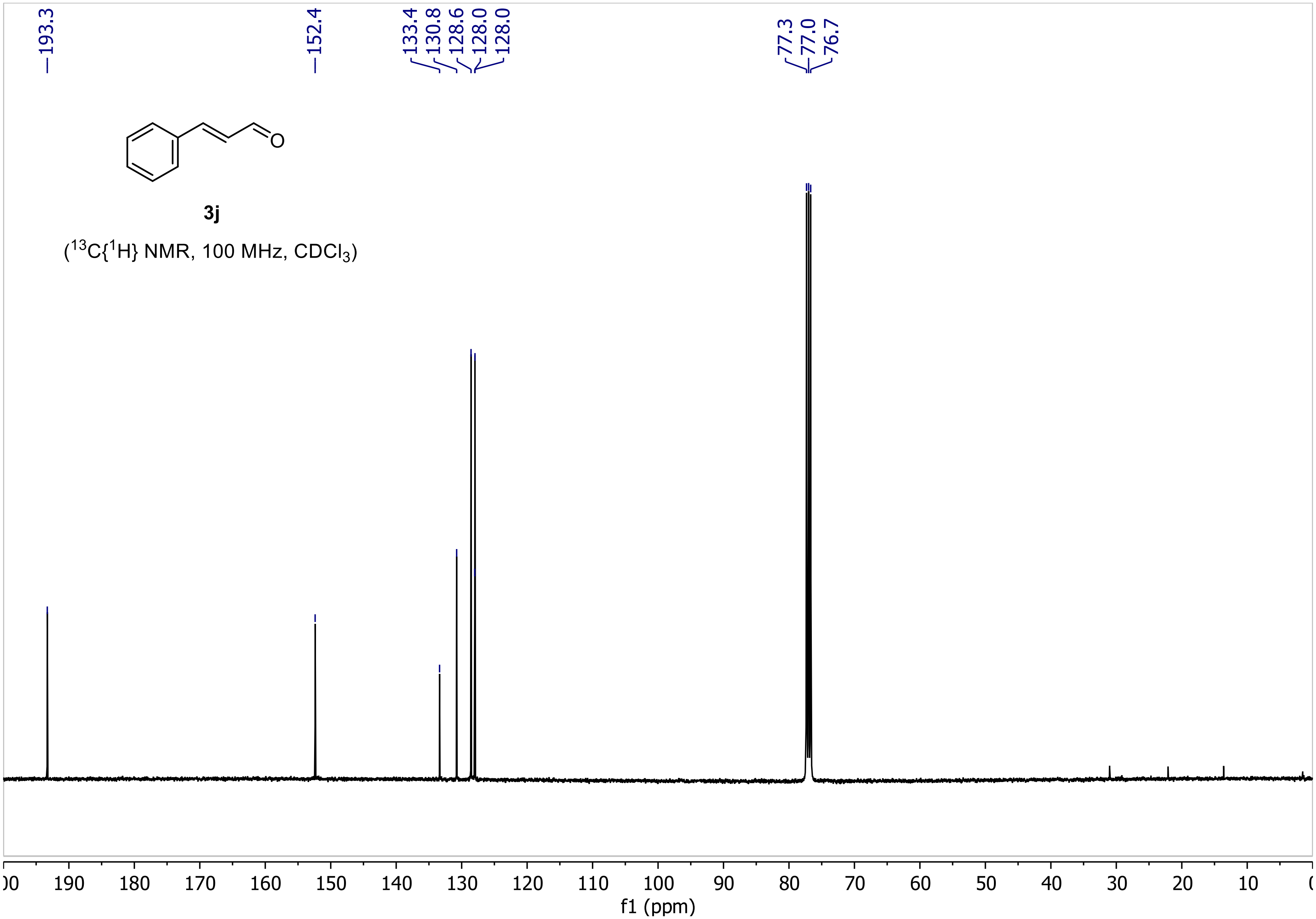

3j

($^{13}C\{^{1}H\}$ NMR, 100 MHz, $CDCl_3$)

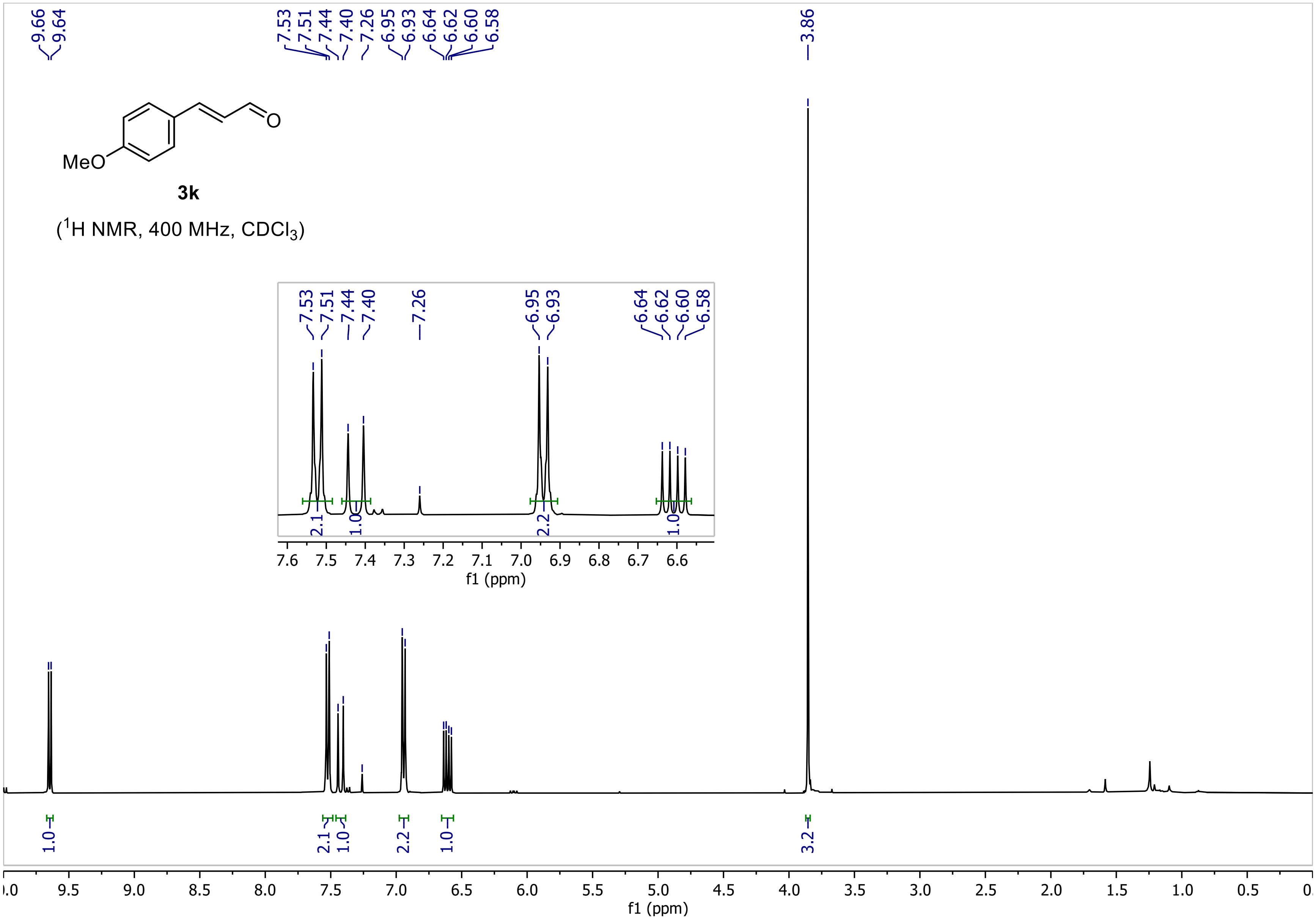
MeO
O
3k
(1H NMR, 400 MHz, CDCl3)
9.66
9.64
7.53
7.51
7.44
7.40
7.26
6.95
6.93
6.64
6.62
6.60
6.58
3.86
1.0
2.1
1.0
2.2
1.0
3.2
f1 (ppm)

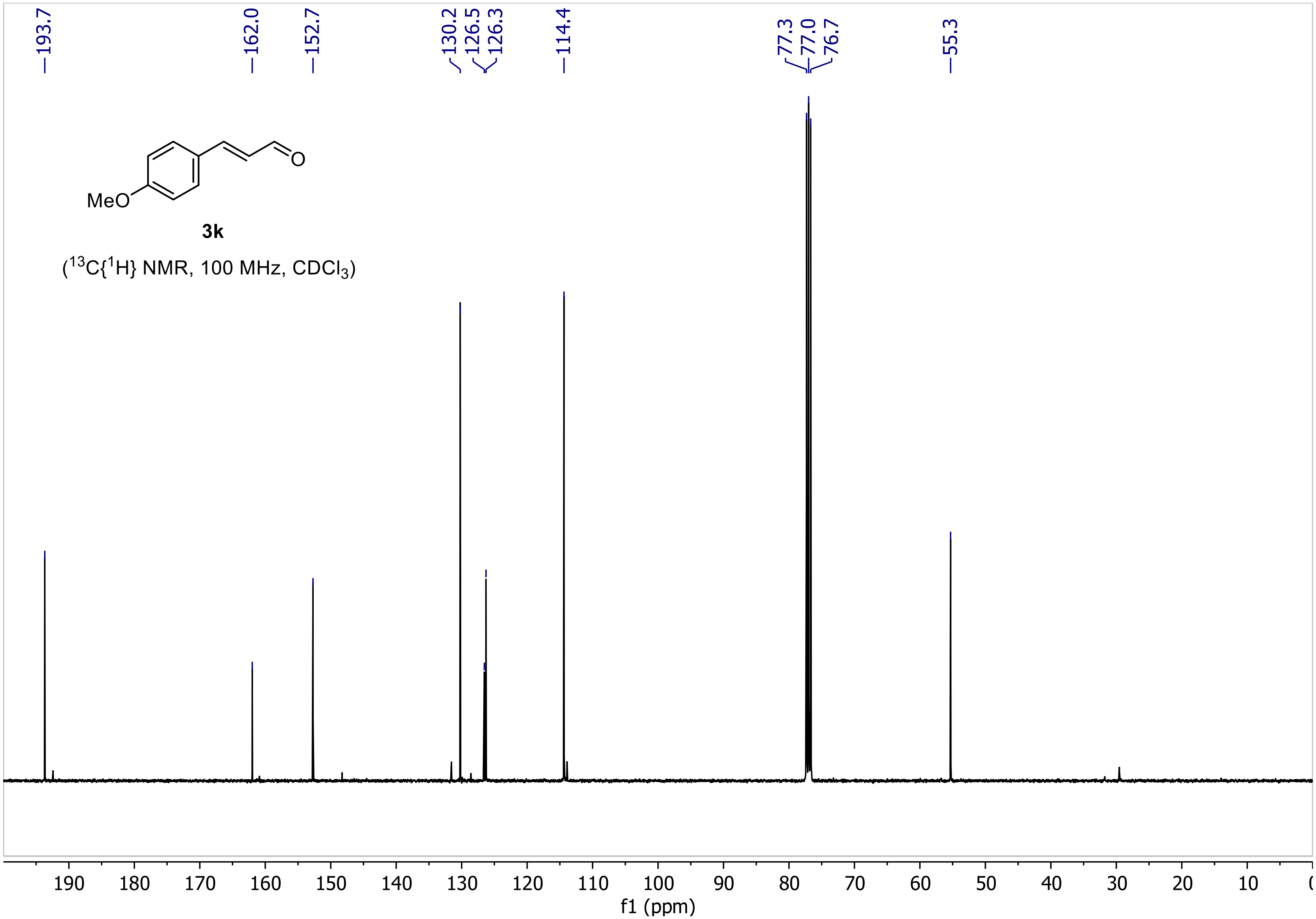

193.7
162.0
152.7
130.2
126.5
126.3
114.4
77.3
77.0
76.7
55.3
MeO
O
3k
($^{13}C\{^1H\}$ NMR, 100 MHz, $CDCl_3$)
190 180 170 160 150 140 130 120 110 100 90 80 70 60 50 40 30 20 10
f1 (ppm)

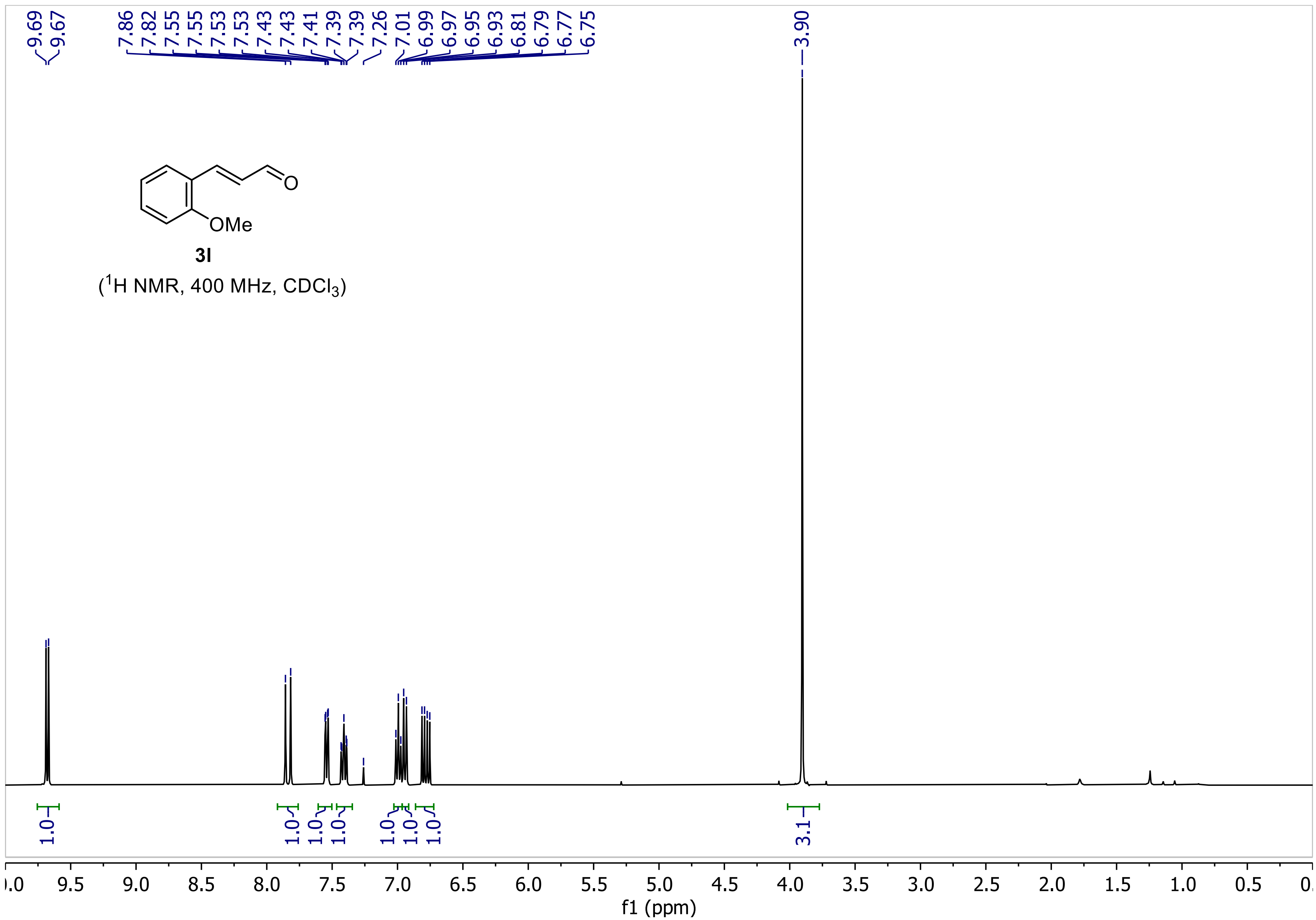

3l

(^{1}H NMR, 400 MHz, $CDCl_3$)

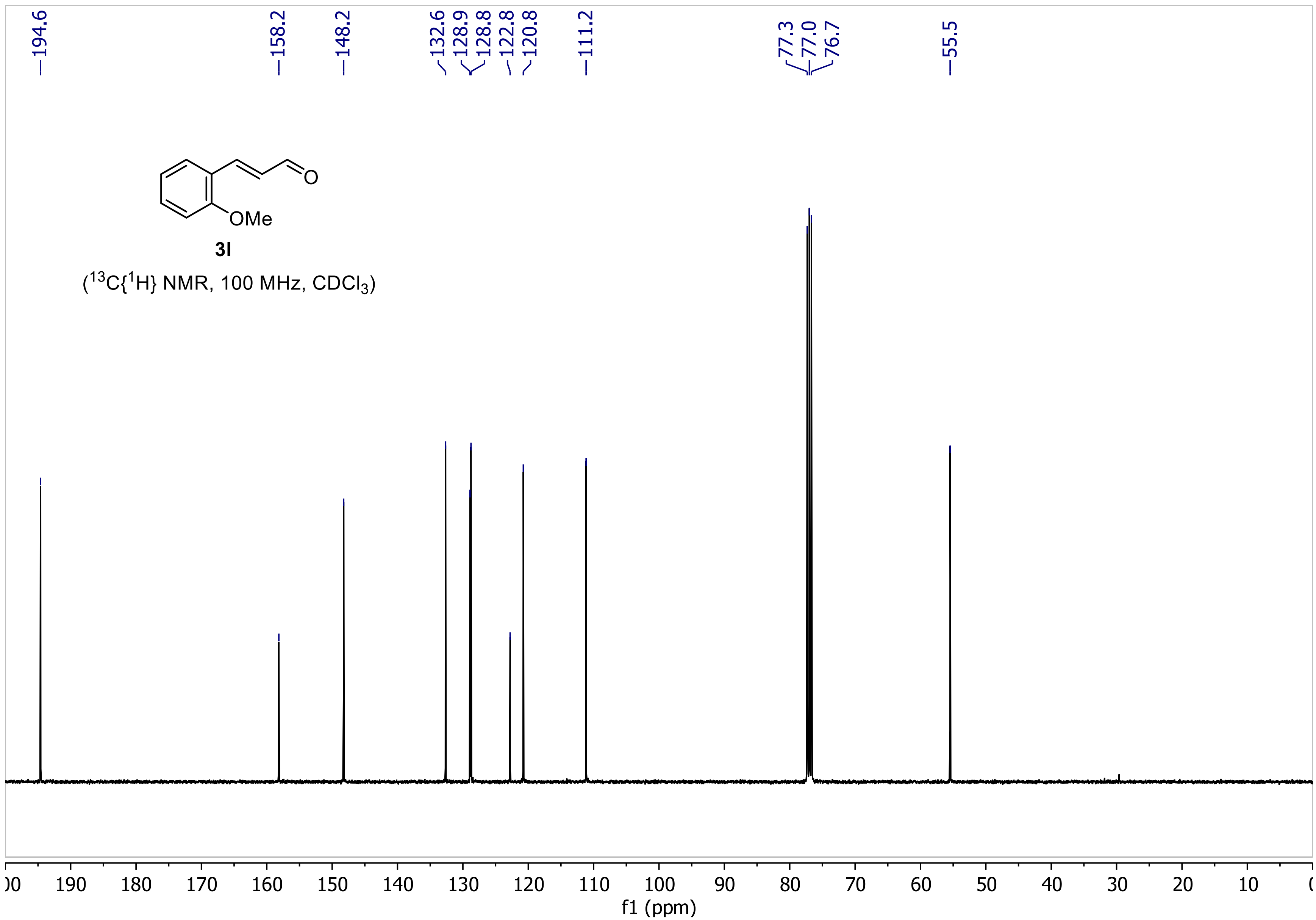
194.6
158.2
148.2
132.6
128.9
128.8
122.8
120.8
111.2
77.3
77.0
76.7
55.5
OMe
3l
(13C{1H} NMR, 100 MHz, CDCl3)
190
180
170
160
150
140
130
120
110
100
90
80
70
60
50
40
30
20
10
f1 (ppm)

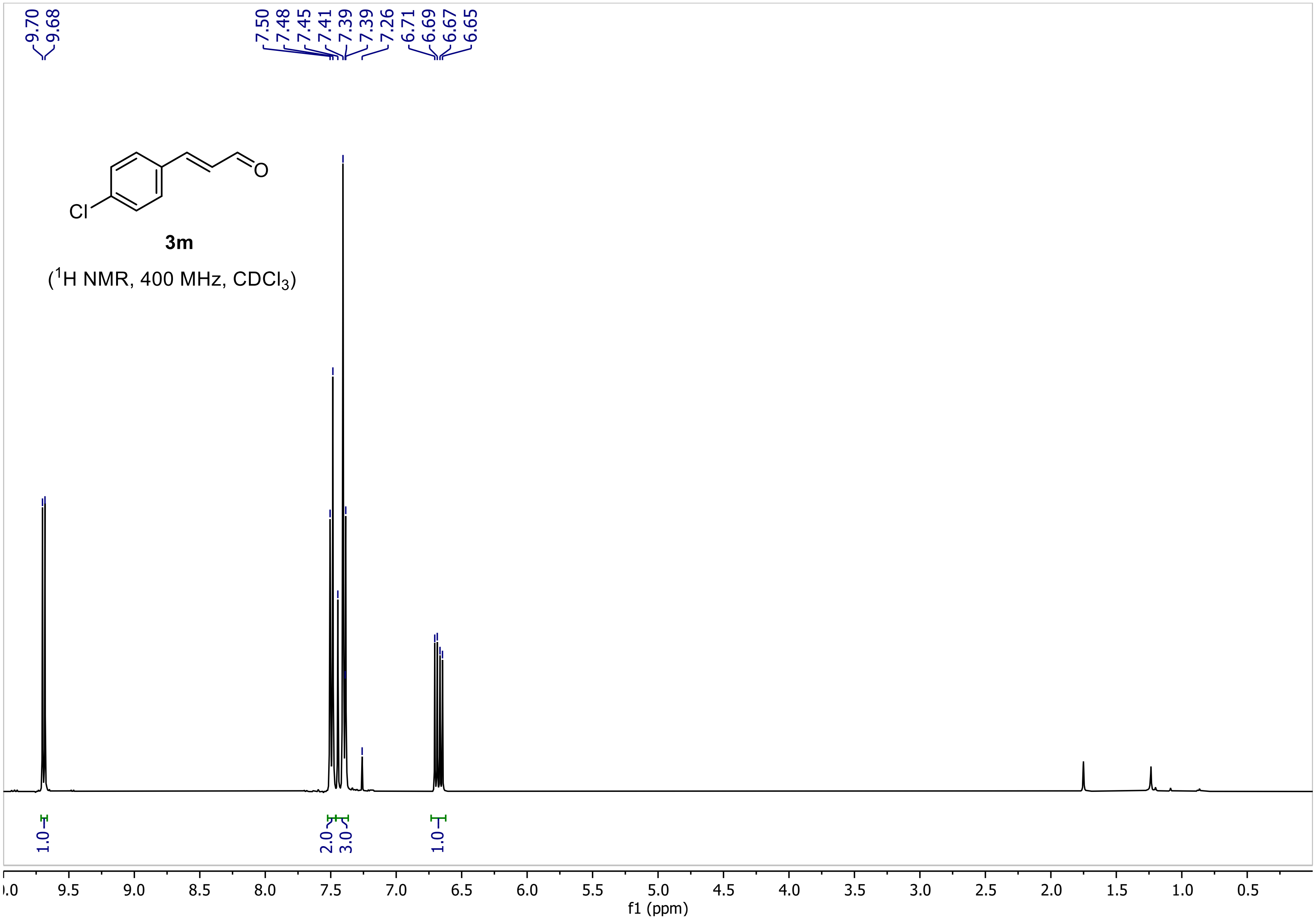
9.70
9.68
7.50
7.48
7.45
7.41
7.39
7.39
7.26
6.71
6.69
6.67
6.65
Cl
O
3m
(1H NMR, 400 MHz, CDCl3)
1.0
2.0
3.0
1.0
9.5
9.0
8.5
8.0
7.5
7.0
6.5
6.0
5.5
5.0
4.5
4.0
3.5
3.0
2.5
2.0
1.5
1.0
0.5
f1 (ppm)

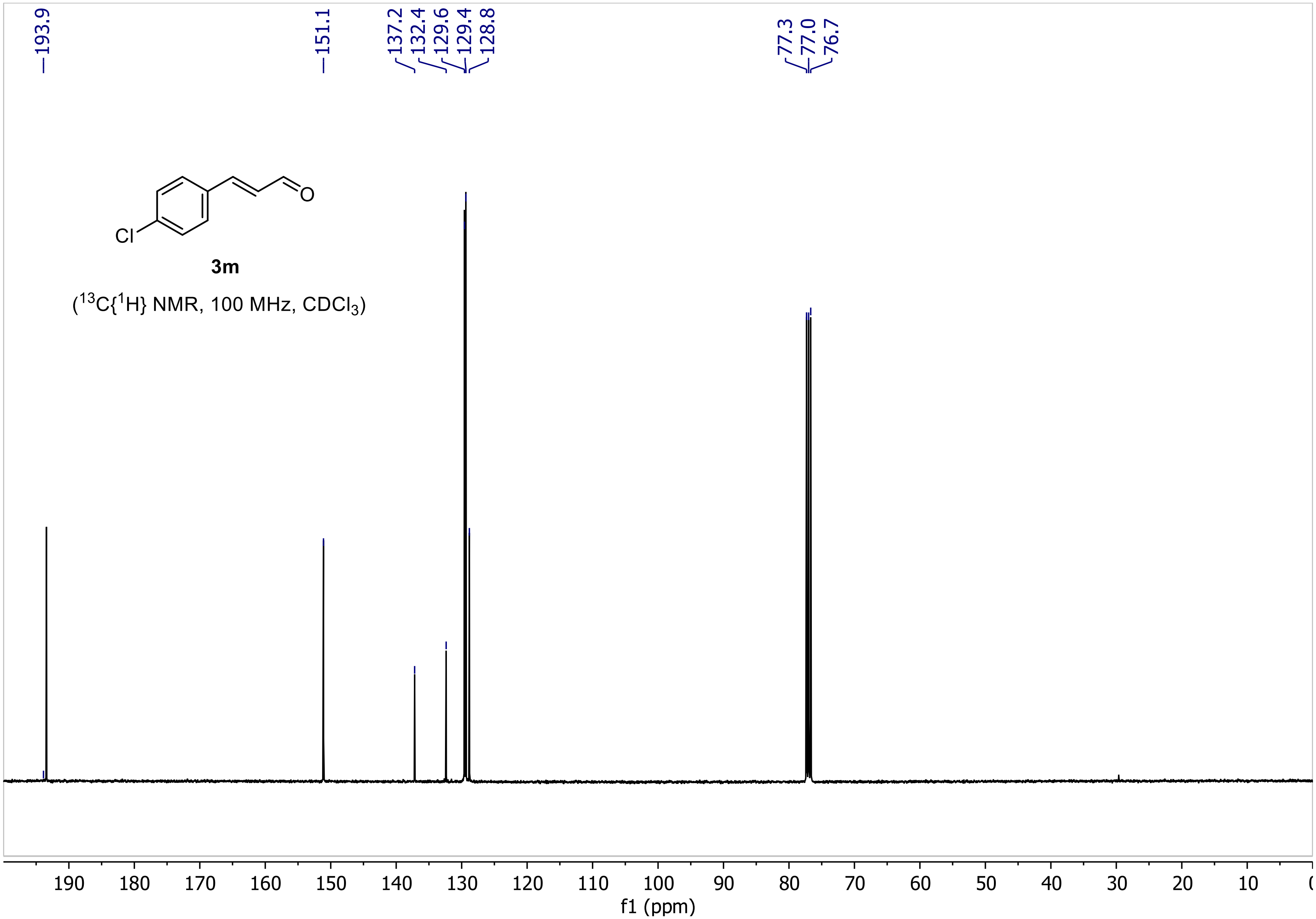
193.9
151.1
137.2
132.4
129.6
129.4
128.8
77.3
77.0
76.7
Cl
O
3m
(13C{1H} NMR, 100 MHz, CDCl3)
190 180 170 160 150 140 130 120 110 100 90 80 70 60 50 40 30 20 10
f1 (ppm)

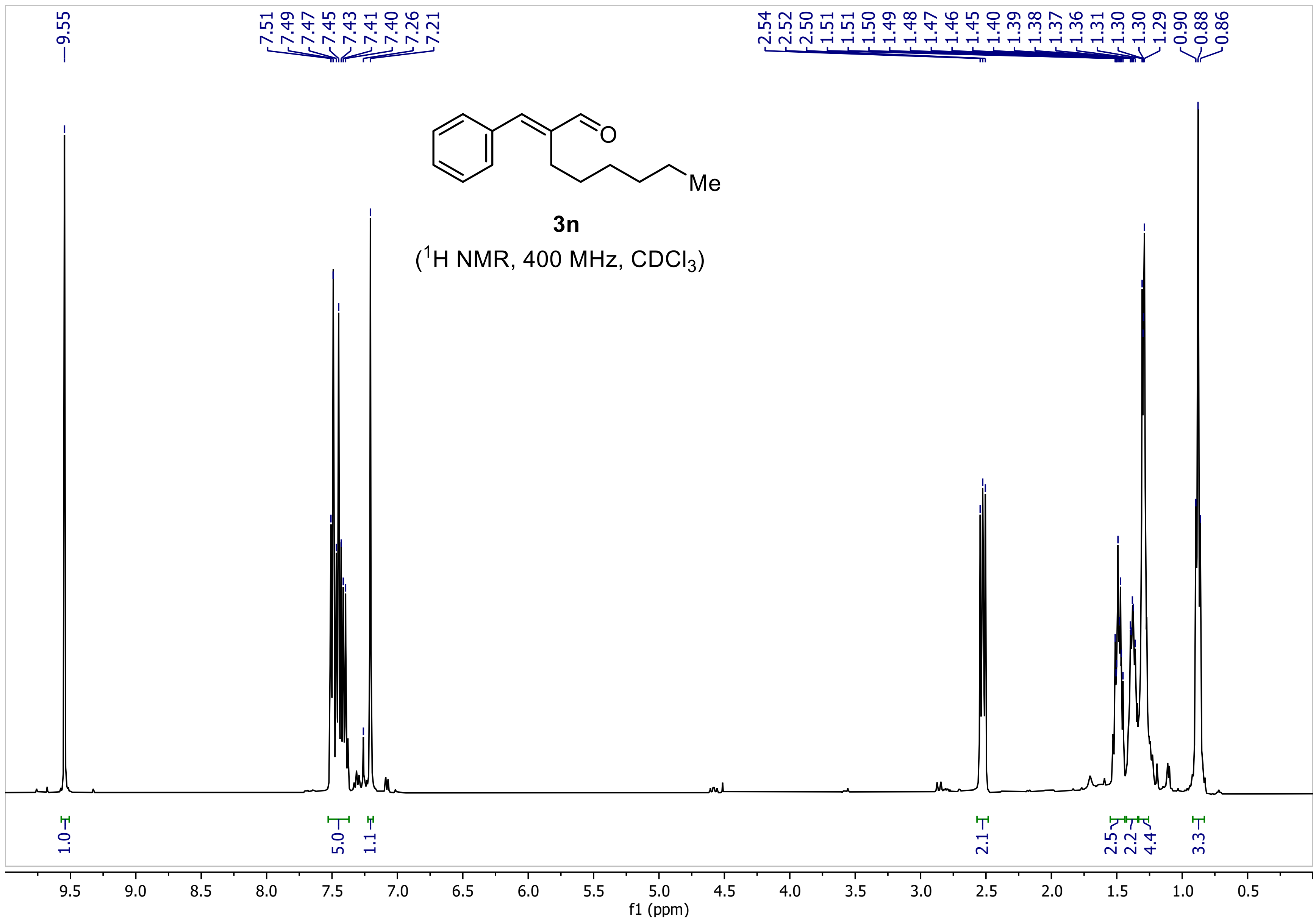
9.55
7.51
7.49
7.47
7.45
7.43
7.41
7.40
7.26
7.21
2.54
2.52
2.50
1.51
1.51
1.50
1.49
1.48
1.47
1.46
1.45
1.40
1.39
1.38
1.37
1.36
1.31
1.30
1.30
1.29
0.90
0.88
0.86
O
Me
3n
(^{1}H NMR, 400 MHz, $CDCl_3$)
1.0
5.0
1.1
2.1
2.5
2.2
4.4
3.3
9.5
9.0
8.5
8.0
7.5
7.0
6.5
6.0
5.5
5.0
4.5
4.0
3.5
3.0
2.5
2.0
1.5
1.0
0.5
f1 (ppm)

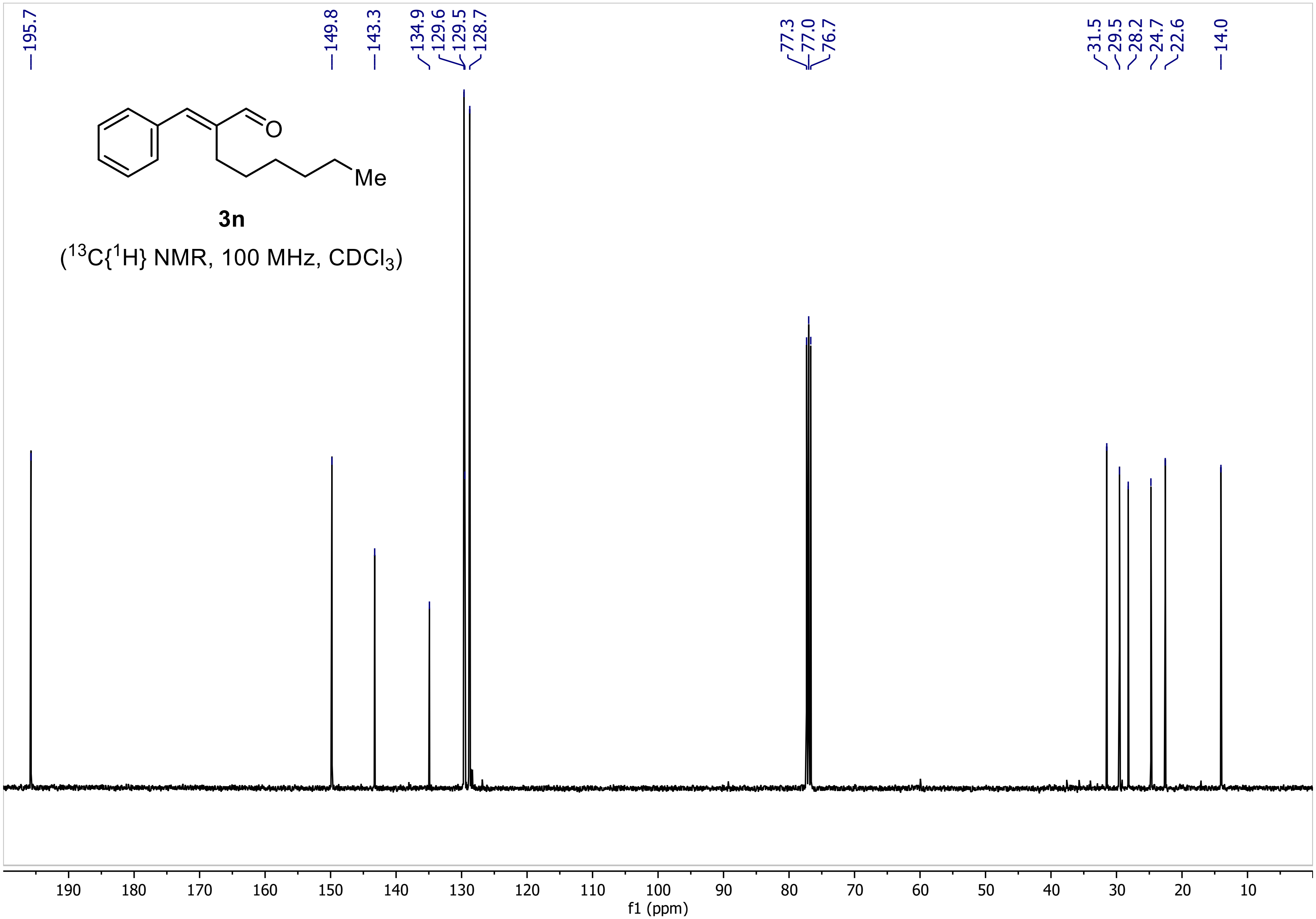
195.7
149.8
143.3
134.9
129.6
129.5
128.7
77.3
77.0
76.7
31.5
29.5
28.2
24.7
22.6
14.0
Me
O
3n
($^{13}C\{^{1}H\}$ NMR, 100 MHz, $CDCl_3$)
190 180 170 160 150 140 130 120 110 100 90 80 70 60 50 40 30 20 10
f1 (ppm)

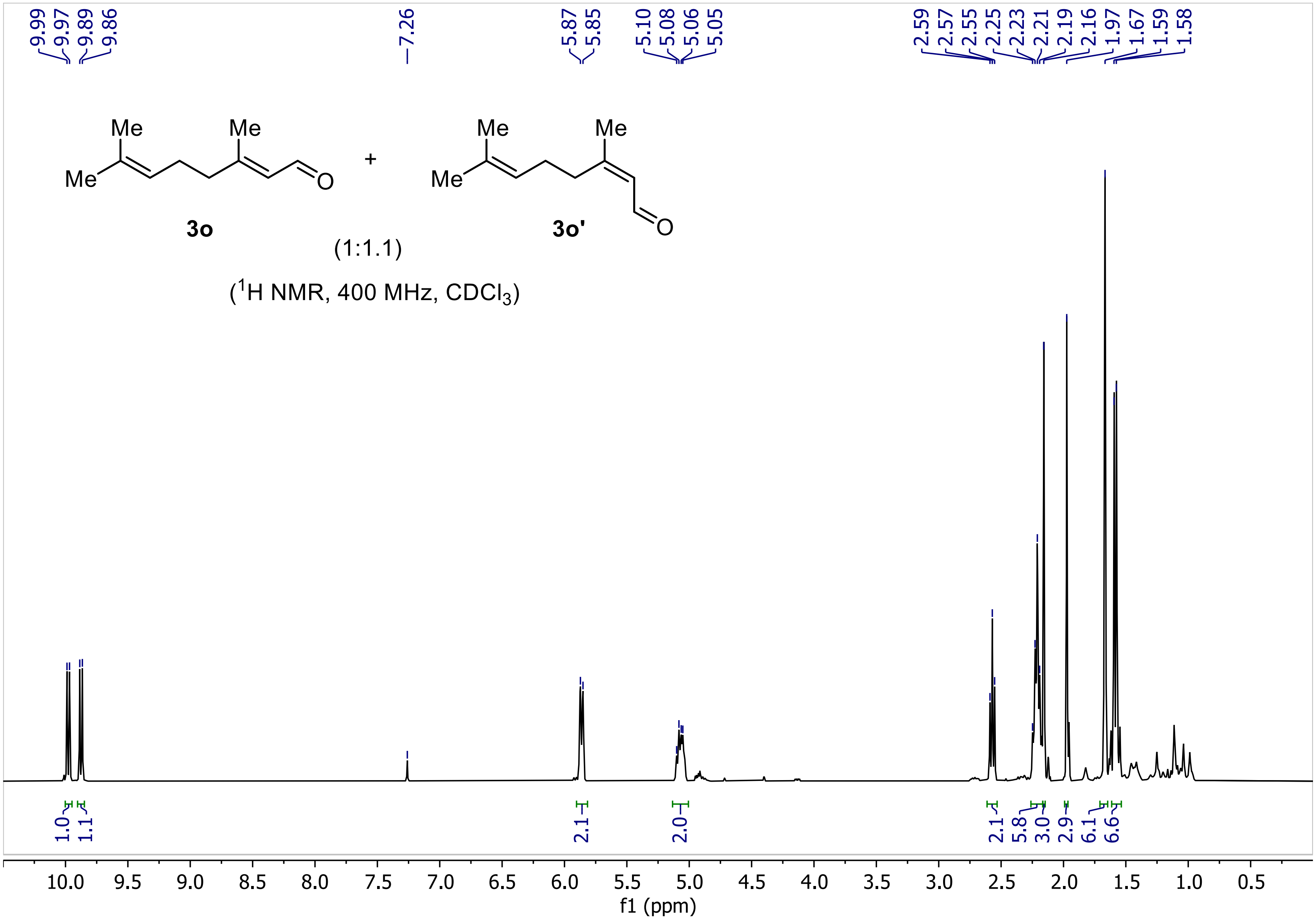
9.99 9.97 9.89 9.86
7.26
5.87 5.85
5.10 5.08 5.06 5.05
2.59 2.57 2.55 2.25 2.23 2.21 2.19 2.16 1.97 1.67 1.59 1.58
Me
Me
Me
O
3o
+
Me
Me
Me
O
3o'
(1:1.1)
(^{1}H NMR, 400 MHz, $CDCl_3$)
1.0 1.1 2.1 2.0 2.1 5.8 3.0 2.9 6.1 6.6
10.0 9.5 9.0 8.5 8.0 7.5 7.0 6.5 6.0 5.5 5.0 4.5 4.0 3.5 3.0 2.5 2.0 1.5 1.0 0.5
f1 (ppm)

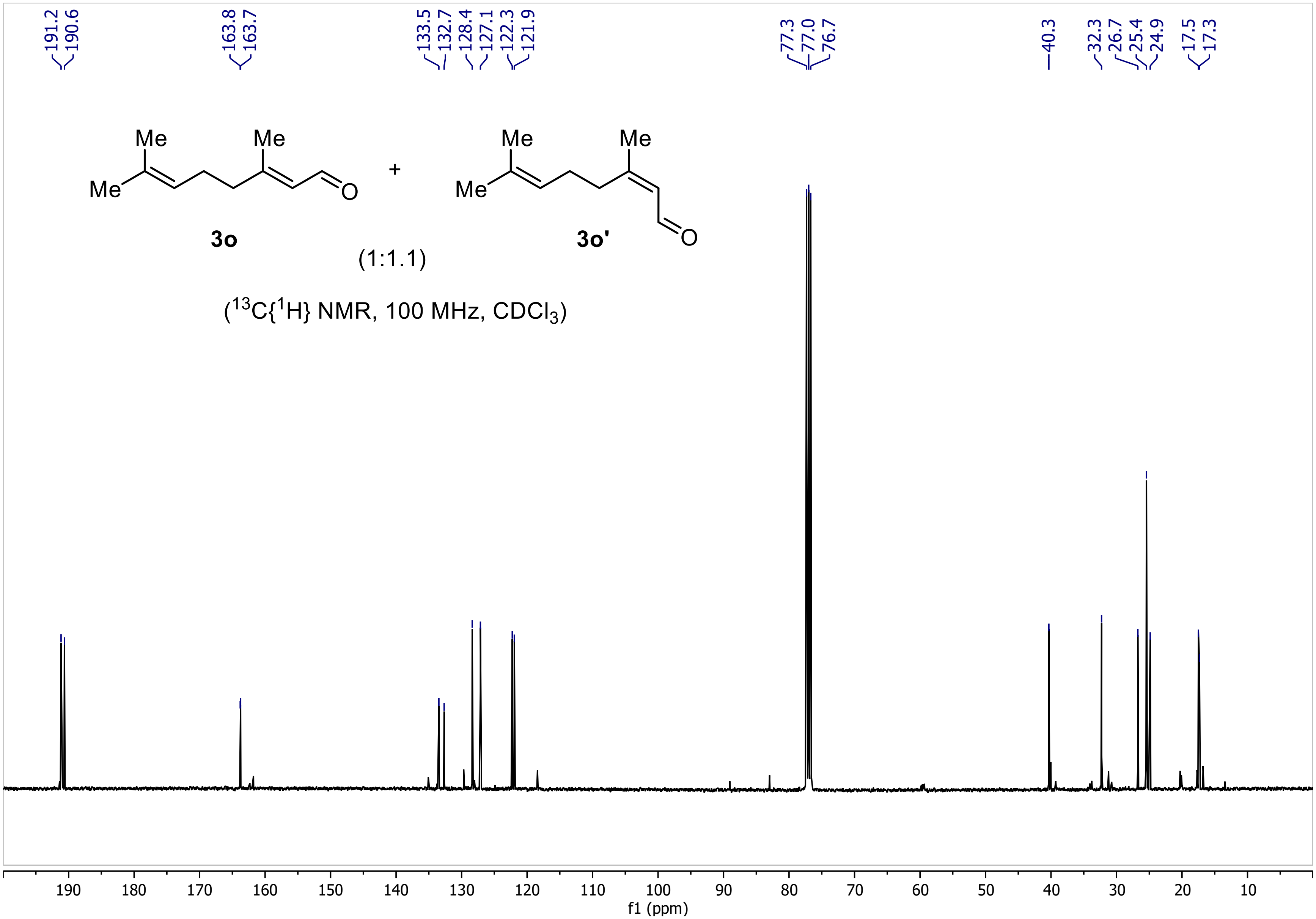
191.2
190.6
163.8
163.7
133.5
132.7
128.4
127.1
122.3
121.9
77.3
77.0
76.7
40.3
32.3
26.7
25.4
24.9
17.5
17.3
Me
Me
Me
O
3o
+
Me
Me
Me
O
3o'
(1:1.1)
($^{13}C\{^{1}H\}$ NMR, 100 MHz, $CDCl_3$)
190
180
170
160
150
140
130
120
110
100
90
80
70
60
50
40
30
20
10
f1 (ppm)

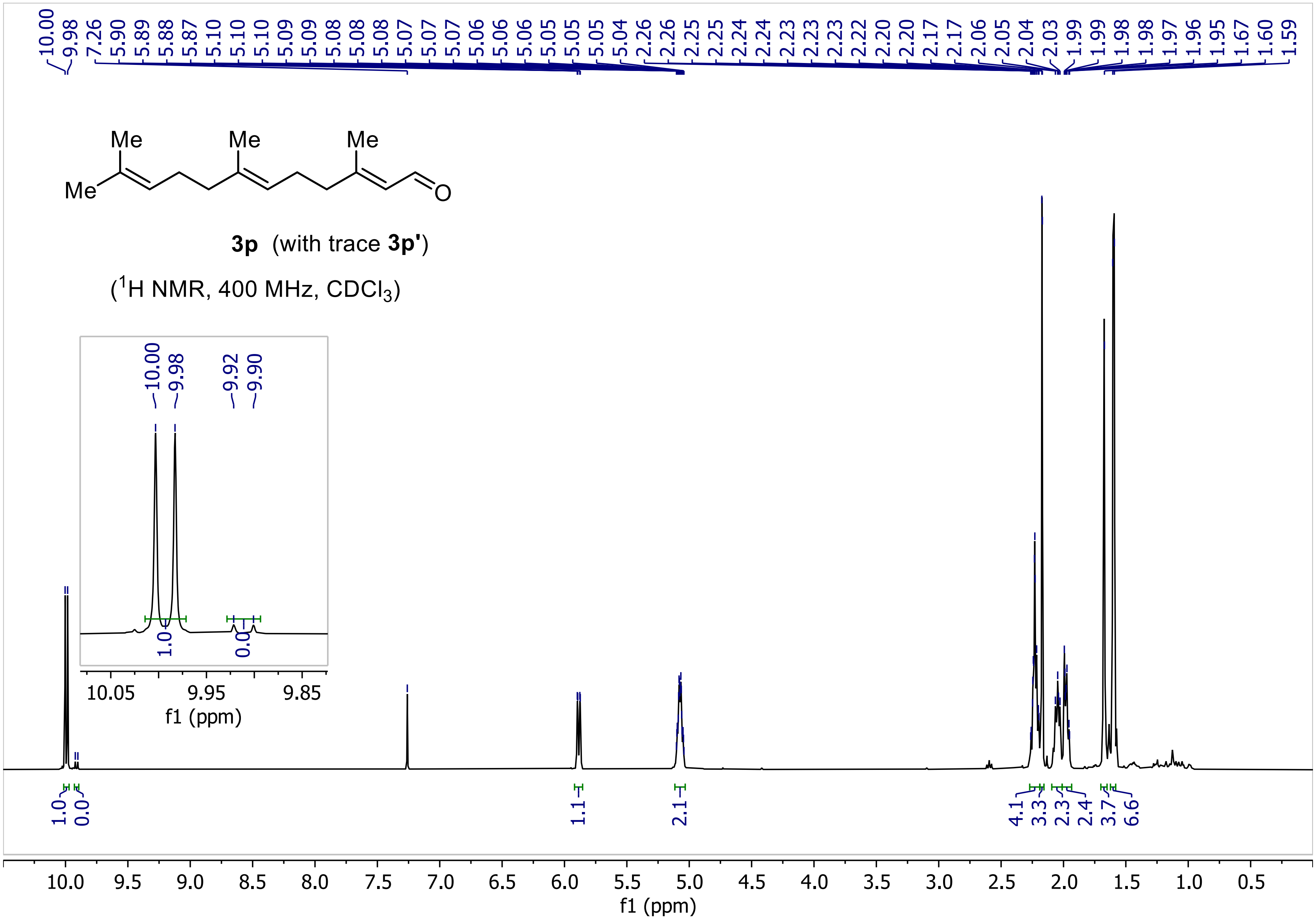

Me
Me
Me
Me
O
3p (with trace 3p')
(¹H NMR, 400 MHz, CDCl₃)
f1 (ppm)

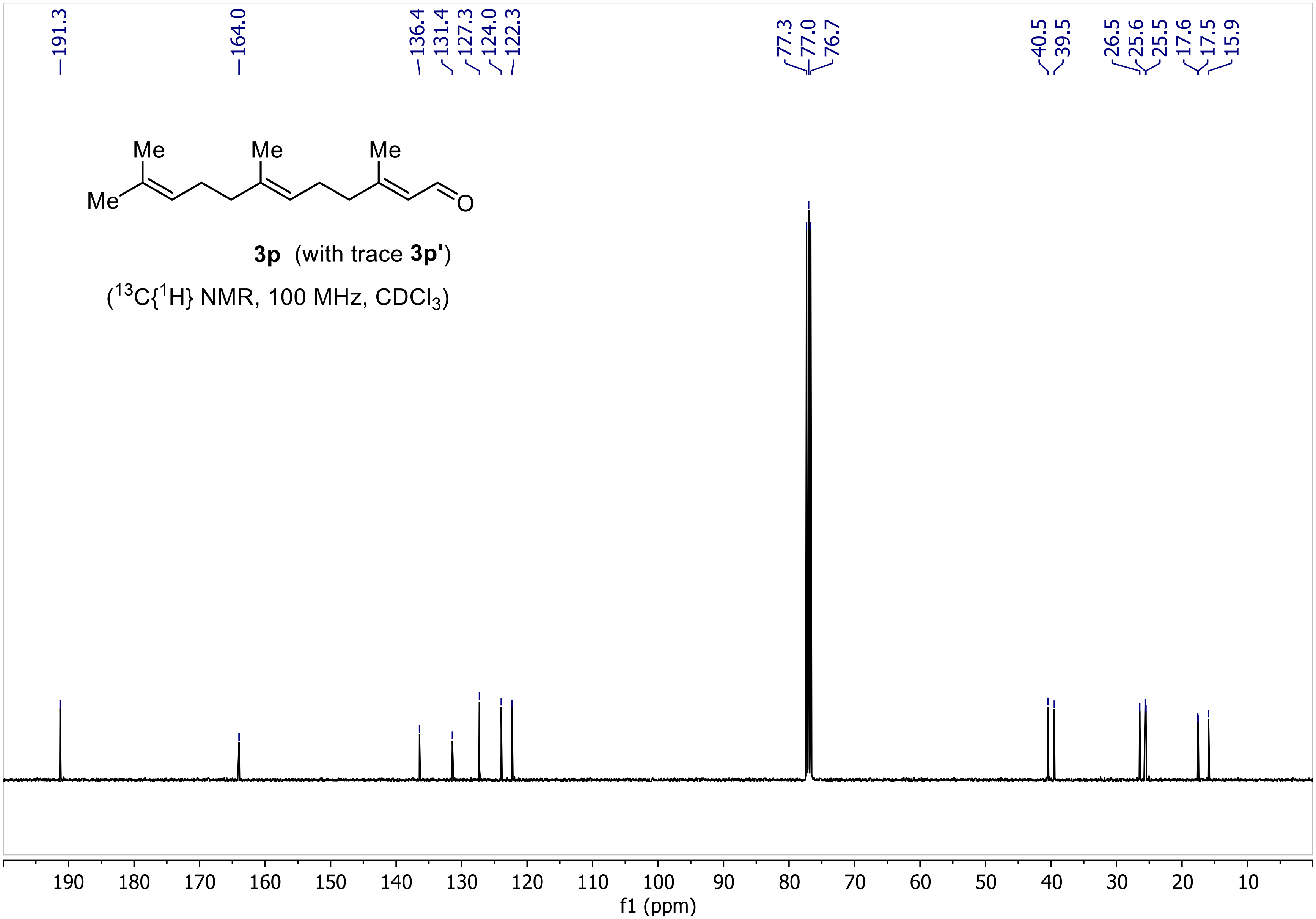
191.3
164.0
136.4
131.4
127.3
124.0
122.3
77.3
77.0
76.7
40.5
39.5
26.5
25.6
25.5
17.6
17.5
15.9
Me
Me
Me
Me
O
3p (with trace **3p'**)
($^{13}C\{^{1}H\}$ NMR, 100 MHz, $CDCl_3$)
190
180
170
160
150
140
130
120
110
100
90
80
70
60
50
40
30
20
10
f1 (ppm)

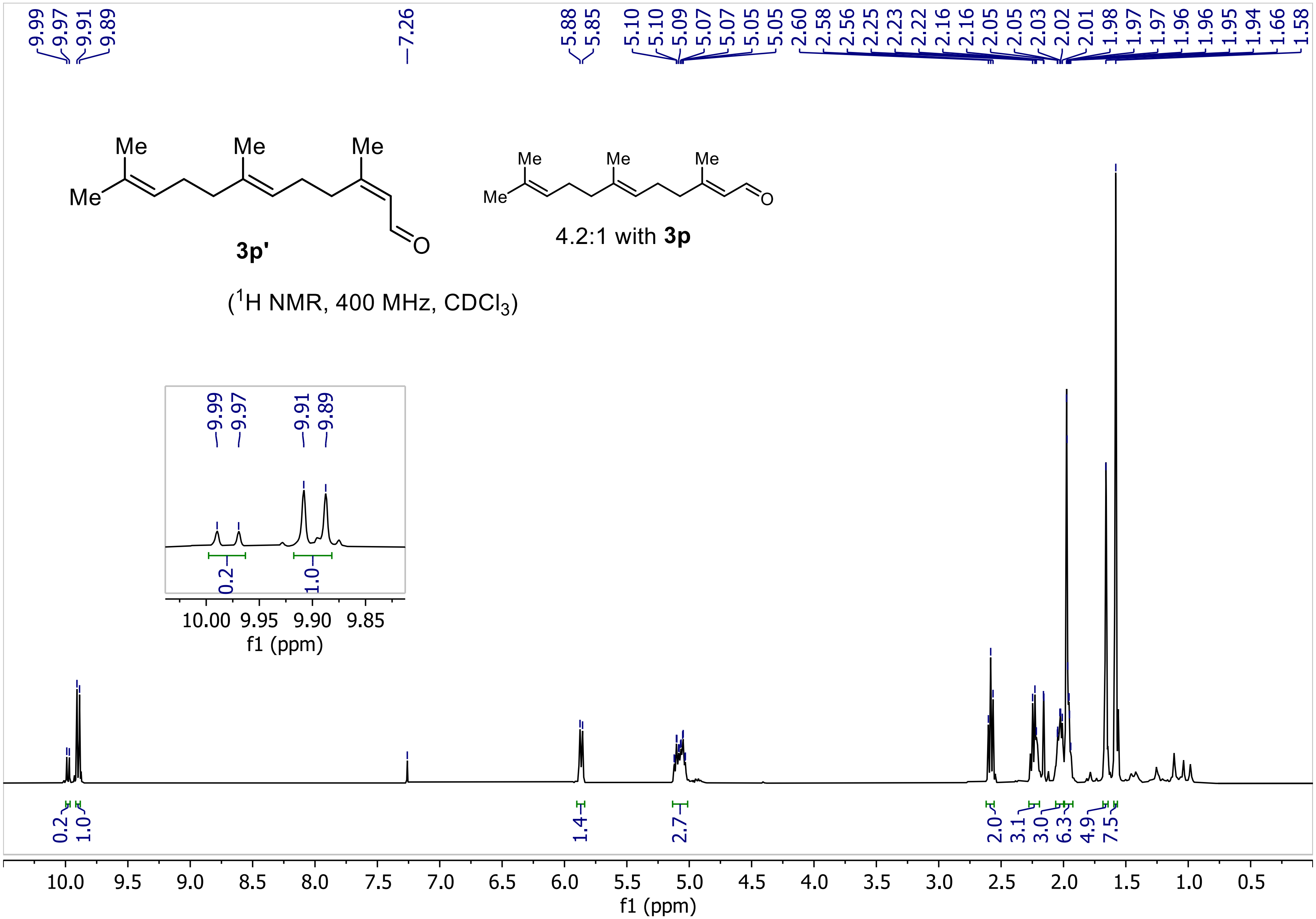
3p'
4.2:1 with 3p
(1H NMR, 400 MHz, CDCl3)
f1 (ppm)

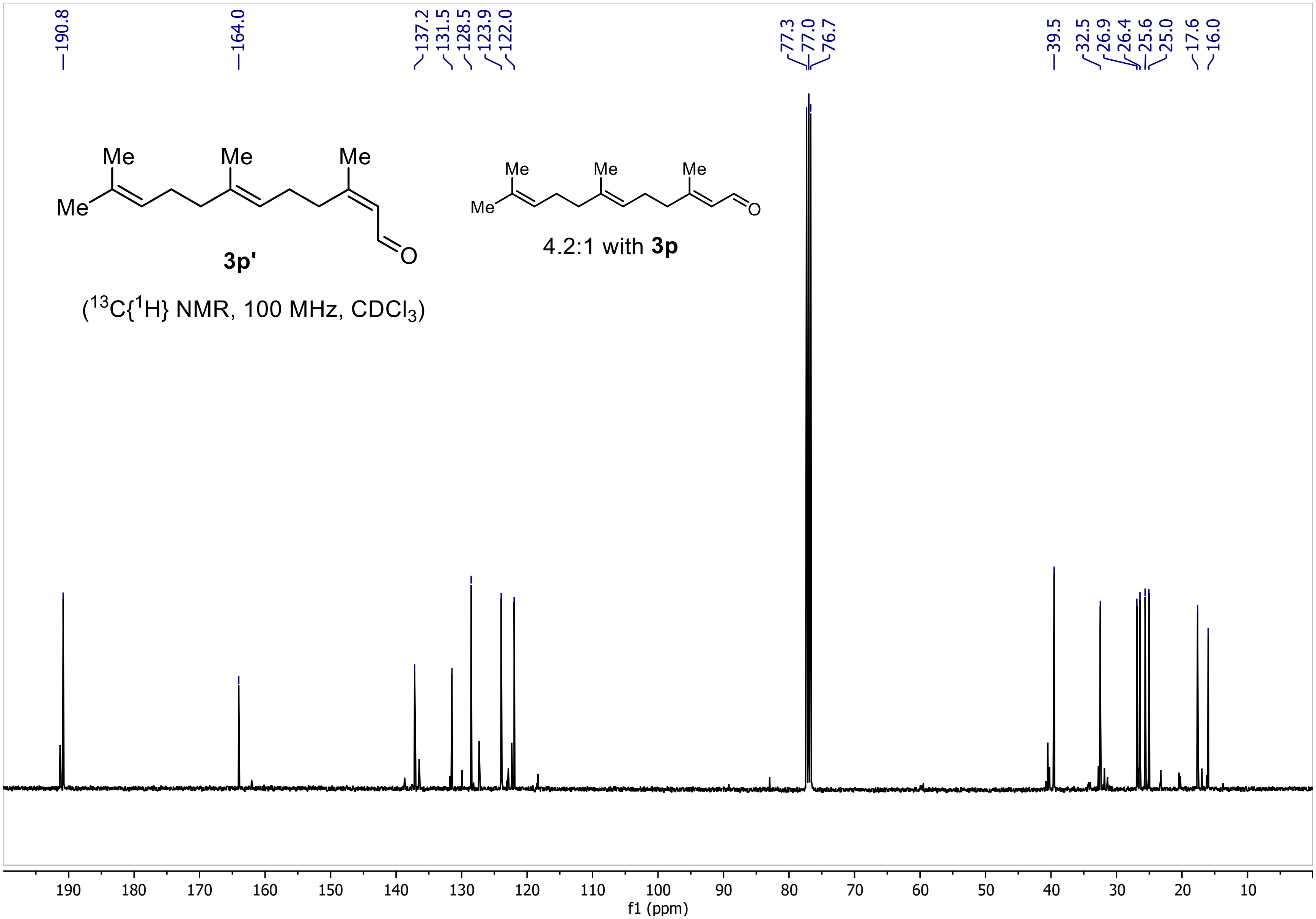

Me
Me
Me
Me
3p'
(13C{1H} NMR, 100 MHz, CDCl3)
Me
Me
Me
Me
O
4.2:1 with 3p
190.8
164.0
137.2
131.5
128.5
123.9
122.0
77.3
77.0
76.7
39.5
32.5
26.9
26.4
25.6
25.0
17.6
16.0
190
180
170
160
150
140
130
120
110
100
90
80
70
60
50
40
30
20
10
f1 (ppm)

3q

(^{1}H NMR, 400 MHz, $CDCl_3$)

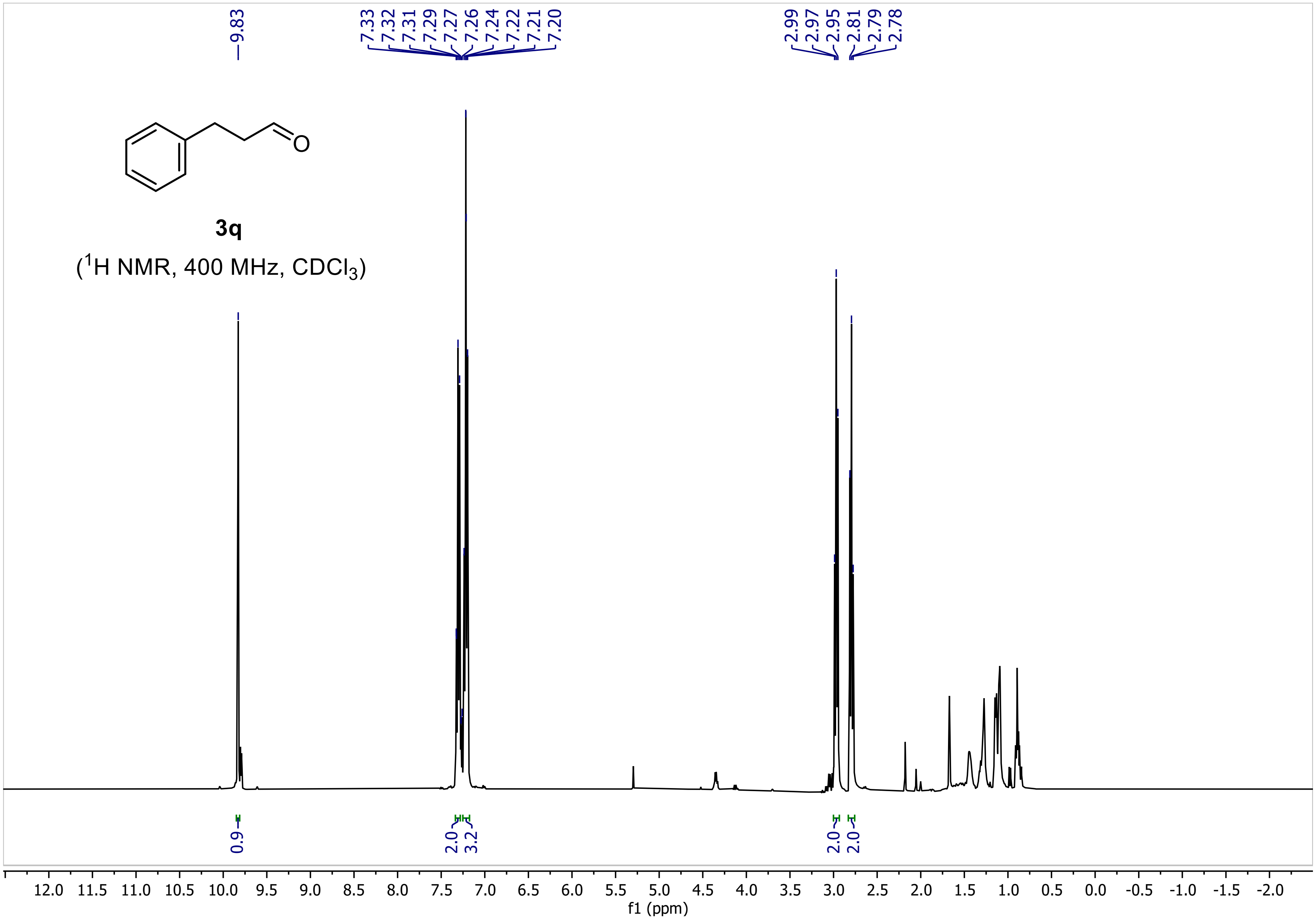

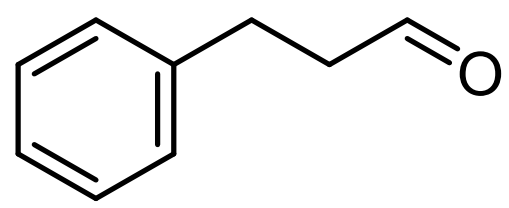

3q

($^{13}C\{^{1}H\}$ NMR, 100 MHz, $CDCl_3$)

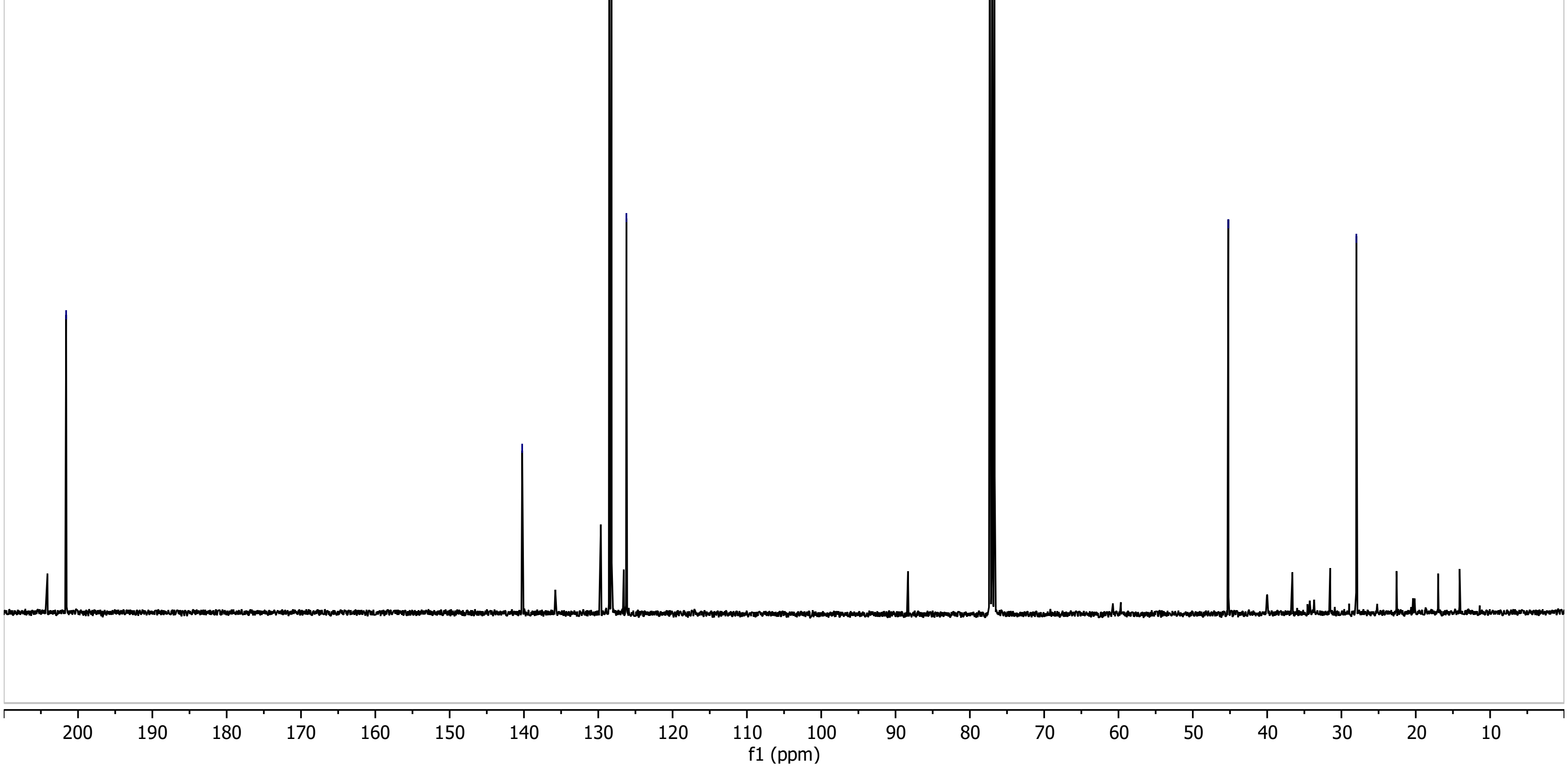

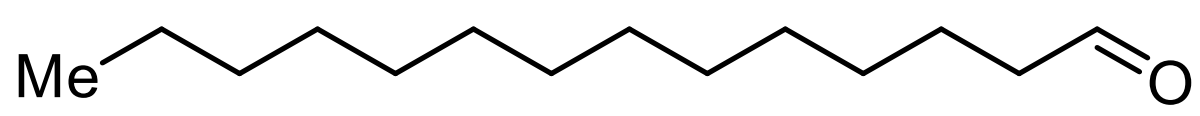

3r
(^{1}H NMR, 400 MHz, $CDCl_3$)

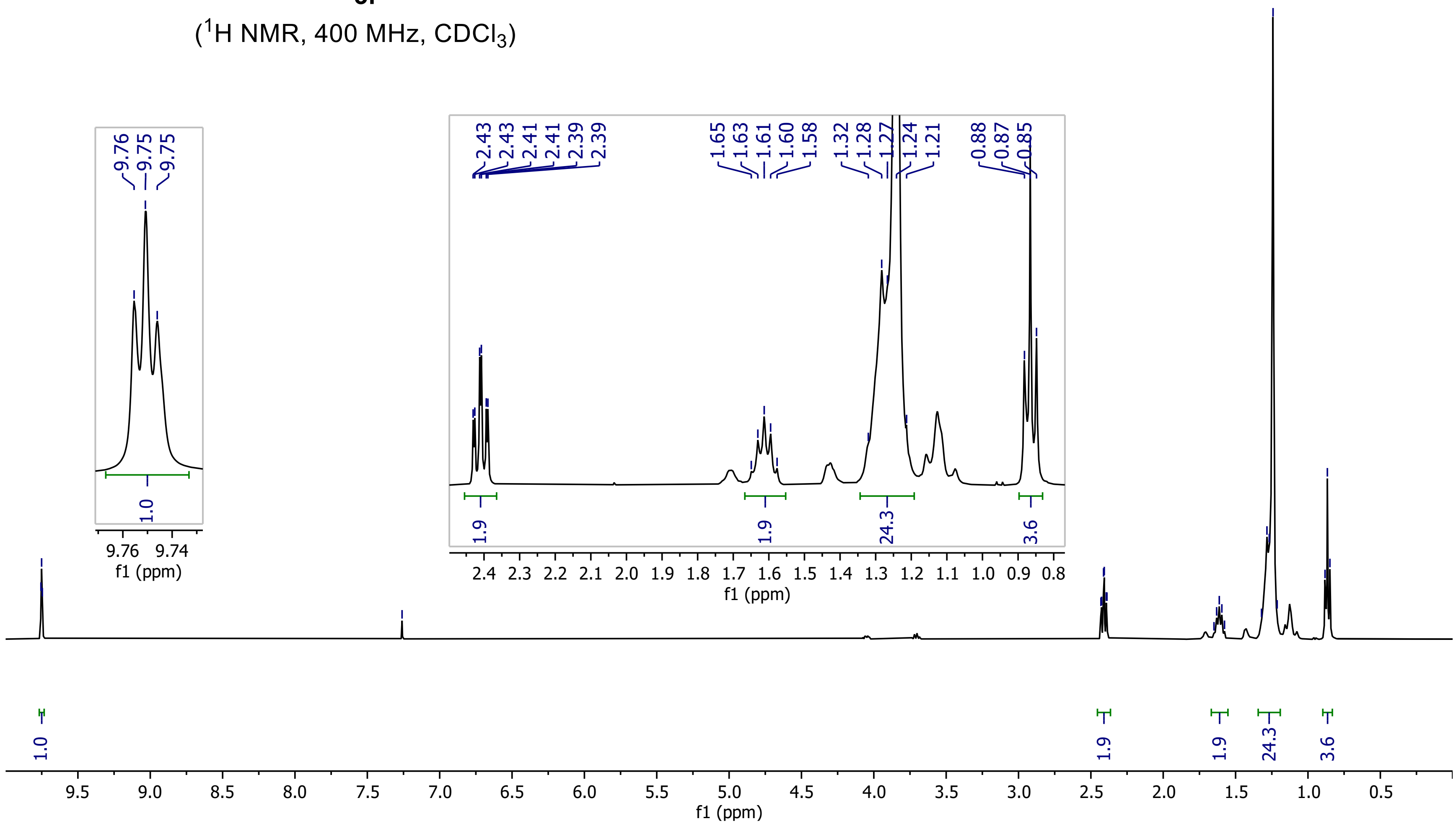

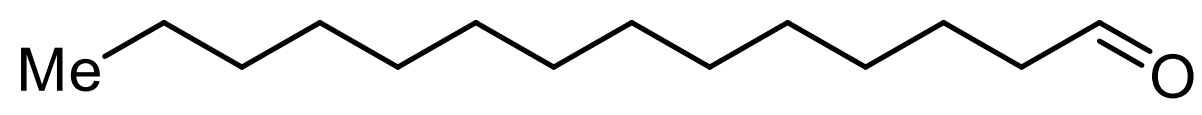

3r

($^{13}C\{^{1}H\}$ NMR, 100 MHz, $CDCl_3$)

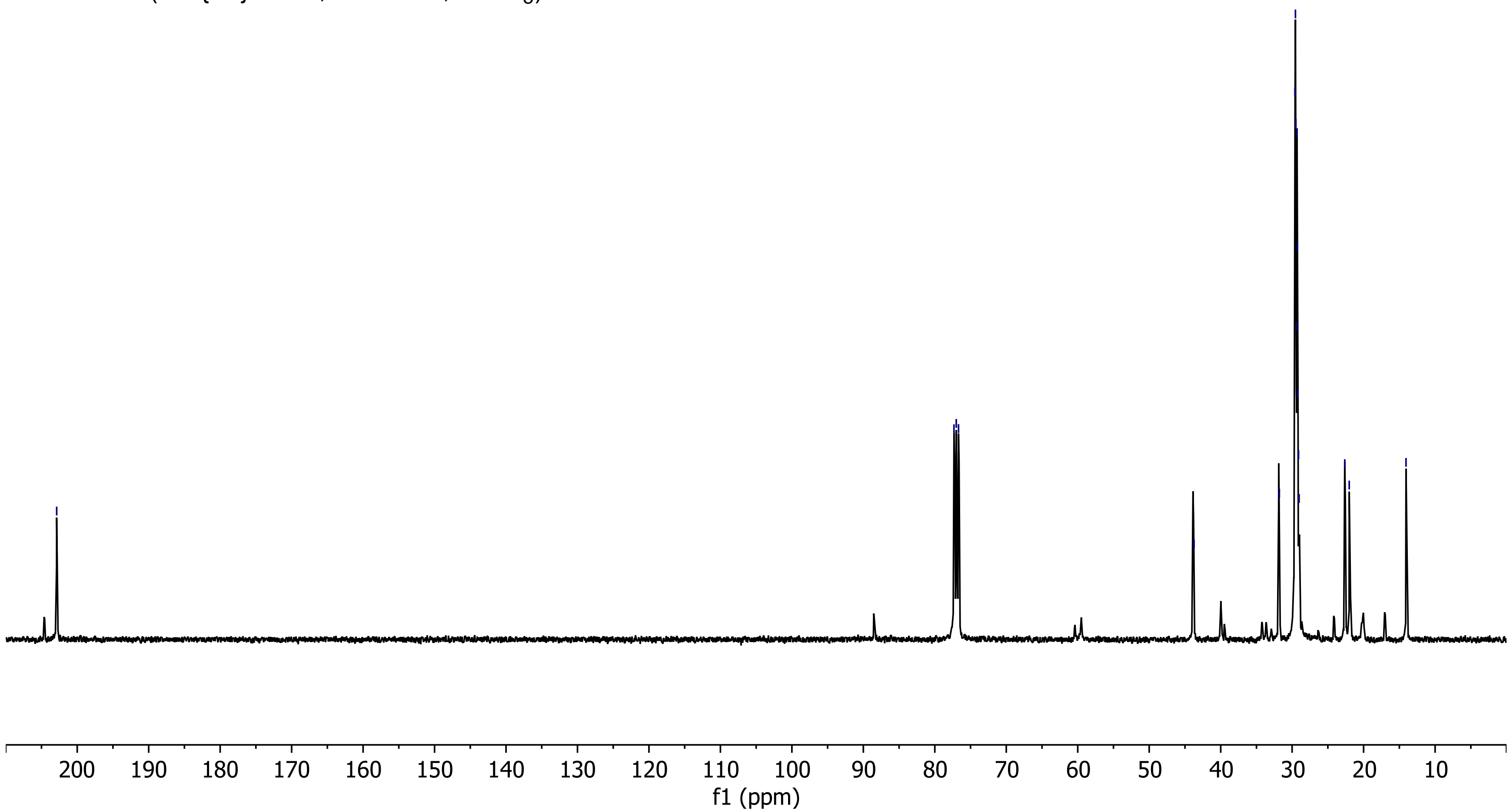

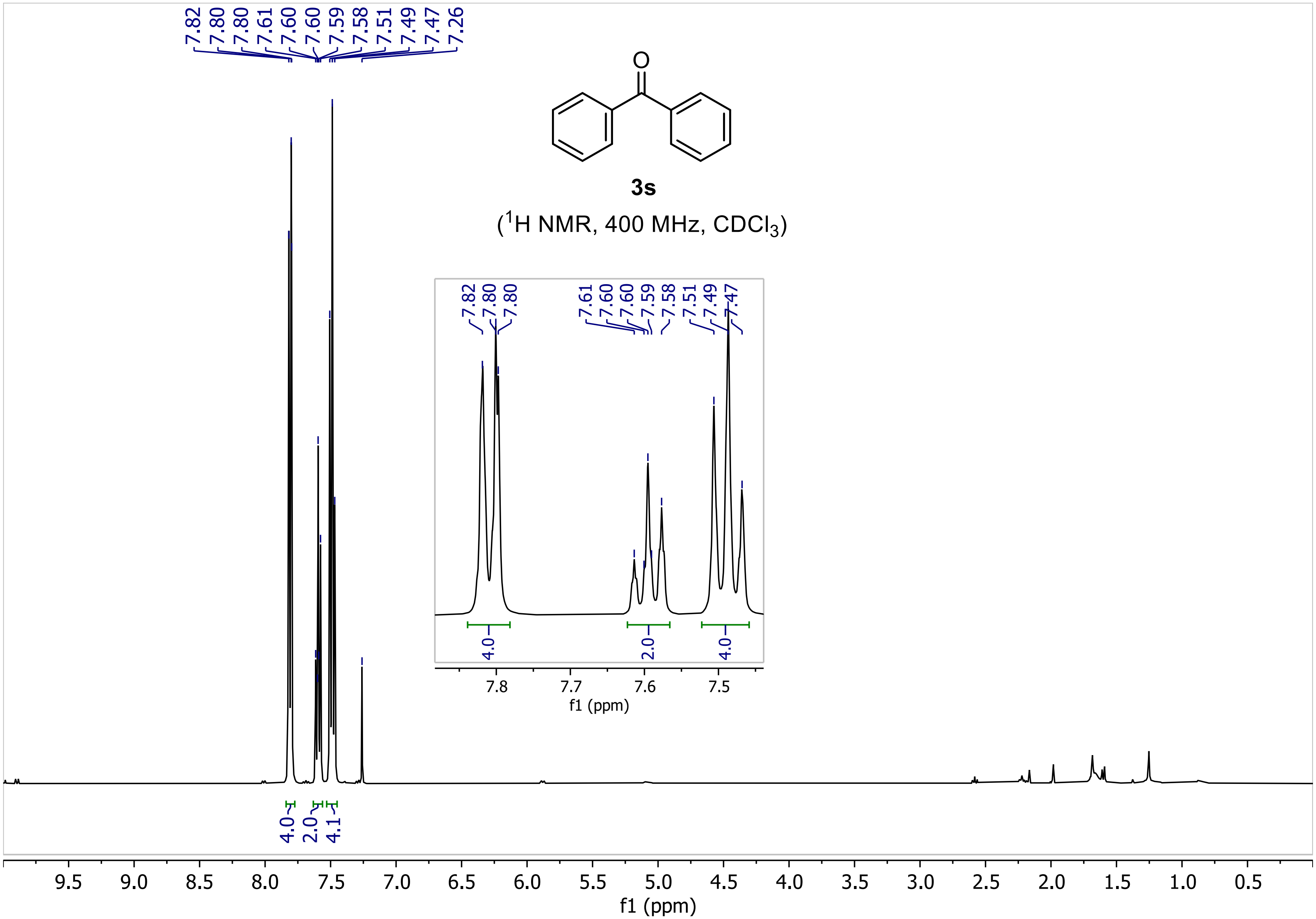

3s

(^{1}H NMR, 400 MHz, $CDCl_3$)

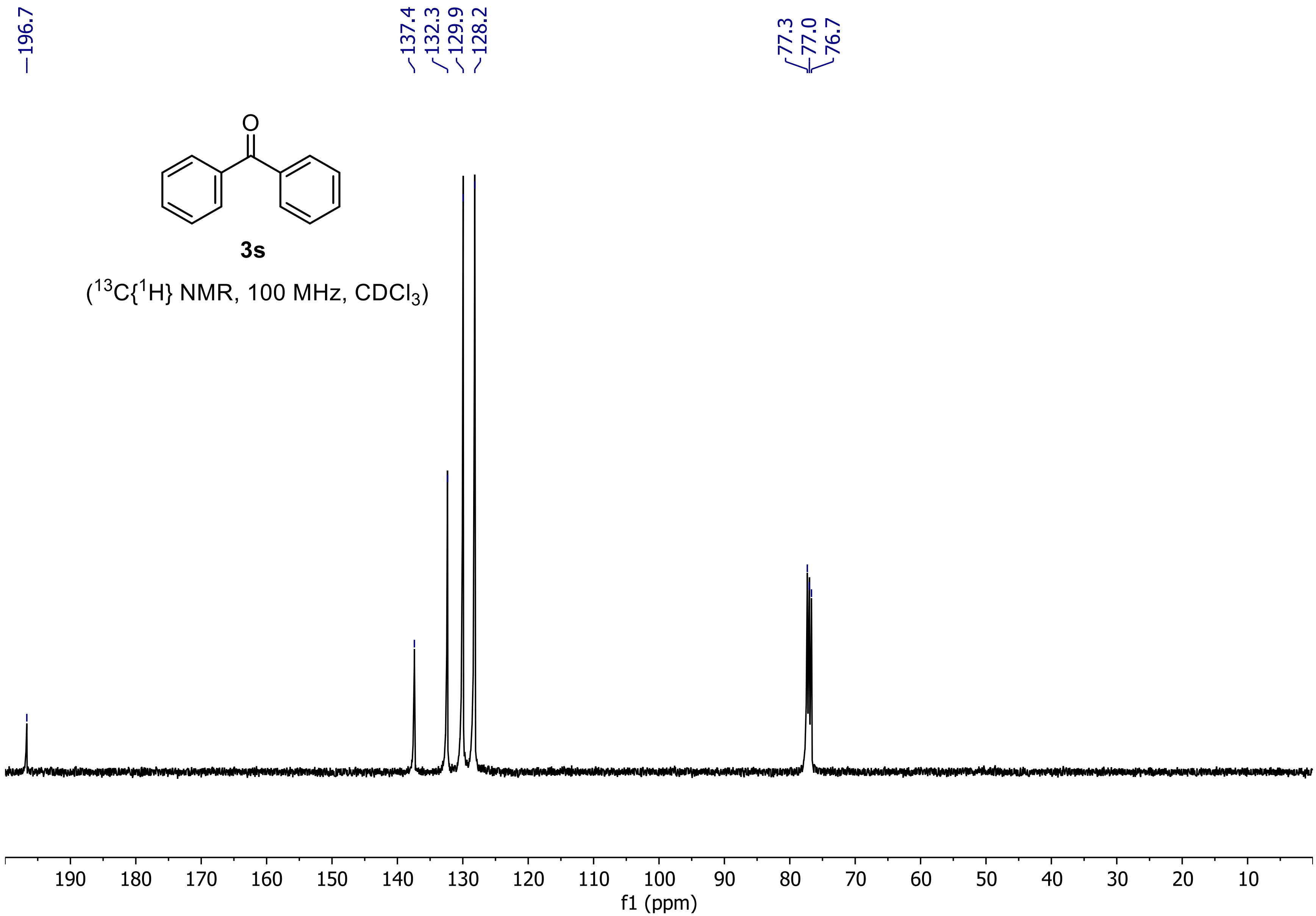

3s

($^{13}C\{^{1}H\}$ NMR, 100 MHz, $CDCl_3$)

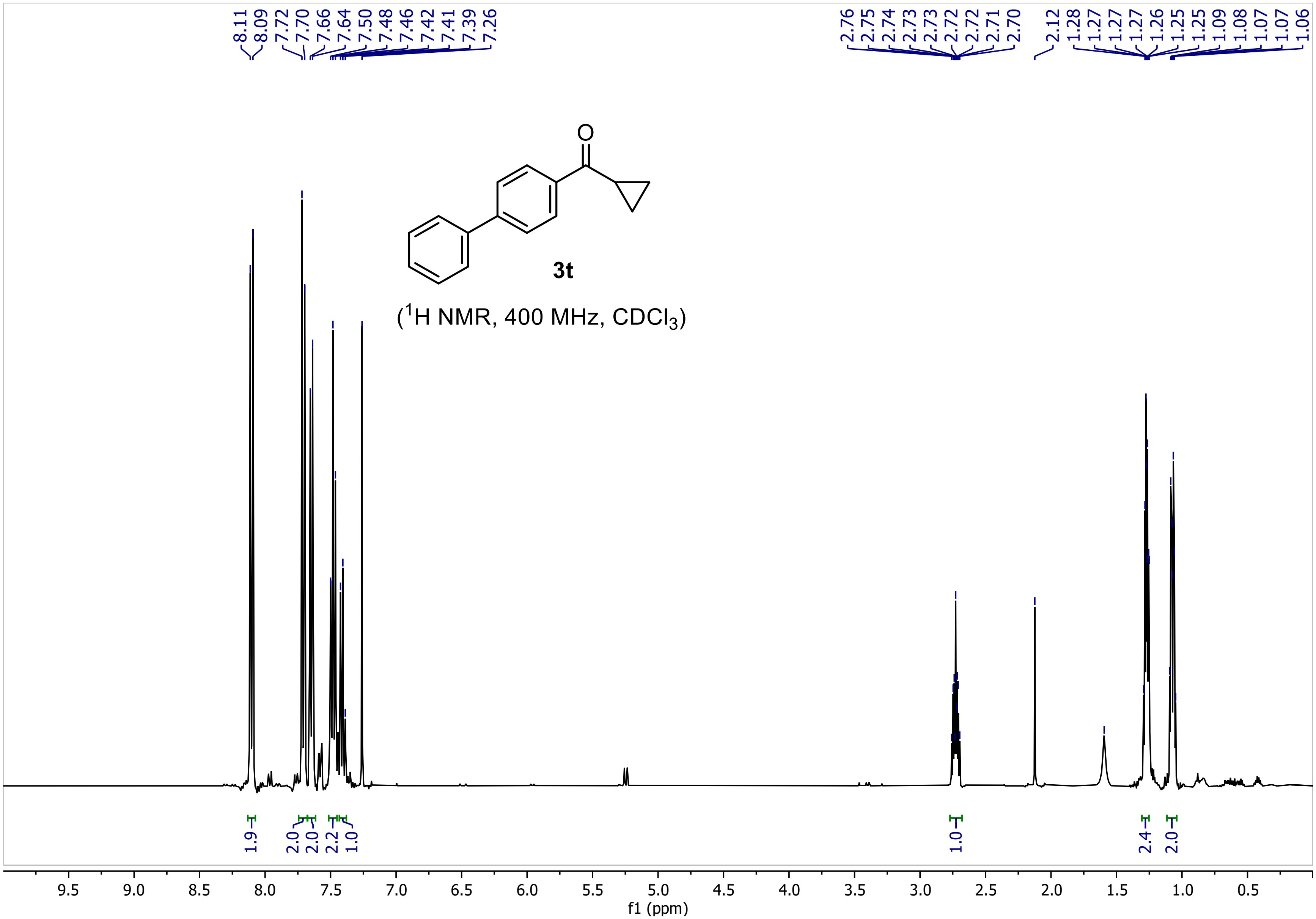
3t
(^{1}H NMR, 400 MHz, $CDCl_3$)
f1 (ppm)

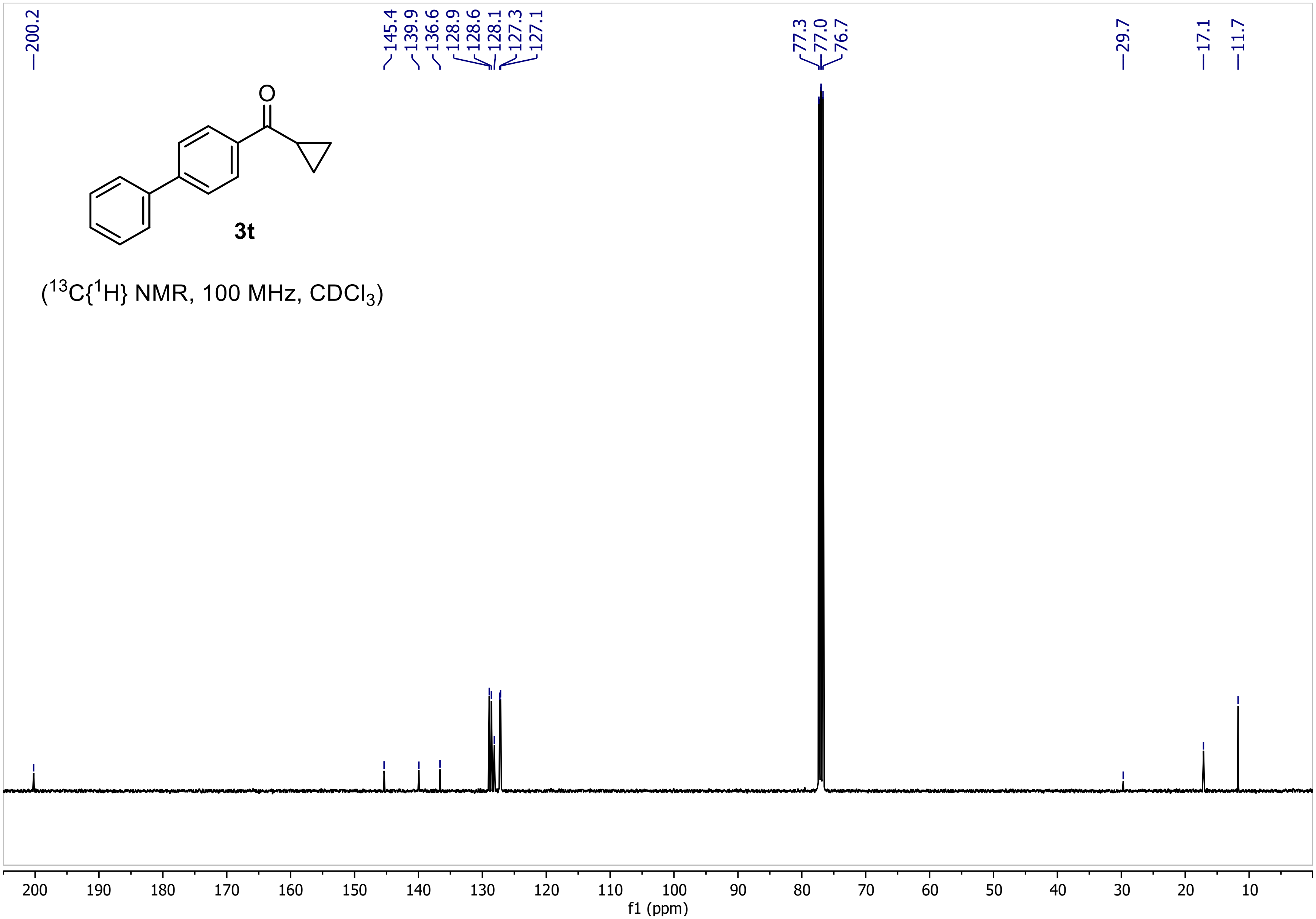
200.2
145.4
139.9
136.6
128.9
128.6
128.1
127.3
127.1
77.3
77.0
76.7
29.7
17.1
11.7
O
3t
($^{13}C\{^1H\}$ NMR, 100 MHz, $CDCl_3$)
200
190
180
170
160
150
140
130
120
110
100
90
80
70
60
50
40
30
20
10
f1 (ppm)
